# Psychometric properties of instruments for measuring abuse of older people in community and institutional settings: A systematic review

**DOI:** 10.1002/cl2.1419

**Published:** 2024-08-29

**Authors:** Fadzilah Hanum Mohd Mydin, Christopher Mikton, Wan Yuen Choo, Ranita Hisham Shunmugam, Aja Murray, Yongjie Yon, Raudah M. Yunus, Noran N. Hairi, Farizah M. Hairi, Marie Beaulieu, Amanda Phelan

**Affiliations:** ^1^ Department of Primary Care Medicine, Faculty of Medicine Universiti Malaya Kuala Lumpur Malaysia; ^2^ Department of Social Determinants of Health, Division of Healthier Populations World Health Organization Geneva Switzerland; ^3^ Department of Social & Preventive Medicine, Faculty of Medicine Universiti Malaya Kuala Lumpur Malaysia; ^4^ Department of Library & Information Science, Faculty of Arts & Social Sciences Universiti Malaya Kuala Lumpur Malaysia; ^5^ Department of Psychology University of Edinburgh Edinburg UK; ^6^ World Health Organization Regional Office for Europe Copenhagen Denmark; ^7^ Public Health Medicine Universiti Teknologi MARA Sungai Buloh Malaysia; ^8^ École de travail social, Faculté des lettres et sciences humaines Université de Sherbrooke Sherbrooke Québec Canada; ^9^ School of Nursing, Midwifery & Health Systems, National Centre for the Protection of Older People University College Dublin, Belfield Dublin Ireland

**Keywords:** abuse of older people, content validity, instrument, prevalence, psychometric properties

## Abstract

**Background:**

The examination of psychometric properties in instruments measuring abuse of older people (AOP) is a crucial area of study that has, unfortunately, received relatively little attention. Poor psychometric properties in AOP measurement instruments can significantly contribute to inconsistencies in prevalence estimates, casting a shadow of uncertainty over the magnitude of the problem at national, regional, and global levels.

**Objectives:**

This review rigorously employed the Consensus‐based Standards for the Selection of Health Measurement Instruments (COSMIN) guideline on the quality of outcome measures. It was designed to identify and review the instruments used to measure AOP, assess the instruments' measurement properties, and identify the definitions of AOP and abuse subtypes measured by these instruments, ensuring the reliability and validity of the findings.

**Search Methods:**

A comprehensive search was conducted up to May 2023 across various online databases, including AgeLine via EBSCOhost, ASSIA via ProQuest, CINAHL via EBSCOhost, EMBASE, LILACS, ProQuest Dissertation & Theses Global, PsycINFO via EBSCOhost, PubMed, SciELO, Scopus, Sociological Abstract via ProQuest, Chinese National Knowledge Infrastructure (CNKI), Google Scholar and WHO Global Index Medicus. Additionally, relevant studies were identified by thoroughly searching the grey literature from resources such as Campbell Collaboration, OpenAIRE, and GRAFT.

**Selection Criteria:**

All quantitative, qualitative (addressing face and content validity), and mixed‐method empirical studies published in peer‐reviewed journals or grey literature were included in this review. The included studies were primary studies that (1) evaluated one or more psychometric properties, (2) contained information on instrument development, or (3) examined the content validity of the instruments designed to measure AOP in community or institutional settings. The selected studies describe at least one psychometric property: reliability, validity, and responsiveness. Study participants represent the population of interest, including males and females aged 60 or older in community or institutional settings.

**Data Collection and Analysis:**

Two reviewers evaluated the screening of the selected studies' titles, abstracts, and full texts based on the preset selection criteria. Two reviewers assessed the quality of each study using the COSMIN Risk of Bias checklist and the overall quality of evidence for each psychometric property of the instrument against the updated COSMIN criteria of good measurement properties. Disagreements were resolved through consensus discussion or with assistance from a third reviewer. The overall quality of the measurement instrument was graded using a modified GRADE approach. Data extraction was performed using data extraction forms adapted from the COSMIN Guideline for Systematic Reviews of Outcome Measurement Instruments. The extracted data included information on the characteristics of included instruments (name, adaptation, language used, translation and country of origin), characteristics of the tested population, instrument development, psychometric properties listed in the COSMIN criteria, including details on content validity, structural validity, internal consistency, cross‐cultural validity/measurement invariance, reliability, measurement error, criterion validity, hypotheses testing for construct validity, responsiveness, and interoperability. All data were synthesised and summarised qualitatively, and no meta‐analysis was performed.

**Main Results:**

We found 15,200 potentially relevant records, of which 382 were screened in full text. A total of 114 studies that met the inclusion criteria were included. Four studies reported on more than one instrument. The primary reasons for excluding studies were their focus on instruments used solely for screening and diagnostic purposes, those conducted in hospital settings, or those without evaluating psychometric properties. Eighty‐seven studies reported on 46 original instruments and 29 studies on 22 modified versions of an original instrument. The majority of the studies were conducted in community settings (97 studies) from the perspective of older adults (90 studies) and were conducted in high‐income countries (69 studies). Ninety‐five studies assessed multiple forms of abuse, ranging from 2 to 13 different subscales; four studies measured overall abuse and neglect among older adults, and 14 studies measured one specific type of abuse. Approximately one‐quarter of the included studies reported on the psychometric properties of the most frequently used measurement instruments: HS‐EAST (assessed in 11 studies), VASS‐12 items (in 9 studies), and CASE (in 9 studies). The instruments with the most evidence available in studies reporting on instrument development and content validity in all domains (relevance, comprehensiveness and comprehensibility) were the DEAQ, OAPAM, *RAAL‐31 items, *ICNH (Norwegian) and OAFEM. For other psychometric properties, instruments with the most evidence available in terms of the number of studies were the HS‐EAST (11 studies across 5 of 9 psychometric properties), CASE (9 studies across 6 of 9 psychometric properties), VASS‐12 items (9 studies across 5 of 9 psychometric properties) and GMS (5 studies across 4 of 9 psychometric properties). Based on the overall rating and quality of evidence, the psychometric properties of the AOP measurement instruments used for prevalence measurement in community and institutional settings were insufficient and of low quality.

**Authors' Conclusions:**

This review aimed to assess the overall rating and quality of evidence for instruments measuring AOP in the community and institutional settings. Our findings revealed various measurement instruments, with ratings and evidence quality predominantly indicating insufficiency and low quality. In summary, the psychometric properties of AOP measurement instruments have not been comprehensively investigated, and existing instruments lack sufficient evidence to support their validity and reliability.

## 1 PLAIN LANGUAGE SUMMARY

Insufficient overall rating and low quality of evidence for the psychometric properties of measurement instruments for abuse of older people (AOP) in community and institutional settings.

### 1.1 The review in brief

The psychometric properties of the AOP measurement instruments used in community and institutional settings were insufficient, and the evidence was low‐quality. The evidence suggests that existing measurement instruments have poor validity and reliability.

### 1.2 What is this review about?

Various measurement instruments are used to estimate the prevalence of AOP in community and institutional settings; however, these estimates remain subject to uncertainty. Previous studies and systematic reviews investigating the prevalence of AOP have indicated that a potential factor contributing to this uncertainty could be the lack of a cross‐culturally valid and reliable measurement instrument. Therefore, this review aims to comprehensively evaluate the psychometric properties of the AOP measurement instruments in both community and institutional settings using the COSMIN guidelines. The summary of the overall rating and quality of evidence for the psychometric properties of each AOP measurement instrument are presented.

### 1.3 What is the aim of this review?

This Campbell systematic review examines the overall rating and quality of evidence of the psychometric properties of instruments used to measure AOP in community and institutional settings.

### 1.4 What are the main findings of this review?

#### 1.4.1 What studies are included?

This review included studies that evaluated the psychometric properties of the instruments used to measure AOP in community and institutional settings. The review synthesises the evidence from 114 studies across 68 instruments administered to older adults, caregivers or third persons (e.g., nursing staff, health care providers). Studies included in this review were published between 1986 and 2023. Only 21 studies across 20 instruments reported on instrument development, and 34 studies reported the content validity of the 25 instruments. Internal consistency was the most frequently assessed psychometric property among the included instruments (89 studies, 56 instruments), followed by structural validity (42 studies, 28 instruments) and hypothesis testing/construct validity (32 studies, 20 instruments). A few instruments evaluated criterion validity (five studies, four instruments), measurement invariance (two studies, two instruments) and cross‐cultural validity (one study, one instrument). Measurement errors and responsiveness were not evaluated in any of the studies. Most studies included in this review were conducted in high‐income countries (69 studies). All the included studies have significant methodological weaknesses.

#### 1.4.2 Are the psychometric properties of instruments used to measure the prevalence of AOP sufficient, and does high‐quality evidence support them?

The low‐quality evidence reported in this review demonstrated that most of the included instruments required additional evidence for their psychometric properties. This underscores the necessity for further research to rigorously evaluate the psychometric properties of existing measurement instruments using appropriate methodological approaches. Alternatively, developing a new measurement instrument and rigorously testing its psychometric properties may be imperative.

#### 1.4.3 Are there any instruments recommended for AOP measurement?

There is no specific instrument we would recommend as a valid and reliable instrument to measure the prevalence of AOP in community and institutional settings.

### 1.5 What do the findings of this review mean?

This review reveals various instruments employed to measure AOP's prevalence in community and institutional settings. However, the psychometric properties of these AOP measurement instruments have not received sufficient research attention. Evidence from existing studies indicates that the overall ratings for the evaluated psychometric properties are insufficient, supported by low‐quality evidence. There is a clear need for well‐structured research endeavours to comprehensively evaluate the psychometric properties of existing measurement instruments or develop and evaluate new instruments.

### 1.6 How up‐to‐date is this review?

The literature search was conducted up to May 2023.

## 2 SUMMARY OF FINDINGS

See Table 1.

**Table 1:** Summary of findings.

		Content validity		Relevance		Comprehensiveness		Comprehensibility		Structural validity		Internal consistency		Cross‐cultural validity		Measurement invariance		Reliability		Criterion validity		Hypothesis testing	
No	Instrument	Overall rating	Quality of evidence	Overall rating	Quality of evidence	Overall rating	Quality of evidence	Overall rating	Quality of evidence	Overall rating	Quality of evidence	Overall rating	Quality of evidence	Overall rating	Quality of evidence	Overall rating	Quality of evidence	Overall rating	Quality of evidence	Overall rating	Quality of evidence	Overall rating	Quality of evidence
COMMUNITY																							
1.	OAPAM	+	Moderate	+	Moderate	+	Moderate	+	Moderate	+	High	+	High	NE	NE	NE	NE	NE	NE	NE	NE	+	High
2.	GMS	+	Moderate	+	Moderate	+	Moderate	+	Moderate	±	Inconsistent	?	Very Low	NE	NE	NE	NE	+	Very Low	NE	NE	+	Moderate
3.	UKNPS	+	Moderate	+	Moderate	+	Moderate	+	Moderate	NE	NE	NE	NE	NE	NE	NE	NE	NE	NE	NE	NE	NE	NE
4.	DEAQ	+	Low	+	Low	+	Low	+	Low	+	High	+	High	NE	NE	NE	NE	?	Low	NE	NE	NE	NE
5.	HS‐EAST	+	Low	+	Low	?	Low	+	Low	±	Inconsistent	?	Very Low	NE	NE	NE	NE	?	Low	±	Inconsistent	+	High
6.	VASS‐12 items	+	Low	‐	Low	+	Low	±	Low	+	High	+	Moderate	NE	NE	NE	NE	?	Very Low	‐	High	+	High
7.	IEAQ‐Long form	‐	Low	‐	Low	‐	Low	‐	Low	+	High	‐	High	NE	NE	NE	NE	NE	NE	NE	NE	NE	NE
8.	mCTS‐34 items	‐	Low	‐	Low	‐	Low	‐	Low	NE	NE	NE	NE	NE	NE	NE	NE	NE	NE	NE	NE	+	High
9.	ATDEA	‐	Low	+	Low	‐	Low	‐	Low	NE	NE	?	Very Low	NE	NE	NE	NE	NE	NE	NE	NE	NE	NE
10.	CASE	±	Inconsistent	‐	Low	+	Moderate	+	Moderate	+	Moderate	+	Moderate	+	Moderate	+	High	±	Inconsistent	NE	NE	+	High
11.	*NAS (Ghana)	±	Inconsistent	±	Low	+	Low	‐	Low	+	High	+	High	NE	NE	NE	NE	NE	NE	NE	NE	+	High
12.	mGMS (Neglect)	NE	NE	+	Moderate	+	Moderate	NE	NE	+	High	+	High	NE	NE	NE	NE	?	Very Low	NE	NE	+	High
13.	MEAS	NE	NE	‐	Low	‐	Low	NE	NE	+	High	‐	High	NE	NE	NE	NE	NE	NE	NE	NE	+	High
14.	FIVE	NE	NE	‐	Low	+	Low	NE	NE	+	High	‐	High	NE	NE	NE	NE	NE	NE	NE	NE	+	High
15.	*EA (Korea)	NE	NE	‐	Low	‐	Low	NE	NE	NE	NE	?	Very Low	NE	NE	NE	NE	NE	NE	NE	NE	NE	NE
16.	EAS	NE	NE	‐	Low	‐	Low	NE	NE	NE	NE	NE	NE	NE	NE	NE	NE	?	Very Low	NE	NE	NE	NE
17.	*IPPA (India)	NE	NE	‐	Very Low	‐	Very Low	NE	NE	+	High	+	Very Low	NE	NE	NE	NE	NE	NE	NE	NE	NE	NE
18.	EMM	NE	NE	‐	Very Low	NE	NE	NE	NE	?	High	‐	High	NE	NE	NE	NE	NE	NE	NE	NE	NE	NE
19.	FVOW	NE	NE	‐	Very Low	NE	NE	NE	NE	‐	High	+	Very Low	NE	NE	NE	NE	NE	NE	NE	NE	?	High
20.	EACS	NE	NE	‐	Very Low	NE	NE	NE	NE	+	High	+	High	NE	NE	NE	NE	NE	NE	NE	NE	+	High
21.	*mRAAL‐27 items	NE	NE	NE	NE	NE	NE	+	Low	NE	NE	NE	NE	NE	NE	NE	NE	NE	NE	NE	NE	NE	NE
22.	mCTS‐M 38 items	NE	NE	NE	NE	NE	NE	NE	NE	+	High	+	High	NE	NE	NE	NE	NE	NE	NE	NE	+	Very Low
23.	Native EAS‐Short Form	NE	NE	NE	NE	NE	NE	NE	NE	+	High	+	High	NE	NE	NE	NE	NE	NE	NE	NE	+	High
24.	mCTS‐C 25 Items	NE	NE	NE	NE	NE	NE	NE	NE	NE	NE	?	Moderate	NE	NE	NE	NE	NE	NE	NE	NE	+	High
25.	mCTS‐10 items	NE	NE	NE	NE	NE	NE	NE	NE	NE	NE	?	Very Low	NE	NE	NE	NE	NE	NE	+	High	NE	NE
26.	mVASS‐15 items	NE	NE	NE	NE	NE	NE	NE	NE	+	High	+	High	NE	NE	NE	NE	NE	NE	NE	NE	NE	NE
27.	*mNSEAN	NE	NE	NE	NE	NE	NE	NE	NE	NE	NE	?	Very Low	NE	NE	NE	NE	NE	NE	NE	NE	+	High
28.	*mNSEAN (Neglect)*	NE	NE	NE	NE	NE	NE	NE	NE	NE	NE	?	High	NE	NE	+	High	NE	NE	NE	NE	NE	NE
29.	mOARS‐ADL (Neglect)	NE	NE	NE	NE	NE	NE	NE	NE	NE	NE	?	High	NE	NE	NE	NE	NE	NE	NE	NE	+	High
30.	EVEQ	NE	NE	NE	NE	NE	NE	NE	NE	‐	High	+	High	NE	NE	NE	NE	NE	NE	NE	NE	NE	NE
31.	FVS	NE	NE	NE	NE	NE	NE	NE	NE	+	High	+	Very low	NE	NE	NE	NE	NE	NE	NE	NE	NE	NE
32.	*EAN (Puerto Ricans)	NE	NE	NE	NE	NE	NE	NE	NE	?	High	+	High	NE	NE	NE	NE	NE	NE	NE	NE	NE	NE
33.	EMS	NE	NE	NE	NE	NE	NE	NE	NE	+	Very Low	+	Very Low	NE	NE	NE	NE	NE	NE	NE	NE	NE	NE
34.	WHRS	NE	NE	NE	NE	NE	NE	NE	NE	?	Very Low	?	High	NE	NE	NE	NE	NE	NE	NE	NE	NE	NE
35.	*EANQ (Iran)	NE	NE	NE	NE	NE	NE	NE	NE	NE	NE	?	Very Low	NE	NE	NE	NE	?	Very Low	NE	NE	NE	NE
36.	mCTS‐T 18 items	NE	NE	NE	NE	NE	NE	NE	NE	NE	NE	?	High	NE	NE	NE	NE	NE	NE	NE	NE	NE	NE
37.	mCTS‐C 18 items	NE	NE	NE	NE	NE	NE	NE	NE	NE	NE	?	High	NE	NE	NE	NE	NE	NE	NE	NE	NE	NE
38.	mCTS‐C 23 items	NE	NE	NE	NE	NE	NE	NE	NE	NE	NE	?	High	NE	NE	NE	NE	NE	NE	NE	NE	NE	NE
39.	mCTS‐Verbal 12 items	NE	NE	NE	NE	NE	NE	NE	NE	NE	NE	?	Very Low	NE	NE	NE	NE	NE	NE	NE	NE	NE	NE
40.	mVASS‐10 items	NE	NE	NE	NE	NE	NE	NE	NE	NE	NE	?	Very Low	NE	NE	NE	NE	NE	NE	NE	NE	NE	NE
41.	mVASS‐16 items	NE	NE	NE	NE	NE	NE	NE	NE	NE	NE	?	Very Low	NE	NE	NE	NE	NE	NE	NE	NE	NE	NE
42.	m‐HS‐EAST	NE	NE	NE	NE	NE	NE	NE	NE	NE	NE	?	Very Low	NE	NE	NE	NE	NE	NE	NE	NE	NE	NE
43.	FPS‐2 items	NE	NE	NE	NE	NE	NE	NE	NE	NE	NE	NE	NE	NE	NE	NE	NE	NE	NE	‐	High	NE	NE
44.	mFPS‐5 items	NE	NE	NE	NE	NE	NE	NE	NE	NE	NE	NE	NE	NE	NE	NE	NE	NE	NE	NE	NE	?	Moderate
45.	ABUEL	NE	NE	NE	NE	NE	NE	NE	NE	NE	NE	?	High	NE	NE	NE	NE	NE	NE	NE	NE	NE	NE
46.	*DVE (Thailand)	NE	NE	NE	NE	NE	NE	NE	NE	NE	NE	?	Very Low	NE	NE	NE	NE	NE	NE	NE	NE	NE	NE
47.	*EAQ (Nepal)	NE	NE	NE	NE	NE	NE	NE	NE	NE	NE	?	Very Low	NE	NE	NE	NE	NE	NE	NE	NE	NE	NE
48.	EAVQ	NE	NE	NE	NE	NE	NE	NE	NE	NE	NE	?	High	NE	NE	NE	NE	NE	NE	NE	NE	NE	NE
49.	USCOACS	NE	NE	NE	NE	NE	NE	NE	NE	NE	NE	?	High	NE	NE	NE	NE	NE	NE	NE	NE	NE	NE
50.	*IPVQ	NE	NE	NE	NE	NE	NE	NE	NE	?	High	NE	NE	NE	NE	NE	NE	NE	NE	NE	NE	NE	NE
51.	*EAQ (China)	NE	NE	NE	NE	NE	NE	NE	NE	NE	NE	NE	NE	NE	NE	NE	NE	+	Very Low	NE	NE	NE	NE
52.	*EAQ (Iran)	NE	NE	NE	NE	NE	NE	NE	NE	NE	NE	?	Very Low	NE	NE	NE	NE	NE	NE	NE	NE	NE	NE
53.	m‐EAQ	NE	NE	NE	NE	NE	NE	NE	NE	NE	NE	?	High	NE	NE	NE	NE	NE	NE	NE	NE	NE	NE
54.	m‐EASI	NE	NE	NE	NE	NE	NE	NE	NE	NE	NE	?	Very Low	NE	NE	NE	NE	NE	NE	NE	NE	NE	NE
55.	QEEA	NE	NE	NE	NE	NE	NE	NE	NE	NE	NE	?	Very Low	NE	NE	NE	NE	NE	NE	NE	NE	NE	NE
56.	AAT items	NE	NE	NE	NE	NE	NE	NE	NE	NE	NE	?	Very Low	NE	NE	NE	NE	NE	NE	NE	NE	NE	NE
INSTITUTION																							
57.	R‐REM	+	Moderate	+	Moderate	+	Moderate	+	Moderate	+	High	+	High	NE	NE	NE	NE	NE	NE	NE	NE	NE	NE
58.	*ICNH (Norwegian)	±	Inconsistent	+	Low	‐	Low	+	Low	‐	Moderate	+	High	NE	NE	NE	NE	NE	NE	NE	NE	NE	NE
59.	*RAAL‐31 items	+	Low	+	Low	+	Low	+	Low	NE	NE	NE	NE	NE	NE	NE	NE	NE	NE	NE	NE	NE	NE
60.	*mRAAL‐28 items	+	Low	+	Low	+	Low	+	Low	NE	NE	?	Very Low	NE	NE	NE	NE	NE	NE	NE	NE	NE	NE
61.	CHCS	NE	NE	+	High	+	Low	NE	NE	NE	NE	NE	NE	NE	NE	NE	NE	NE	NE	NE	NE	NE	NE
62.	DVSQ	NE	NE	+	Very Low	‐	Very Low	NE	NE	NE	NE	NE	NE	NE	NE	NE	NE	NE	NE	NE	NE	NE	NE
63.	*EAS (Korea)	NE	NE	‐	Low	‐	Low	NE	NE	+	High	+	Low	NE	NE	NE	NE	NE	NE	NE	NE	NE	NE
64.	CPEAB	NE	NE	‐	Low	‐	Low	NE	NE	NE	NE	?	High	NE	NE	NE	NE	‐	Very low	NE	NE	NE	NE
65.	*mICNH (Norwegian)	NE	NE	NE	NE	NE	NE	NE	NE	NE	NE	?	High	NE	NE	NE	NE	NE	NE	NE	NE	NE	NE
66.	*EAQ (Japan)	NE	NE	NE	NE	NE	NE	NE	NE	NE	NE	?	High	NE	NE	NE	NE	NE	NE	NE	NE	NE	NE
67.	*EAQ (Japan)	NE	NE	NE	NE	NE	NE	NE	NE	NE	NE	?	High	NE	NE	NE	NE	NE	NE	NE	NE	NE	NE
COMMUNITY AND INSTITUITION																							
68.	OAFEM	+	Moderate	+	Moderate	+	Moderate	+	Moderate	+	High	+	High	NE	NE	NE	NE	NE	NE	NE	NE	+	High
69.	EPAS	NE	NE	+	Moderate	+	Moderate	NE	NE	NE	NE	?	High	NE	NE	NE	NE	+	Low	NE	NE	+	High

## 3 BACKGROUND

### 3.1 The problem, condition or issue

AOP (widely recognised as elder abuse and neglect) is now acknowledged as a prevalent and growing issue with profound consequences for the health and social well‐being of older people. AOP is ‘a single or repeated act, or lack of appropriate action, occurring within any relationship with an expectation of trust, which causes harm or distress to an older person’. This type of violence violates human rights and includes physical, sexual, psychological and emotional abuse; financial and material abuse; abandonment; neglect; and serious loss of dignity and respect’ (World Health Organization, 2002). It also involves deliberate or neglectful acts by the older person's formal or informal caregiver or trusted individual that cause harm to a vulnerable older person. AOP can occur in various settings, from home to institutional settings and within the broader community. The definitions of different AOP subtypes are provided in Supporting Information: Appendix 1.

AOP is a complex issue. Several risk factors can increase the likelihood of older adults experiencing abuse, including social isolation, cognitive impairment, physical dependence, mental health issues, substance abuse, history of abuse, financial exploitation, low income and socioeconomic status, gender (i.e., being a woman), racial/ethnic group and financial dependence (Johannesen & LoGiudice, 2013; Li et al., 2020; Pillemer et al., 2016; Storey, 2020; Yan et al., 2015). Perpetrator risk factors include poor psychological health, substance misuse, and dependency on the abuser. In addition, other older adult characteristics, such as relationship dynamics and marital status, may also contribute to the risk of AOP.

There are notable distinctions in the AOP occurrences between community and institutional settings, including differences in the types of abuse, perpetrator profiles, and reporting procedures. The AOP occurs in the home or community and is mainly perpetrated by their spouses, family members or caregivers responsible for their care. On the contrary, abuse in institutional settings is predominantly perpetrated by professional caregivers or peers (Yon, Mikton, et al., 2019; Yon, Ramiro‐Gonzalez, et al., 2019). AOP in both community and institutional settings can manifest in various forms, including physical, emotional, financial, sexual and neglect. Additionally, within institutional settings, other types of abuse may also occur, such as medication errors, over‐medication or the use of restraints. Reporting AOP in the community is also challenging due to social isolation or fear of reporting. In institutions, the requirement for staff members to report suspected abuse to relevant authorities varies widely depending on each country's legal and regulatory framework.

In the context of a rapidly ageing global population, the issue of AOP urgently requires attention and intervention from healthcare providers, social welfare agencies, and policymakers. Data from research worldwide suggest that AOP is often underreported in many countries. The prevalence of AOP has been investigated through population surveys where older adults or their proxies are surveyed directly to collect information about their experiences, including exposure frequency and specific types of abuse (Sooryanarayana et al., 2013; Yon et al., 2017; Yon, Mikton, et al., 2019; Yon, Ramiro‐Gonzalez, et al., 2019).

A recent systematic review estimated that the prevalence of AOP in the community was 15.7% over the past year based on population surveys (Yon et al., 2017). In institutional settings such as nursing homes and other long‐term care facilities, there was insufficient data to estimate the prevalence reported by older adults. Still, findings found that approximately two in three staff members working in nursing homes admitted to perpetrating AOP in the past year (Yon, Ramiro‐Gonzalez, et al., 2019). The significant drawbacks to the quantified estimates in these reviews were the lack of comparable data due to heterogeneous methods and the fact that most included studies were from high‐income countries. Consequently, robust prevalence studies in low‐ and middle‐income countries are deficient. However, over the past 10 years, considerable research on measuring AOP has emerged from developing countries such as Malaysia, Iran, Brazil and India. Therefore, it is an opportunity to conduct a new review of AOP measurement instruments (Blay et al., 2017; Nassiri et al., 2016; Patel et al., 2018; Sooryanarayana et al., 2013). Research findings from countries worldwide may help identify similarities and differences in AOP measurement instruments. A comprehensive review of the psychometric properties of the measurement instruments utilised in prevalence studies can help identify gaps and provide relevant recommendations for AOP measurement instruments.

It is crucial to assess the burden of AOP in a population, compare the prevalence of AOP in different populations to identify risk factors and examine trends in planning and evaluating strategies, policies or large‐scale interventions. However, the literature has demonstrated a wide variation in reported prevalence rates due to methodological differences and a lack of agreement on defining and measuring AOP and its subtypes (Sooryanarayana et al., 2013; World Health Organization, 2022a; Yan et al., 2015; Yon et al., 2017; Yon, Mikton, et al., 2019; Yon, Ramiro‐Gonzalez, et al., 2019; Zhang et al., 2022). This issue is not limited to AOP but is a common problem researchers face when measuring exposure to other types of violence, such as child maltreatment and interpersonal violence (Alhabib et al., 2010; Mathews et al., 2020). In addition, reports have also documented extensive cultural variation in the circumstances and context of AOP (Lee et al., 2014; Li et al., 2020; Zhang, 2019). Most prevalence studies have utilised the widely accepted definition of AOP and its subtypes as adopted by the World Health Organisation and the United States Centres for Disease Control and Prevention (Hall et al., 2016; Sooryanarayana et al., 2013; Yon et al., 2017; Yon, Ramiro‐Gonzalez, et al., 2019). Besides this overarching framework, there are various words used interchangeably to describe the phenomenon, such as ‘harm’, ‘exploitation’, ‘mistreatment’, ‘maltreatment’, and ‘violence’ found in the literature. To what extent researchers adopted these terms or whether standardised (or non‐standardised) instruments were used to measure ‘abuse’ in these studies remains unclear. Although there is no gold standard measurement instrument to establish the prevalence of AOP in community or institutional settings, many AOP measurement instruments have emerged over the past decades.

Despite the availability of several AOP measurement instruments, most studies only document part of the psychometric properties of these instruments (Cooper, Manela, et al., 2008; Jackson, 2018; Sooryanarayana et al., 2013; Yan et al., 2015; Yon et al., 2017; Yon, Ramiro‐Gonzalez, et al., 2019; Zhang et al., 2022). Besides, adapting an existing AOP instrument to be used in another population is a common practice, yet information regarding the cross‐cultural validity of the instrument remains sparse. The psychometric properties of an instrument are population‐specific, and its validity and reliability are not generalised. Information regarding the adaptation process is essential when an instrument is used in a different gender, community, language, setting or time to avoid introducing bias into the study. Measurement instruments should also be evaluated for cultural suitability in a new context, including conducting cognitive interviews with the relevant population and assessing their cross‐cultural validity (Prinsen et al., 2018; Terwee et al., 2018).

Prevalence studies relying on poor or uncertain‐quality measurement instruments can potentially yield misleading findings. Understanding the current state of research in this context reveals the critical need for a comprehensive evaluation of these instruments. Such assessments highlight how their measurement properties were determined and existing gaps and limitations. This knowledge is crucial for researchers in this field, providing them with the insights necessary to select measurement instruments with demonstrated validity and reliability for future studies, further investigate psychometric properties in areas of deficiency or develop a new instrument when the psychometric properties of existing instruments are deemed inadequate.

### 3.2 Description of the phenomena of interest

The psychometric properties of AOP measurement instruments used in existing prevalence surveys have not received sufficient attention (Cooper, Manela, et al., 2008; Sooryanarayana et al., 2013; Yan et al., 2015; Yon et al., 2017; Yon, Ramiro‐Gonzalez, et al., 2019; Zhang et al., 2022). Selecting the best measurement instruments for AOP prevalence studies requires an instrument supported by evidence of reliability, validity, cross‐cultural validity, and responsiveness (Prinsen et al., 2018; Terwee et al., 2018). Evaluating the psychometric properties of existing measurement instruments can identify gaps in the knowledge of psychometric evidence, guide the development of new AOP measurement instruments, and test their psychometric properties.

This systematic review utilised the Consensus‐based Standards for the selection of health Measurement Instruments (COSMIN) methodology to systematically review the psychometric properties of AOP measurement instruments (Prinsen et al., 2018). COSMIN methodology provides a comprehensive checklist to assess the quality and criteria for the good measurement properties of the instruments utilised for research and practice. The COSMIN taxonomy of psychometric properties is based on three domains: reliability, validity and responsiveness. The first domain, focusing on the reliability of the measurement instrument scores, comprises internal consistency, reliability (test–retest, inter‐rater and intra‐rater) and measurement error (test–retest, inter‐rater and intra‐rater). The second domain, which pertains to the validity of the measurement instrument, includes content validity (relevance, comprehensiveness or comprehensibility including face validity), structural validity, hypotheses testing for construct validity, cross‐cultural validity and criterion validity. The third domain addresses the responsiveness of measurement instruments, defined by their abilities to detect changes in response to interventions.

The COSMIN Manual for Systematic Reviews of Patient‐Reported Outcome Measures (Prinsen et al., 2018) provides comprehensive definitions of each domain.

### 3.3 Why it is important to do this review

AOP is a serious public health and social problem expected to escalate over time (Pillemer et al., 2016). Recognising this, both the World Health Organization's Global Action Plan on Ageing and Health Strategy and the United Nations Decade of Healthy Aging (2021–2030) outline the need to establish the prevalence of AOP and implement evidence‐informed AOP prevention and response programs (United Nations, 2015). This commitment aligns with the 2030 Agenda for Sustainable Developmental Goal (SDG), which strongly emphasises human rights within all categories of age in society, focusing on vulnerable populations, including older people, aiming to end discrimination later in life (Lee et al., 2016). Accurate and reliable data is needed for older people to fulfil the SDG indicator (SDG 16.1.3) that specifically targets the proportion of the population in each country subjected to various forms of abuse, including physical, psychological, financial and sexual abuse across all ages, including older people. This indicator is a critical tool in addressing the unique challenges older individuals face and promotes their well‐being within a broader societal context.

In addition, ‘Tackling abuse of older people: Five priorities for the United Nations Decade of Healthy Ageing (2021–2030)’ identifies generating more and better data on prevalence as one of the five priorities for the field (World Health Organization, 2022b). There is little data on the prevalence of AOP, particularly in low‐ and middle‐income countries and institutions, and the accuracy of the available estimates has been questioned. Understanding prevalence is the basis for communicating the scale of a problem, where the intervention is most needed and facilitating investigations on the effective interventions using a cross‐setting comparative approach. Furthermore, the report proposes the development of an instrument for measuring AOP based on the best existing instruments identified in systematic reviews of the quality of the measurement instruments.

Based on an initial search, we uncovered a systematic review of survey instruments used to measure staff‐to‐resident AOP in residential care settings (Malmedal et al., 2020). However, this review excluded studies of AOP in the community. There is also a narrative review of instruments used to measure violence against older women (unpublished) (Mikton, 2019). Jackson (2018) has reviewed financial exploitation among older people (Jackson, 2018). On a related note, several other reviews have focused on AOP screening and detection instruments used by service providers in healthcare settings and home environments (Gallione et al., 2017; McCarthy et al., 2017; Van Royen et al., 2020). Though these screening and detection instruments are critical for service providers to detect and respond to potential cases of abuse, it is essential to note that they may not be optimally suited for estimating the prevalence of AOP, particularly in community settings. Consequently, there is currently a notable gap in the literature on the quality and psychometric properties of instruments and the definitions used to measure abuse against older men and older women in both community and institutional settings (Gallione et al., 2017; Malmedal et al., 2020). The lack of clarity on the quality of existing instruments has resulted in significant uncertainty regarding the global prevalence of AOP.

A crucial step in addressing this gap is systematically reviewing the psychometric properties of AOP measurement instruments using the rigorous procedure recommended in the Consensus‐based Standards for the Selection of Health Measurement Instruments (COSMIN) methodology (Mokkink et al., 2018). This review examines the definitions and items used to measure the prevalence of AOP in community or institutional settings (Prinsen et al., 2018; Terwee et al., 2018).

A comprehensive and high‐quality systematic review enhances evidence‐based recommendations for appropriate instruments for measuring AOP worldwide. The findings of this review will help identify or develop a standardised, reliable, valid, and cross‐culturally valid approach to measure AOP in the community or institutional settings. This enables practitioners and policymakers to make informed choices when selecting an instrument to measure AOP and employ an evidence‐informed approach for assessing AOP initiatives in the future. It also contributes to clearing the uncertainty about global, regional, and national prevalence estimates.

## 4 OBJECTIVES

Our research questions are:

1. What are the psychometric properties of instruments to measure the prevalence of AOP in the community or institutional setting?
2. What is the quality of the evidence supporting the psychometric properties of AOP measurement instruments?
3. What is the definition of AOP used in the AOP measurement instruments?
4. What are the abuse subtypes measured in the AOP measurement instruments?

An exhaustive review was conducted, including identifying, gathering, critical appraisal, comparing, and describing all current instruments used to measure AOP in community or institutional settings, along with their respective psychometric properties. We also identified the definitions and domains of AOP incorporated in these measurement instruments. Based on our findings, we identified all instruments used worldwide in prevalence studies of AOP and determined their comprehensiveness in measuring AOP.

## 5 METHODS

**Figure 1:**
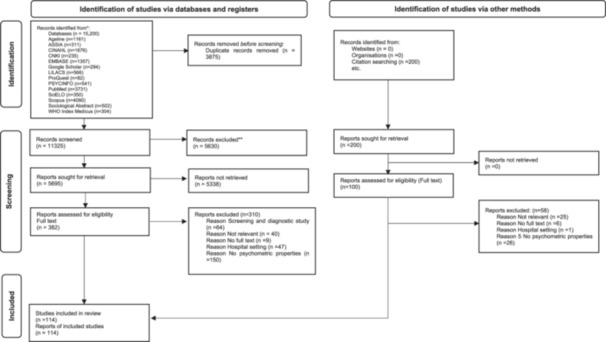
PRISMA Flow Chart. Consider, if feasible to do so, reporting the number of records identified from each database or register searched (rather than the total number across all databases/registers). **If automation tools were used, indicate how many records were excluded by a human and how many were excluded by automation tools. *Source:* Page et al. (2021). For more information, visit: http://www.prisma-statement.org/.

This systematic review follows the Consensus‐based Standards for Selecting Health Measurement Instrument (COSMIN) Guidelines for Systematic Reviews of Patient Reported Outcome Measures(Prinsen et al., 2018; Terwee et al., 2018). The protocol for this review was registered with PROSPERO (CRD42022324200) and Campbell review (ACG‐22‐02), published in September 2023 (Mohd Mydin et al., 2023). We used Preferred Reporting Items for Systematic Reviews and Meta‐Analyses (PRISMA) guidelines to report the screening and selection process, as shown in Figure 1 (Moher, 2009).

### 5.1 Criteria for considering studies for this review

#### 5.1.1 Types of studies

Studies eligible for this review must at least (1) contain information on instrument development, (2) examine content validity, or (3) evaluate one psychometric property of AOP measurement instruments in the community or institutional settings. We also included studies in which AOP measurement instruments are used in a validation study of another instrument.

We excluded studies that used AOP measurement instruments solely for screening purposes to establish a diagnosis in clinical or hospital settings (such as emergency departments) or cases from adult protective services. However, we included studies evaluating screening instruments and determining if they have been utilised in prevalence studies, particularly in community and institutional settings.

#### 5.1.2 Types of participants

The population of interest for this review includes males and females aged 60 or older living in community or institutional settings (i.e., nursing homes, long‐term care facilities, assisted living facilities, residential care institutions, residential facilities, and skilled nursing facilities) in all countries.

#### 5.1.3 Phenomena of interest

Instrument development, content validity and psychometric properties of AOP measurement instruments in the community or institutional settings.

#### 5.1.4 Types of outcome measures

The psychometric properties outlined by the COSMIN Taxonomy for quality of measurement outcome domains will be adhered to (Prinsen et al., 2018). These include three quality domains: reliability, validity, and responsiveness.

We systematically compiled and evaluated all available AOP measurement instruments, ensuring that each instrument had reported on aspects such as instrument development, content validity, evaluation of one or more psychometric properties, AOP definition, and the abuse subtypes covered by the instrument.

##### Primary outcomes

We reported all evaluated psychometric properties of the instruments for AOP prevalence measurement instruments in the community or institutional settings. These reported psychometric properties align with the COSMIN criteria:

1. Instrument development
2. Content validity
3. Structural validity
4. Internal consistency
5. Cross‐cultural validity\measurement invariance
6. Reliability
7. Measurement error
8. Criterion validity
9. Hypotheses testing for construct validity
10. Responsiveness.

The COSMIN methodology for assessing the content validity of measurement instruments focuses on three main aspects: comprehensiveness, relevance, and comprehensibility (Terwee et al., 2018). Comprehensiveness refers to covering all relevant aspects of the construct being measured. Relevance evaluates whether the questions are appropriate and meaningful for the target population and context. Comprehensibility checks whether the language, wording, and formatting of the questions are clear and easily understandable by the target population. Various methods can be used to evaluate each factor, such as expert review, literature review, input from target population members, feedback through focus groups, cognitive interviews, or surveys.

The definitions of the psychometric properties we used are similar to the description provided in the COSMIN methodology for systematic reviews of the Patient‐Reported Outcome Measures (PROMs) manual, as outlined in Supporting Information: Appendix 2 (Mokkink et al., 2018; Prinsen et al., 2018; Terwee et al., 2018).

##### Secondary outcomes

The definitions of AOP and its subtypes for the included AOP measurement instruments are also reported in this review.

#### 5.1.5 Types of settings

The included studies were conducted worldwide in various community or institutional settings (e.g., nursing homes, long‐term care facilities, assisted living facilities, residential care institutions, and skilled nursing facilities).

### 5.2 Search methods for identification of studies

An information specialist designed the primary search strategy that consists of a combination of search terms using the medical subject heading (MeSH) and free text terms that consisted of ‘elder abuse’, ‘elder mistreatment’, ‘elder maltreatment’, ‘elder neglect’ AND ‘psychometric’ OR ‘outcome assessment’ OR reproducible OR reliability OR validity OR ‘screening tool’ OR ‘screening assessment’ OR assessment OR ‘assessment tool’ OR screening OR ‘appraisal tool’. The search strategy was developed, revised by content experts, and piloted in several rounds to improve its sensitivity and specificity. The final strategy was completed in PubMed and replicated in other databases. The final search strategy for all the databases is available in Supporting Information: Appendix 3.

We searched the following sources of information: electronic databases, trial registries, and grey literature. We performed a search of the title, abstract, and keywords through AgeLine via EBSCOhost, ASSIA via ProQuest, CINAHL via EBSCOhost, EMBASE, LILACS, ProQuest Dissertation & Theses Global, PsycINFO via EBSCOhost, PubMed, SciELO, Scopus, Sociological Abstract via ProQuest, Chinese National Knowledge Infrastructure (CNKI), Google Scholar, and WHO Global Index Medicus**.** Letters, editorials, and conference abstracts were excluded.

#### 5.2.1 Searching other resources

We also identified other relevant studies by searching the grey literature from several resources, such as Campbell Collaboration (https://www.campbellcollaboration.org/), OpenAIRE (https://explore.openaire.eu/), and GRAFT (https://www.jurn.link/graft/). Additionally, we conducted direct searches of the entire journal content in prominent academic journals that publish empirical elder abuse research, including the Journal of Elder Abuse and Neglect, Journal of Interpersonal Violence, Journal of Adult Protection, and Journal of Trauma, Abuse, and Violence.

Potential studies that may not have been identified through the searches were identified by searching the references of related studies or reviews. Forward citation searches were used to identify cited studies included in these related studies or reviews.

### 5.3 Data collection and analysis

#### 5.3.1 Description of methods used in primary research

Quantitative, qualitative (addressing face and content validity) and mixed‐method empirical studies were included.

#### 5.3.2 Selection of studies

EndNote and EPPI Reviewer web applications were utilised to manage all documents retrieved throughout the search process. All database citations were imported into the EPPI Reviewer web application (Thomas et al., 2010). Duplicate studies were removed, and titles, abstracts, and full‐text screening were assessed in EndNote.

Two reviewers independently performed the primary screening of studies based on the titles and abstracts. The selected studies were categorised into relevant, irrelevant and unsure groups. Studies deemed irrelevant by both reviewers were excluded from the review. Next, two reviewers independently reviewed and selected the full text of selected studies based on the inclusion and exclusion criteria. Studies that did not meet the inclusion criteria were excluded from the review, and reasons for exclusion were provided in Figure 1. Any disagreements were resolved during the selection and review process through discussion with a third reviewer.

#### 5.3.3 Data extraction and management

Data extraction from the selected full text was conducted and entered into the data extraction form independently by two reviewers to reduce bias and errors. Studies other than those in the English language were translated into English using Google Translate or an expert in the language. A third reviewer checked for any differences between the data extracted by the two reviewers. Any potential differences among the two reviewers were discussed with the team.

Information and the details of the non‐psychometric properties and practical administration of the measuring instruments included in this review were extracted and described for each measurement instrument. We extracted the data on the characteristics of the included measurement instruments, such as the name of the measurement instruments, original or modified, study country, study population, original language, available translations in other languages, constructs being measured, scales or subscales measured, number of items per subscale, the perpetrator (a trusted person who carries out a harmful, illegal, or immoral act to the older adults), response options, recall period, severity and the details of instruments administration such as mode of administration, time to complete the instrument and the definitions used as shown in Table 2.

**Table 2:** haracteristics of the included measuring instruments according to the study setting.

No	Instrument	Reference	Original/modified	Country	Study population	Original language	Available translation	Constructs/Subscales and no. of item for each subscale	No. of items	Response options	Recall period	Mode of administration	Definition	Duration of administration	Severity	Frequency	Perpetrator
COMMUNITY																	
1.	CASE	Reis and Nahmiash (1995)	Original	Canada	C	English	NA	Physical, Psychosocial, Financial, Neglect	8	Yes/No	NA	Interview‐based	Abuse of older people defined as at least four categories: physical, psychosocial and financial/material abuse, and neglect, though abuse is not always categorically or uncontestably definable. Abuse perpetrators are often people in positions of trust, such as caregivers.	1 to 2 min	No	No	No
		Paixão et al. (2007)	Original	Brazil	C	English	Portuguese	Physical, Psychosocial, Financial, Neglect	8	Yes/No	NA	Interview‐based	NA	NA	No	No	No
		Reichenheim et al. (2009)	Original	Brazil	C	English	NA	Physical, Psychosocial, Financial, Neglect	8	Yes/No	NA	Interview‐based	NA	NA	No	No	No
		冯瑞新 (2010)	Original	China	C	English	NA	Physical, Psychosocial, Financial, Neglect	8	Yes/No	NA	Interview‐based	NA	NA	No	No	No
		Pérez‐Rojo et al. (2015)	Original	Spain	C	English	NA	Physical, Psychosocial, Financial, Neglect	8	Yes/No	NA	Interview‐based	Elder abuse has been defined as “a single or repeated act, or lack of appropriate action, occurring within any relationship where there is an expectation of trust, which causes harm or distress to an older person.	NA	No	No	No
		Melchiorre et al. (2017)	Original	Italy	C	English	NA	Physical, Psychosocial, Financial, Neglect	8	Yes/No	NA	Interview‐based	Elder abuse has been defined as “a single or repeated act, or lack of appropriate action, occurring within any relationship where there is an expectation of trust, which causes harm or distress to an older person.	1 to 2 min	No	No	No
		Rivera‐Navarro (2018)	Original	Spain	C	English	NA	Physical, Psychosocial, Financial, Neglect	8	Yes/No	NA	Interview‐based	Family maltreatment in older adults is understood as any act committed by a family member that by action or omission causes physical or psychological harm to an older adult.	45 min	No	No	No
		Sakar et al. (2019)	Original	Iran	C	English	Persian	Physical, Psychosocial, Financial, Neglect	8	Yes/No	NA	Interview‐based	NA	NA	No	No	No
		Khan et al. (2020)	Original	India	C	English	Urdu	Physical, Psychosocial, Financial, Neglect	8	Likert‐scale	NA	Interview‐based	Many forms of elder abuse are recognized as types of domestic or family violence since they are committed by family members. Abusive behaviours take various forms. In the literature, elder abuse has often been categorized as psychological, emotional, physical, or sexual abuse with adverse consequences in elders’ lives.	NA	No	No	No
2.	VASS‐12 items	Schofield (2002)	Original	Australia	O	English	NA	3 Vulnerability, 3 Dependence, 3 Dejection, 3 Coercion	12	Yes/No	12 months	Self‐report, interview‐based	Four key forms of abuse against older people: physical abuse (e.g., striking, restraining), psychosocial abuse (e.g., insulting, isolating), material/financial abuse (e.g., theft, misuse of property), and neglect (e.g., not providing medical care, lack of supervision), whether intentional or not.	NA	No	No	No
		Schofield and Mishra (2003)	Original	Australia	O	English	NA	3 Vulnerability, 3 Dependence, 3 Dejection, 3 Coercion	12	Yes/No	12 months	Self‐report	NA	NA	No	No	No
		Buri et al. (2009)	Original	USA	O	English	NA	3 Vulnerability, 3 Dependence, 3 Dejection, 3 Coercion	12	Yes/No	12 months	Self‐report	NA	NA	No	No	No
		Dong and Simon (2013)	Original	China	O	English	Mandarin	3 Vulnerability, 3 Dependence, 3 Dejection, 3 Coercion	12	Yes/No	12 months	Self‐report	NA	NA	No	No	No
		Maia (2014)	Original	Brazil	O	English	Portuguese	3 Vulnerability, 3 Dependence, 3 Dejection, 3 Coercion	12	Yes/No	12 months	Self‐report	NA	NA	No	No	No
		Maia (2016)	Original	Brazil	O	English	Portuguese	3 Vulnerability, 3 Dependence, 3 Dejection, 3 Coercion	12	Yes/No	12 months	Interview‐based	NA	NA	No	No	No
		Asiret (2017)	Original	Brazil	O	English	Turkish	3 Vulnerability, 3 Dependence, 3 Dejection, 3 Coercion	12	Yes/No	12 months	Interview‐based	Elder abuse has been defined as “a single, or repeated act, or lack of appropriate action, occurring within any relationship where there is an expectation of trust which causes harm or distress to an older person”	20 min	No	No	No
		Dantas et al. (2017)	Original	Iran	O	English	Turkish	3 Vulnerability, 3 Dependence, 3 Dejection, 3 Coercion	12	Yes/No	12 months	Interview‐based	NA	2 min	No	No	No
		Motahedi et al. (2021)	Original	USA	O	English	Persian	3 Vulnerability, 3 Dependence, 3 Dejection, 3 Coercion	12	Yes/No	12 months	Interview‐based	Elder abuse involves as actions that cause harm or create a serious risk of harm to a vulnerable elder by a caregiver or other person who stands in a trust relationship to the elder.	5 min	No	No	No
3.	HS‐EAST	Hwalek and Sengstock (1986)	Original	USA	O, S	English	NA	1 Physical (direct abuse), 4 Violation of personal rights, 3 Characteristic of vulnerability, 7 Potentially abusive situation	15	Yes/No	12 months	Self‐report, interview‐based	(1) Physical Abuse. Direct attacks against a person such as a hit, as well as threats in which a weapon is involved. (2) Physical Neglect. Failure to provide an punch or aged and dependent person with the necessities of life such as food, clothing, or shelter. (3) Psychological Abuse. The measurement of verbal assaults such as screaming or ridicule, and threats that induce fear but do not make use of a weapon. (4) Psychological Neglect. Failure of the caretaker to satisfy the emotional or psychological needs of the aged under their care‐for example, isolating the elderly person or not providing any social or cognitive stimulation. (5) Material Abuse. Stealing or misusing money orto the elderly person. (6) Violation of Personal Rights. Depriving an right to freedom of choice, life, or privacy. (7) Risk Indicators. Items that did not directly assess symptoms identifying abuse and neglect but were included on the indexes because they were felt to be predictive of the presence of abuse and/or neglect.	NA	No	No	No
		Neale (1991)	Original	USA	O, S	English	NA	1 Physical (direct abuse), 4 Violation of personal rights, 3 Characteristic of vulnerability, 7 Potentially abusive situation	15	Yes/No	12 months	Interview‐based	NA	NA	No	No	No
		Moody (2000)	Original	USA	O, S	English	NA	1 Physical (direct abuse), 4 Violation of personal rights, 3 Characteristic of vulnerability, 7 Potentially abusive situation	15	Yes/No	12 months	Self‐report, interview‐based	NA	5 to 10 min	No	No	No
		Reichenheim et al. (2008)	Original	Brazil	O, S	English	Portuguese	1 Physical (direct abuse), 4 Violation of personal rights, 3 Characteristic of vulnerability, 7 Potentially abusive situation	15	Yes/No	12 months	Interview‐based	NA	NA	No	No	No
		Buri et al. (2009)	Original	USA	O, S	English	NA	1 Physical (direct abuse), 4 Violation of personal rights, 3 Characteristic of vulnerability, 7 Potentially abusive situation	15	Yes/No	12 months	Self‐report, interview‐based	NA	NA	No	No	No
		Jervis et al. (2014)	Original	Iran	O, S	English	NA	1 Physical (direct abuse), 4 Violation of personal rights, 3 Characteristic of vulnerability, 7 Potentially abusive situation	15	Yes/No	12 months	Interview‐based	NA	NA	No	No	No
		Hosseinkhani et al. (2017)	Original	Türkiye	O, S	English	Persian	1 Physical (direct abuse), 4 Violation of personal rights, 3 Characteristic of vulnerability, 7 Potentially abusive situation	15	Yes/No	12 months	Interview‐based	Elder abuse includes physical, emotional, sexual abuse, ignorance, abandon, and misuse of elders. It refers to a group of behaviours that hurt the elders or cause them serious problems and are performed by persons the elders trust. In addition, ignoring elders' care by not providing their essential needs and not protecting them from being hurt are included in this definition.	NA	No	No	No
		Özçakar et al. (2017)	Original	Türkiye	O, S	English	Turkish	1 Physical (direct abuse), 4 Violation of personal rights, 3 Characteristic of vulnerability, 7 Potentially abusive situation	15	Yes/No	12 months	Interview‐based	NA	NA	No	No	No
		Ozmete (2017)	Original	Türkiye	O, S	English	Turkish	1 Physical (direct abuse), 4 Violation of personal rights, 3 Characteristic of vulnerability, 7 Potentially abusive situation	15	Yes/No	12 months	Interview‐based	Elder abuse as ‘‘a single or repeated act, or lack of appropriate action, occurring within a relationship where there is an expectation of trust which causes harm or distress to an older person’’	NA	No	No	No
		Akyol Guner (2022)	Original	Uganda	O, S	English	Turkish	1 Physical (direct abuse) 4 Violation of personal rights, 3 Characteristic of vulnerability, 7 Potentially abusive situation	15	Yes/No	12 months	Interview‐based	Elder abuse as ‘‘a single or repeated act, or lack of appropriate action, occurring within a relationship where there is an expectation of trust which causes harm or distress to an older person’’	15 to 20 min		No	No
		Atim et al. (2023)	Original	USA	O, S	English	Uganda local language	1 Physical (direct abuse), 4 Violation of personal rights, 3 Characteristic of vulnerability, 7 Potentially abusive situation	15	Yes/No	12 months	Interview‐based	Elder abuse is defined as ‘a single, or repeated act, or lack of appropriate action, occurring within any relationship where there is an expectation of trust, which causes harm or distress to an older person’	40 min	Yes	No	No
4.	mGMS (Neglect)	Zawisza et al. (2020)	Original	Poland	O	English	Polish	12 Neglect (8 Basic needs, 4 Psychological needs)	12	Likert‐scale	12 months	Self‐report, interview‐based	There are a variety of definitions of neglect of older people. Based on The World Health Organization definition of older abuse, neglect was operationalized as “the refusal or failure to fulfil a caregiving obligation” or “the refusal or failure of responsible caregivers to provide a care dependent older adult with assistance in daily living tasks or essential support such as food, clothing, shelter, health and medical care”, neglect is “the failure to provide adequate medical or personal care/services necessary to maintain the physical and mental health of a vulnerable older person by a designated caregiver.” A more general definition has been proposed “intentional or unintentional withholding of food, medication or other necessities that result in the older person's failure to thrive.”	NA	No	Yes	Yes
5.	GMS	Giraldo‐Rodríguez (2013)	Original	Mexico	O	Spanish	English	5 Physical, 6 Psychological, 5 Economic, 2 Sexual, 4 Neglect	22	Frequency count	12 months	Interview‐based	NA	NA	No	Yes	Yes
		Giraldo‐Rodríguez (2015)	Original	Mexico	O	English	NA	5 Physical, 6 Psychological, 5 Economic, 2 Sexual, 4 Neglect	22	Yes/No	NA	Self‐report	Elder abuse has been defined as intentional actions that cause harm or create a serious risk of harm (whether or not harm is intended) to a vulnerable older person by a caregiver or another person who has a trust relationship with the older person. Many forms of elder abuse exist, including physical, sexual, and psychological abuse, as well as financial exploitation and neglect.	NA	No	Yes	Yes
		Dasbas (2019)	Original	Turkiye	O	English	Turkish	5 Physical, 6 Psychological, 5 Economic, 2 Sexual, 4 Neglect	22	Yes/No	1 year	Interview‐based	Elder abuse can be defined as a single or repeated act or lack of appropriate action occurring within any relationship where there is an expectation of trust, which causes harm or distress to an older person.	NA	No	Yes	Yes
		Pabon‐Poches (2019)	Original	Columbia	O	Spanish	English	5 Physical, 6 Psychological, 5 Economic, 2 Sexual, 4 Neglect	22	Yes/No	NA	Interview‐based	Abuse is understood as the action that causes physical or psychological damage to an elderly person in a unique or repeated way and/or due to lack of timely action, which is generated within any relationship where there is an expectation of trust and of which five types can be distinguished: physical abuse, psychological or emotional, economic or material exploitation, neglect and sexual abuse.	8 to 20 min	No	Yes	Yes
		Rashidi Fakari (2020)	Original	Tehran	O	Persian	NA	5 Physical, 6 Psychological, 5 Economic, 2 Sexual, 4 Neglect	22	Yes/No	NA	Self‐report	NA	NA	No	Yes	Yes
6.	mCTS‐M 38 items	Wazid et al. (2021)	Modified	Malaysia	O	English	Malay	8 Physical, 7 Psychological, 3 Sexual, 9 Financial, 11 Neglect	38	Yes/No	12 months	Self‐report	Elder abuse and neglect is defined as “a single or repeated act, or lack of appropriate action, occurring within any relationship where there is an expectation of trust that causes harm or distress to an older person”. Classification of EAN is either based on the five common subtypes – physical abuse, psychological abuse, financial abuse, sexual abuse, and neglect – or derived from the setting in which mistreatment occurs, domestic or institutional.	NA	Yes	Yes	Yes
		Sooryanarayana (2017)	Modified	Malaysia	O	English	NA	8 Physical, 7 Psychological, 3 Sexual, 9 Financial, 11 Neglect	38	Yes/No	12 months	Interview‐based	Elder abuse is defined as “a single or repeated act, or lack of appropriate action, occurring within any relationship where there is an expectation of trust that causes harm or distress to an older person”.	NA	No	No	No
7.	DEAQ	Heravi‐Karimooi (2010)	Original	Iran	O	Persian	NA	6 Physical, 8 Psychological, 2 Emotional neglect, 4 Financial, 4 Sexual, 11 Neglect, 4 Rejection, 10 Denial of choice	49	Yes/No	NA	Interview‐based	NA	20 min	No	No	No
		Keyghobadi et al. (2014)	Original	Iran	O	Persian	NA	6 Physical, 8 Psychological, 2 Emotional neglect, 4 Financial, 4 Sexual, 11 Neglect, 4 Rejection, 10 Denial of choice	49	Yes/No	NA	Interview‐based	Older person abuse is an intentional act or failure to act that causes or creates a risk of harm to an older adult. An older adult is someone aged 60 or older.	NA	No	No	No
		Morowatisharifabad et al. (2016)	Original	Iran	O	English	Persian	6 Physical, 8 Psychological, 2 Emotional neglect, 4 Financial, 4 Sexual, 11 Neglect, 4 Rejection, 10 Denial of choice	49	Yes/No	NA	Interview‐based	Older person abuse is an intentional act or failure to act that causes or creates a risk of harm to an older adult. An older adult is someone aged 60 or older.	20 to 30 min	No	No	No
8.	FIVE	Hancock (2023)	Original	USA	O	English	NA	5 Financial	5	Yes/No	NA	NA	Financial abuse of older adults occurs when a person in a position of trust obtains property or assets through deception or intimidation, or improperly uses the assets of an older adult.	NA	Yes	Yes	No
9.	MEAS	Hamid Tengku et al. (2013)	Original	Malaysia	O	English	Malay	4 physical, 4 psychological, 2 financial	10	Yes/No	NA	Interview‐based	NA	NA	No	No	No
10.	*NAS (Ghana)	Asiamah et al. (2021)	Original	Ghana	O	English	NA	Neglect and Abuse, Discrimination and Exploitation	11	Likert‐scale	NA	Self‐report	NA	NA	No	Yes	No
11.	EACS	Neise et al. (2022)	Original	Germany	O	German	NA	Intimidation, Shaming and blaming, Paternalism, Neglect, Financial exploitation, Physically abusive behaviour, Unwanted custodial measures, Sexualised	16	Likert‐scale	12 months	Computer‐assisted interview	Elder abuse is defined as “a single or repeated act, or lack of appropriate action, occurring within any relationship where there is an expectation of trust that causes harm or distress to an older person”.	90 min	No	Yes	No
12.	Native EAS‐Short Form	Ghahar,i 2018	Original	Iran	O	English	NA	6 Emotional, 10 Verbal, 3 Financial, 4 Neglect	23	NA	NA	Self‐report, interview‐based	Elder abuse as “a single, or repeated act, or lack of appropriate action, occurring within any relationship where there is an expectation of trust, which causes harm or distress to an older person”. Elder abuse can be seen in various forms including physical, psychological or emotional, sexual, financial abuse, intentional or unintentional neglect of that, emotional abuse has been reported as the most common one.	NA	No	No	No
13.	FVOW	Paranjape et al. (2009)	Original	USA	O	English	NA	7 Physical, 14 Emotional, 2 Financial, 1 Sexual, 4 Neglect, 5 Coercion	33	Frequency count	NA	Interview‐based	Family violence broadly, citing examples of abuse (physical, sexual, emotional and financial) and neglect.	NA	No	Yes	No
14.	mVASS‐15 items	徐金燕 and 蒋利平 (2020)	Modified	China	O	Mandarin	English	6 Physical, 4 Emotional, 3 Economic, 2 Neglect	15	Yes/No	12 months	Interview‐based	NA	NA	No	Yes	No
15.	FVS	Préville et al. (2014)	Original	Canada	O	English	French	21 items (Spousal: 3 Physical, 4 Psychological, 4 Financial, 3; Children: 2 Physical, 4 Psychological, 4 Financial)	21	Yes/No	12 months	Interview‐based	NA	90 min	No	No	No
16.	*IPPA (India)	Bajpai (2023)	Original	India	O, S, C	Tamil	NA	8 Physical, 11 Psychological, and 5 Elder Abuse	24	Likert‐scale	NA	Interview‐based	(2003) The psychological abuse is one of the types of abusive behaviour affecting elder people. Several researchers attempted to define psychological abuse as the destructive action, mental torture or derailment of mind or suffering from an emotional situation such as harassment, threats, humiliation, or intimidation, attack on self‐esteem or sense of safety of the person by another. Psychological abuse is the deliberate infliction of the mental or emotional suffering of humiliation, threat, or other verbal/nonverbal conduct Under psychological abuse due to the verbal and non‐verbal acts person feels mental pain, anguish, stress, headache or agony.	40 to 45 min	No	No	No
17.	IEAQ‐Long form	Ghahari et al. (2018)	Original	Iran	O	English	NA	8 Emotional, 4 Financial, 4 Neglect, 1 Sexual, 4 Ignoring needs and demands, 3 Compulsion, 2 Secrecy, 2 Mental pressure, 2 Mistreatment, 4 Insulting, 2 Deprivation, 4 Imposition, 2 Domination	42	Likert‐scale	NA	Self‐report, interview‐based	Elder abuse is defined as “a single or repeated act, or lack of appropriate action, occurring within any relationship where there is an expectation of trust that causes harm or distress to an older person”.	1 to 2 h	No	Yes	No
18.	EVEQ	Ajdikovic (2008)	Original	Croatia	O	English	NA	9 Physical, 11 Psychological, 5 Material, 3 Sexual	28	Frequency count	NA	Interview‐based	Elder abuse is defined as “a single or repeated act, or lack of appropriate action, occurring within any relationship where there is an expectation of trust that causes harm or distress to an older person”.	NA	No	Yes	Yes
19.	EMM	Wong et al. (2021)	Original	USA	O	English	NA	2 Physical, 5 Emotional, 2 Financial	10	Yes/No	Since turning 60 years old	Interview‐based	Elder mistreatment is a public health issue defined as “an intentional act, or failure to act, by a caregiver or another person in a relationship involving an expectation of trust that causes or creates a risk of harm to an older adult”	NA	Yes	No	Yes
20.	*EAN (Puerto Ricans)	Irizarry‐Irizarry (2008)	Original	Puerto Rico	O	English	Spanish	Exposure to abuse (items for subscale not mentioned)	23	Yes/No/Neutral	NA	Interview‐based	NA	NA	No	No	No
21.	EMS	Wu (2012)	Original	China	O	English	Chinese	Physical, Psychological, and Financial Neglect	NA	Yes/No	12 months	Interview‐based	Elder mistreatment as ‘‘intentional actions that cause harm or create a serious risk of harm, whether or not intended, to a vulnerable elder by a caregiver or other person who stands in a trust relationship to the elder or failure by a caregiver to satisfy the elder's basic needs or to protect the elder from harm’’	NA	No	No	No
22.	WHRS	Fisher and Regan (2006)	Original	USA	O	English	NA	4 physical, 3 Psychological/Emotional, 3 Sexual, 3 Control, 2 Threat	15	Frequency count	Starting 55 years old	Self‐report, interview‐based	Older person abuse is an intentional act or failure to act that causes or creates a risk of harm to an older adult. An older adult is someone aged 60 or older.	20 to 45 min	No	Yes	No
23.	*IPVQ	Charro‐Baena et al. (2023)	Original	Spain	O	Spanish	NA	8 Physical, 6 Psychological, 3 Verbal, 3 Economic, 4 Social	24	Yes/No	Lifetime	Interview‐based	Intimate partner violence (IPV) is any behavior within an intimate relationship that causes physical, psychological, or sexual harm to the partners. It includes controlling behaviors such as isolation from family members and/or friends and restriction of access to employment, education, financial resources, or healthcare.	NA	No	No	No
24.	*mNSEAN (Neglect)	Ayalon (2015)	Modified	Israel	O, C, S	English	NA	7 Neglect	7	NA	NA	Interview‐based	Neglect is defined as the “intentional or unintentional withholding of food, medication or other necessities that result in the older person's failure to thrive”. An alternative definition of neglect puts the responsibility for neglect on the caregiver. The National Research Council defines elder neglect as “an omission by responsible caregivers that constitutes “neglect” under applicable federal or state law”.	NA	No	No	No
25.	mOARS‐ADL(Neglect)	Fang and Yan (2021)	Original	China	O & C	English	NA	3 Neglect	3	NA	NA	Self‐report, interview‐based	NA	NA	No	No	No
26.	mCTS‐C 25 Items	Yan and Tang (2001)	Modified	Hong Kong	O	English	Chinese	12 Physical, 8 Verbal, 5 Social Abuse	25	Yes/No	12 months	Interview‐based	Elder abuse includes both abuse and neglect. Physical abuse refers to being hit, assaulted, burned or physically restrained, verbal abuse involves being insulted, frightened, humiliated, or intimidated. Social abuse involves involuntary isolation of elderly people or forcing them to enter nursing home.	NA	No	No	No
		Yan and Tang (2004)	Modified	Hong Kong	O	English	Chinese	12 Physical, 8 Verbal, 5 Social Abuse	25	Yes/No	12 months	Interview‐based	Elder abuse is broadly defined to include the above types of abusive behaviour committed against elder people.	NA	No	No	No
		Fang and Yan (2021)	Modified	China	O	English	NA	12 Physical, 8 Verbal, 5 Social Abuse	25	Yes/No	12 months	Interview‐based	Elder abuse is defined as “a single or repeated act, or lack of appropriate action, occurring within any relationship where there is an expectation of trust that causes harm or distress to an older person”.	NA	No	Yes	No
27.	mCTS‐10 items	Beach et al. (2005)	Modified	USA	C	English	NA	5 Physical, 5 Psychological	10	Frequency count	3 months	Interview‐based	NA	1.5 to 2 h	No	Yes	No
		Cooper, Manela, et al. (2008)	Modified	UK	C	English	NA	5 Physical, 5 Psychological	10	Frequency count	3 months	Interview‐based	NA	NA	No	Yes	No
		Cooper et al. (2009)	Modified	UK	C	English	NA	5 Physical, 5 Psychological	10	Frequency count	3 months	Self‐reported and interview‐based	Elder abuse is a violation of a vulnerable older person's human and civil rights by another person or persons.	About 1 h	Yes	Yes	No
		Lafferty (2016)	Modified	Ireland	C	English	NA	5 Physical, 5 Psychological	10	Frequency count	3 months	Self‐report	NA	NA	No	Yes	No
		Qin and Yan (2018)	Modified	NA	C	NA	NA	5 Physical, 5 Psychological	10	Frequency count	3 months	NA	Domestic violence is defined as maltreatment conducted by family members, including physical, psychological, emotional, financial, and sexual abuse, neglect, and abandonment	NA	No	No	No
28.	*mNSEAN	Ayalon (2011)	Modified	Israel	O, C, S	English	NA	2 Physical, 6 Emotional, 5 Financial,2 Sexual, 7 Neglect	22	NA	NA	Self‐report, interview‐based	Elder mistreatment is defined as intentional actions aimed to cause harm or to create a serious risk of harm, or a failure to satisfy the basic needs of a vulnerable older adult by a trusted individual. This definition of elder mistreatment refers to abuse, neglect, abandonment, discrimination against and exploitation of the older adult.	45 min	No	No	Yes
29.	*EANQ (Iran)	Manoochehri et al. (2008)	Original	Iran	O, C, S	Persian	NA	7 Physical, 7 Emotional, 6 Financial, 1 Sexual, 9 Neglect	30	Likert‐scale	NA	Interview‐based	NA	NA	No	No	yes
30.	mCTS‐T 18 items	Chokkanathan (2005)	Modified	India	O	English	Tamil	6 Physical, 8 Verbal, 2 Financial, 2 Neglect	18	Frequency count	12 months	Interview‐based	Elder mistreatment encompasses both abuse and neglect. Elder abuseis a single or repeated act or lack of appropriate action occurring within any relationship where there is an exception of trust, which causes harm or distrust to an older person. The types of abuse as investigated in the past twelve months were: • Chronic verbal abuse included 10 or more instances of insults, swearing, calling names, etc. • Financial abuse was defined as at least one instance of older adults being forced to part with their money or assets; family members utilizing money or assets belonging to the older adults without the latter's knowledge. • Physical abuse included at least one instance of shoving, pushing, beating, slapping, kicking, punching, etc. • Chronic neglect was defined as 10 or more instances of caregivers’ failure to provide older adults with basic needs or to take care of them during illness.	NA	No	Yes	Yes
31.	mCTS‐C 18 items	Yan and Kwok (2011)	Modified	Hong Kong	C	NA	NA	12 Physical, 6 Psychological	18	NA	NA	NA	Elder abuse refers to ‘‘actions that cause harm or create a serious risk of harm, whether or not intended, to a vulnerable elder by a caregiver or other person who stands in a trust relationship to the elder, or failure by a caregiver to satisfy the elder's basic needs or to protect the elder from harm"	NA	No	No	No
32.	mCTS‐C 23 items	Chen and Chan (2022)	Modified	China	C	English	Chinese	12 Physical (5 minor, 7 major), 8 Psychological (4 items minor, 4 major), 2 Neglect, 1 Injury to older people	23	Likert‐scale	12 months	Interview‐based	Older adults can be vulnerable to different forms of abuse, including physical, psychological, sexual, and financial abuse. Neglect is also a form of elder abuse that can be difficult to identify; it refers to the failure of a caregiver to satisfy the basic needs of an elderly person. The perpetrator of elder abuse could be a caregiver or another person with whom the elder person fosters a bond of trust.	NA	Yes	Yes	No
33.	ABUEL	Melchiorre et al. (2013)	Original	Greece	O	English	NA	17 Physical, 11 Psychological, 8 Sexual, 9 Financial, 13 Neglect, 7 Injuries	52	Frequency count	12 months	Self‐report, interview‐based	NA	NA	No	Yes	Yes
		Melchiorre et al. (2016)	Original	Greece	O	English	NA	17 Physical, 11 Psychological, 8 Sexual, 9 Financial, 13 Neglect, 7 Injuries	52	Yes/No	12 months	Self‐report, interview‐based	NA	NA	No	Yes	Yes
34.	EAVQ	Kong and Jeon (2018)	Original	Korea	O	Korean	English	7 Emotional	7	Yes/No	12 months	Interview‐based	NA	NA	No	No	No
35.	USCOACS	DeLiema et al. (2012)	Original	USA	O	English	Spanish	15 Physical, 9 Psychological, 4 Sexual, 14 Financial, 12 Neglect,	54	Yes/No	12 months	Interview‐based	NA	60 to 90 min	Yes	No	No
36.	m‐EAQ	Chang (2016)	Modified	USA	O	English	Korean	3 Physical, 6 Psychological, 5 Financial, 5 Sexual, 3 Neglect	22	Yes/No	NA	Self‐report	Elder abuse defined as harmful or hurtful behaviour that is intentionally inflicted upon an older person.	30 min	No	No	No
37.	mCTS‐Verbal 12 items	Fulmer et al. (2014)	Modified	USA	O	English	NA	6 Verbal	6	Frequency count	12 months	Audio‐assisted computer self‐interview	Elder mistreatment has been defined as “(a) intentional actions that cause harm or create a serious risk of harm (whether or not harm is intended) to a vulnerable elder by a caregiver or other person who stands in a trust relationship to the elder or (b) failure by a caregiver to satisfy the elder's basic needs or to protect the elder from harm.	NA	No	Yes	No
38.	mVASS‐10 items	Dong et al. (2014)	Modified	USA	O	English	NA	(a) family conflicts at home, (b) felt uncomfortable with someone in the family, (c) felt that nobody wanted them around, (d) been told by someone that they gave too much trouble, (e) been afraid of someone in the family, (f) felt that someone close tried to hurt or harm them, (g) been neglected or confined, (h) been called names or put down, (i) been forced by someone to do things, or (j) had belongings taken without permission.	10	Yes/No	NA	Interview‐based	NA	NA	No	No	No
39.	mVASS‐16 items	钱振中 (2016)	Modified	China	O	Mandarin	NA	5 Physical, 6 Mental, 2 Economic Exploitation, 3 Neglect	16	NA	NA	Self‐report	NA	NA	No	No	No
40.	m‐HS‐EAST	Alizadeh‐Khoei et al. (2014)	Modified	Iran	O	English	Farsi	Physical, Psychosocial, Financial, Sexual, Neglect	NA	Yes/No	12 months	Interview‐based	Abuse is described s as ‘‘a violation of an individual'shuman and civil rights by another person’’. Abuse can be categorized as physical, psychological, sexual, financial, or discriminatory abuse or neglect.	NA	No	No	No
41.	ATDEA	Yi (2019)	Original	China	O, S, C	Chinese	NA	4 Physical, 7 Neglect, 6 Psychological, 4 Sexual, 3 Economic, 8 Self‐neglect, 4 Social	36	Likert‐scale	NA	Self‐report	Elder abuse is defined as “a single or repeated act, or lack of appropriate action, occurring within any relationship where there is an expectation of trust that causes harm or distress to an older person”.	NA	No	No	No
		Yang et al. (2022)	Original	China	O, S, C	Chinese	NA	Physical, Psychological, Neglect, Economic, Social	24	Likert‐scale	NA	Interview‐based	NA	NA	Yes	No	No
42.	*EA (Korea)	Oh et al. (2006)	Original	Korea	O	English	NA	5 physical, 5 emotional, 5 economic, 5 verbal, 5 neglect	25	Yes/No	1 month	Interview‐based	In this broad definition, abuse refers to an act of commission or omission, resulting in intentional or unintentional harm or injury, and in one or more types. The Department of Health guideline on policies and procedures to protect vulnerable adults from abuse, sets out a definition of abuse as ‘‘a violation of an individual's human and civil rights by any other person or persons’’. Thus, elder abuse is not only consisting of active physical abuse of an older person but it also includes, in its most common and less dramatic form, exploitation, neglect, and psychological mistreatment.	NA	No	Yes	No
43.	*DVE (Thailand)	Panjaphothiwat (2021)	Original	Thailand	O	English	NA	17 items DV among older people	17	Yes/No	NA	Self‐report, interview‐based	Domestic violence is defined as any form of violence or abuse that occurs in any relationship within a family.	30 min	No	No	No
44.	*EAQ (Nepal)	Yadav et al. (2018)	Original	Nepal	O	English	NA	Physical, Psychological, Financial, Sexual, Neglect	21	Yes/No	3 months	Interview‐based	NA	NA	No	No	No
45.	*EAQ (Iran)	Honarvar et al. (2020)	Original	Iran	O	NA	NA	4 Physical, 9 Emotional, 4 Financial, 4 Sexual, 9 Neglect, 4 Violation of Personal rights	34	Likert‐scale	NA	Interview‐based	Elder abuse is defined as “a single or repeated act, or lack of appropriate action, occurring within any relationship where there is an expectation of trust that causes harm or distress to an older person”.	NA	Yes	Yes	No
46.	AAT	Sembiah et al. (2020)	Original	India	O	Bengali	NA	6 Physical, 7 Psychological, 4 Financial, 4 Neglect	21	Yes/No	12 months	Interview‐based	Elder abuse is defined as “a single or repeated act, or lack of appropriate action, occurring within any relationship where there is an expectation of trust that causes harm or distress to an older person”.	NA	No	Yes	Yes
47.	m‐EASI	Badenes‐Ribera et al. (2021)	Modified	Italy	O	English	NA	1 Physical/Sexual, 1 Emotional/Psychological/Verbal, 1 Financial, 1 Neglect, 1 Dependency	5	Yes/No	NA	Self‐report	Elder mistreatment, defined as intentional harm inflicted on an elder or failure to protect an elder from harm or meet an elder's basic needs, is a serious human rights violation.	NA	No	No	No
48.	QEEA	Badenes‐Ribera et al. (2021)	Original	Italy	O	English	NA	4 Physical/Sexual, 4 Emotional/Psychological/Verbal, 2 financial, 2 Neglect	12	Yes/No	NA	Self‐report	Elder mistreatment, defined as intentional harm inflicted on an elder or failure to protect an elder from harm or meet an elder's basic needs, is a serious human rights violation.	NA	No	No	Yes
49.	*EAQ (China)	Su et al. (2011)	Original	China	O	Chinese	NA	Physical (common and severe), Emotional, Financial, Neglect	21	Yes/No	NA	Self‐report	NA	NA	No	No	No
50.	EAS	Karimi and Elahi (2008)	Original	Iran	O	English	NA	The number of items and subscales not mentioned	NA	NA	Unclear	Interview‐based	NA	45 min	No	No	No
51.	FPS‐2 items	Chan et al. (2019)	Original	China	O & C	English	NA	2 EAN	2	Yes/No	12 months	Self‐report, interview‐based	Family polyvictimization is defined as the co‐occurrence of child abuse and neglect, parental intimate partner violence, and elder abuse against different members in the same family.	NA	No	No	Yes
52.	mCTS‐34 items	Lang et al. (2014)	Modified	5 European countries	O	English	NA	4 Physical, 9 Emotional,4 Financial, 4 Sexual, 9 Neglect, 4 Violation of personal rights	34	Frequency count	12 months	Self‐report, interview‐based	Elder abuse is defined as “a single or repeated act, or lack of appropriate action, occurring within any relationship where there is an expectation of trust that causes harm or distress to an older person”.	NA	Yes	Yes	No
		Luoma (2011)	Modified	Portugal	O	English	NA	4 Physical, 9 Emotional,4 Financial, 4 Sexual, 9 Neglect, 4 Violation of personal rights	34	Yes/No	12 months	Self‐report, interview‐based	Elder abuse is “a single or repeated act, or lack of appropriate action, occurring within any relationship where there is an expectation of trust, which causes harm or distress to an older person.	20 min	Yes	Yes	Yes
53.	mFPS‐5 items	Kita et al. (2023)	Modified	Japan	O & C	English	NA	5 EAN	5	Yes/No	NA	Self‐report, interview‐based	The co‐occurrences and multiple experiences of Interpersonal Violence, Child Abuse and Neglect, and/or Elder Abuse in households have been often observed in clinical settings and reported by recent studies. Such a phenomenon, defined as “family polyvictimization”.	NA	No	Yes	Yes
54.	*mRAAL‐27 items	Steinsheim et al. (2022)	Modified	Norway	C	English	Norwegian	9 Physical, 5 Psychological, 3 financial, 3 Sexual, 7 neglect, 1 depriving the person with dementia of assistive devices	27	Likert‐scale	3 months	Interview‐based	Elder abuse is defined as “a single or repeated act, or lack of appropriate action, occurring within any relationship where there is an expectation of trust that causes harm or distress to an older person”.	NA	No	No	Yes
55.	UKNPS	McCreadie et al. (2006); O'Keeffe (2007)	Original	Northern Ireland	O	English	NA	The number of items not mentioned (Physical, Psychological, Financial, Sexual, Neglect)	NA	NA	12 months	Self‐report, interview‐based	Elder abuse is defined as “a single or repeated act, or lack of appropriate action, occurring within any relationship where there is an expectation of trust that causes harm or distress to an older person”.	NA	Yes	Yes	Yes
56.	OAPAM	Conrad et al. (2009)	Original	USA	O	English	NA	31 Psychological	31	Yes/No	12 months	Interview‐based	Psychological abuse, i.e., a verbal or nonverbal act that inflicts emotional pain, anguish, or distress on the elder, which can range from a simple verbal insult to an extreme form of verbal punishment. Examples include ignoring the elder, habitual scapegoating or blaming, name‐calling, threatening to punish or deprive, attacks against property, intimidation, treating an elder like an infant, and yelling or screaming.	50 min	Yes	Yes	Yes
		Conrad et al. (2011)	Original	USA	O	NA	NA	NA	56	Yes/No	12 months	NA	Psychological abuse, i.e., a verbal or nonverbal act that inflicts emotional pain, anguish, or distress on the elder, which can range from a simple verbal insult to an extreme form of verbal punishment. Examples include ignoring the elder, habitual scapegoating or blaming, name‐calling, threatening to punish or deprive, attacks against property, intimidation, treating an elder like an infant, and yelling or screaming.	60 to 90 min	Yes	No	No
		Alhalal et al. (2022)	Original	UAE	S	Arabic	NA	31 Psychological	30	Yes/No	12 months	Interview‐based	Elder abuse is defined as “a single or repeated act, or lack of appropriate action, occurring within any relationship where there is an expectation of trust that causes harm or distress to an older person”.	NA	Yes	No	No
INSTITUTION																	
57.	R‐REM	Ramirez et al. (2013)	Original	USA	S	English	NA	4 Physical, 7 Verbal	11	Frequency count	2 weeks	Interview‐based	Intentional actions that cause harm or create a serious risk of harm (whether or not harm is intended) to a vulnerable elder by a caregiver or other person who stands in a trust relationship to the elder, or failure by a caregiver to satisfy the elder's basic needs or protect the elder from harm.	NA	No	No	No
		Teresi et al. (2014)	Original	USA	O	English	NA	4 Physical, 7 Verbal	11	Frequency count	2 weeks	Interview‐based	Resident‐to‐resident mistreatment is defined as negative and aggressive physical, sexual, or verbal interactions between residents in long‐term care settings, which in a community setting would likely be considered as unwelcome.	45 min	Yes	Yes	Yes
58.	*EAS (Korea)	Kim et al. (2006)	Original	Korea	S	Korean	NA	Physical I‐threat to safety, Physical II‐direct bodily injury, Verbal and Emotional, Financial abuse, Neglect, Self‐neglect	33	Likert‐scale	NA	Self‐report	NA	NA	No	Yes	Yes
		Lee (2008)	Original	Korea	C	English	NA	Physical I‐threat to safety, Physical II‐direct bodily injury, Verbal and Emotional, Financial abuse, Neglect, Self‐neglect	6	Likert‐scale	NA	Interview‐based	NA	NA	Yes	Yes	No
59.	*ICNH (Norwegian)	Malmedal et al. (2009)	Original	Norway	S	English	NA	5 Physical, 7 Emotional, 1 Financial, 7 Neglect	20	Yes/No	NA	Interview‐based	Inadequate care is used as an overarching concept including abuse and neglect, irrespectively whether the act is intentional or unintentional.	NA	No	Yes	No
		Malmedal (2014)	Original	Norway	S	English	NA	3 Physical, 4 Emotional, 8 Neglect	15	Yes/No	NA	Interview‐based	In the present study, the term inadequate care will be used as the all‐inclusive term; implicit in the term are abuse and neglect. The term inadequate is used when they claim that: “inappropriate and inadequate behaviour can be manifested in a number of ways, which are labelled ‘physical abuse,’ ‘psychological abuse,’ ‘physical neglect’ and so forth”. Inadequate care therefore encompasses a variety of actions or lack of actions that can beharmful to residents of nursing homes.	NA	No	Yes	No
60.	CPEAB	Wang (2005)	Original	Taiwan	C & S	English	NA	20 Psychological	20	Likert‐scale	NA	Interview‐based	Psychological abuse of elders refers to both intentional and unintentional cases and was defined as follows: failure to respect an elderly person as a human being; failure to provide emotional or psychological care for an elderly person; isolation or deprivation of companionship; and inappropriate verbal expression (e.g., calling them names, shouting, and yelling at them).	NA	No	Yes	No
61.	*mICNH (Norwegian)	Blumenfeld Arens et al. (2017)	Modified	Switzerland	S	English	NA	1 Physical, 3 Emotional, 1 Neglect	5	Likert‐scale	4 weeks	Interview‐based	Elder abuse is defined as “a single or repeated act, or lack of appropriate action, occurring within any relationship where there is an expectation of trust that causes harm or distress to an older person”.	10 min	No	Yes	No
62.	*EAQ (Japan)	Shibusawa et al. (2014)	Original	Japan	O	Japanese	NA	2 Physical, 4 Psychological	6	Yes/No	NA	Interview‐based	Elder mistreatment is defined by the National Research Council Panel to Review Risk and Prevalence of Elder Abuse and Neglect as “(a) intentional actions that cause harm or create a serious risk of harm, whether or not intended, to a vulnerable elder by a caregiver or other person who stands in a trust relationship to the elder or (b) failure by a caregiver to satisfy the elder's basic needs or to protect the elder from harm”	NA	No	Yes	No
63.	*mRAAL‐28 items	Botngård et al. (2020)	Modified	Norway	C	English	Norwegian	7 physical, 3 psychological, 5 verbal, 4 material, 4 sexual, 2 caregiving, 3 medication	28	Likert‐scale	3 months	Interview‐based	Older person abuse is an intentional act or failure to act that causes or creates a risk of harm to an older adult.	1 to 1.5 h	No	No	No
64.	*RAAL‐31 items	Castle (2010)	Original	USA	S	English	NA	5 physical, 4 psychological, 5 verbal,5 material, 6 sexual, 3 caregiving, 3 medication	31	Likert‐scale	3 months	Interview‐based	An act of commission or omission that results in harm or threatened harm to the health or welfare of an older adult”.	NA	No	Yes	No
		Castle and Beach (2013)	Original	USA	S	English	NA	5 physical, 4 psychological, 5 verbal,5 material, 6 sexual, 3 caregiving, 3 medication	34	Likert‐scale	3 months	Interview‐based	An act of commission or omission that results in harm or threatened harm to the health or welfare of an older adult”.	NA	No	No	No
65.	DVSQ	Mouton et al. (1999)	Original	USA	O	English	NA	27 Physical	27	Yes/No	12 months	Interview‐based	Domestic violence is defined as all forms of abuse that occur within a relationship including physical, sexual, financial, or psychological abuse	NA	No	No	No
66.	CHCS	Cooper et al. (2013)	Original	UK	S	English	NA	1 Physical, 6 Psychological, 5 Neglect	12	Frequency count	3 months	Self‐report, interview‐based	Abuse is define as “a violation of an individual's human and civil rights by another person(s),” which may not be intentional. Unintentional abuse might include, for example, neglect by a caregiver who was unaware of the needs of a care recipient. Abuse is defined by the impact of actions or inactions on an individual; some actions such as ignoring a care recipient's request may or may not result in abuse, depending on how frequently this occurred, and whether others compensated to meet the person's needs.	NA	Yes	No	No
COMMUNITY AND INSTITUTION																	
67.	OAFEM	Conrad et al. (2009)	Original	USA	S	English	NA	14 Financial	79	Likert‐scale	12 months	Interview‐based	Most broadly, financial exploitation is the illegal or improper use of a vulnerable adult's funds or property for another person's profit or advantage. The usual definition includes a perpetrator who is in a position of trust with the elder) in contrast to quick scams or thievery by strangers.	60 to 90 min	No	Yes	No
		Fang and Yan (2021)	Original	China	O	English	NA	14 Financial	14	NA	NA	Self‐report, interview‐based	NA	60 to 90 min	Yes	No	No
68.	EPAS	Wang et al. (2007)	Original	Taiwan	O	English	NA	32 Psychological	32	Yes/No	NA	Interview‐based	Psychological abuse of elders refers to both intentional and unintentional cases and was defined as follows: failure to respect an elderly person as a human being; failure to provide emotional or psychological care for an elderly person; isolation or deprivation of companionship; and inappropriate verbal expression (e.g., calling them names, shouting, and yelling at them).	NA	No	No	No
		Amirmohammadi et al. (2023)	Original	Iran	O	English	Persian	32 Psychological	32	Yes/No	NA	Interview‐based	Elder abuse is a single or frequent act or failure to take appropriate action that occurs in any relationship in which trust is expected and causes harm or discomfort to an older person	5 to 10 min	No	No	No

We extracted information on the psychometric properties listed in the COSMIN manual, including information on instrument development, content validity, structural validity, internal consistency, cross‐cultural validity, measurement invariance, reliability, measurement error, criterion validity, hypotheses testing for construct validity, and responsiveness.

Information on the definition of AOP used and subtypes measured in the measurement instruments were extracted as the secondary outcome of this review Table 2.

#### 5.3.4 Methodological and quality assessment of included studies

All assessments were performed based on recommendations proposed in the COSMIN guideline (Mokkink et al., 2018). This process involved four steps.

First, we evaluated the methodological quality of the instrument development, content validity and other psychometric properties of the included studies based on the COSMIN Risk of Bias checklist (Mokkink et al., 2018; Prinsen et al., 2018). Second, we evaluated each study's measurement property against the updated criteria for good measurement properties as sufficient (+), insufficient (−) or indeterminate (?). Third, we summarised each instrument's measurement property and then applied the modified Grading of Recommendations, Assessment, Development and Evaluations (GRADE) to determine the quality of the overall evidence for each instrument. Finally, the suitability of these instruments was identified based on evidence of sufficient measurement properties.

##### Assessment of risk of bias

Critical appraisal of studies was performed using the COSMIN Risk of Bias checklist to assess the methodological quality of each study based on the proposed measurement properties (Mokkink et al., 2018). The COSMIN Risk of Bias checklist has 10 boxes covering various study methodology and reporting aspects, as shown in Supporting Information: Appendix 4.

Each study was rated using a four‐point rating system where each standard within a COSMIN criteria box was marked as ‘very good, ‘adequate’, ‘doubtful’, or ‘inadequate’. If multiple studies on different aspects of measurement properties of the same instrument were included, each study was evaluated separately, as each has specific design characteristics. The COSMIN Risk of Bias checklist was used as a modular tool to assess each study by completing the boxes relevant to the psychometric properties evaluated in each study. An overall judgement was made on the methodological quality of each study. The overall rating of the individual study was based on the lowest rating of any standard (i.e., the worst score counts principle) on the checklist.

##### Evaluation of measurement properties based on updated criteria for good measurement properties

Two independent reviewers assessed all study results for each included study and measured according to the COSMIN guidance on updated criteria of good measurement properties (Prinsen et al., 2018). Any discrepancies were solved by consensus among the two reviewers or with the help of a third reviewer.

The content validity study of the measurement instrument was evaluated based on the content validity of the measure itself and the quality of the available studies (Terwee et al., 2018). Content validity was scored as either sufficient (+), insufficient (−), indeterminate (?), or inconsistent (±) based on existing development studies, content validity studies, and reviewer ratings.

The results of each study's other psychometric properties (reliability, validity, and responsiveness) were evaluated according to the updated criteria for good psychometric properties, either as sufficient (above the quality criteria threshold: ‘+’), insufficient (below the quality criteria threshold: ‘−’), or indeterminate (less robust data that do not meet the quality criteria: ‘?’).

The methodological quality of each study on content validity and psychometric properties and the rating of the content validity and psychometric properties results are shown in Tables 3, 4, 5, 6, 7, 8. The summary of findings on the overall rating and quality of the evidence of the relevant psychometric properties for each instrument is shown in the Summary of Findings Table 1.

**Table 3:** Methodological quality of the instrument development and content validity studies.

			Development study quality			Content validity study quality					
			PROM design	Cognitive interview		Asking target population			Asking professionals		
No	Instrument	Reference	Relevance/Comprehensiveness	Comprehensiveness	Comprehensibility	Relevance	Comprehensiveness	Comprehensibility	Relevance	Comprehensiveness	Language
COMMUNITY											
1.	OAPAM	Conrad et al. (2009)	A	A	A	A	A	A	A	A	English
		Alhalal et al. (2022)	NR	NR	NR	NR	NR	NR	D	D	Arabic
2.	UKNPS	McCreadie et al. (2006) O'Keeffe (2007)	A	A	A	NR	NR	NR	NR	NR	English
3.	DEAQ	Heravi‐Karimooi (2010)	D	D	D	D	D	D	D	D	Persian
4.	GMS	Giraldo‐Rodríguez (2013)	A	NR	A	NR	NR	A	D	NR	Spanish
		Pabón‐Poches (2019)	NR	NR	NR	NR	NR	NR	D	D	Spanish
5.	FIVE	Hancock (2023)	D	NR	NR	NR	NR	NR	D	D	English
6.	IEAQ‐Long form	Ghahari et al. (2018)	D	NR	NR	D	D	D	D	D	Persian
7.	MEAS	Hamid Tengku et al. (2013)	I	I	NR	D	D	NR	D	D	Malay
8.	HS‐EAST	Hwalek and Sengstock (1986)	I	NR	NR	NR	NR	NR	I	I	English
		Reichenheim et al. (2008)	NR	NR	NR	NR	NR	NR	A	A	Portuguese
		Jervis et al. (2014)	NR	NR	NR	I	D	I	D	D	English
		Hosseinkhani et al. (2017)	NR	NR	NR	NR	NR	NR	D	I	Persian
		Ozmete (2017)	NR	NR	NR	I	I	I	D	D	Turkish
9.	CASE	Reis and Nahmiash (1995)	I	NR	NR	NR	NR	NR	NR	NR	English
		Paixão et al. (2007)	NR	NR	NR	A	A	A	D	D	Portuguese
10.	EMM	Wong et al. (2021)	I	NR	NR	NR	NR	NR	NR	NR	English
11.	FVOW	Paranjape et al. (2009)	I	NR	NR	NR	NR	NR	NR	NR	English
12.	*IPPA (India)	Bajpai (2023)	I	NR	NR	D	D	NR	NR	NR	English
13.	EACS	Neise et al. (2022)	I	NR	NR	NR	NR	NR	NR	NR	German
14.	AAT	Sembiah et al. (2020)	NR	NR	NR	D	D	D	NR	NR	Bengali
15.	mCTS‐34 items	Luoma (2011)	NR	NR	NR	A	NR	D	NR	NR	English/Dutch/Finnish
		Lang et al. (2014)	NR	NR	NR	D	D	D	D	D	English/Dutch/Finnish
16.	VASS‐12 items	Maia (2014)	NR	NR	NR	NR	NR	D	D	D	Brazilian
		Dantas et al. (2017)	NR	NR	NR	NR	NR	NR	D	NR	Brazilian
		Motahedi et al. (2021)	NR	NR	NR	D	D	D	A	A	Persian
17.**s**	*NAS (Ghana)	Asiamah et al. (2021)	NR	NR	NR	D	D	D	D	D	English
18.	ATDEA	Yi (2019)	NR	NR	NR	D	D	D	NR	NR	Chinese
19.	mGMS (Neglect)	Zawisza et al. (2020)	NR	NR	NR	NR	NR	NR	A	A	Polish
20.	*EA (Korea)	Oh et al. (2006)	NR	NR	NR	NR	NR	D	D	D	Korea
21.	EAS	Karimi and Elahi (2008)	NR	NR	NR	NR	NR	NR	D	D	Persian
22.	*mRAAL‐27 items	Steinsheim et al. (2022)	NR	NR	NR	NR	NR	D	NR	NR	Norwegian
INSTITUTION											
23.	R‐REM	Ramirez et al. (2013)	V	V	V	NR	NR	NR	NR	NR	English
24.	*RAAL‐31 items	Castle (2010)	D	D	D	D	D	D	D	D	English
		Castle and Beach (2013)	D	D	D	D	D	D	D	D	English
25.	*ICNH (Norwegian)	Malmedal (2009)	D	D	D	D	D	D	NR	NR	NR
26.	CHCS	Cooper et al. (2013)	V	D	NR	NR	NR	NR	NR	NR	English
27.	DVSQ	Mouton et al. (1999)	I	NR	NR	NR	NR	NR	D	D	English
28.	*mRAAL‐28 items	Botngård et al. (2020)	NR	NR	NR	D	D	NR	NR	NR	Norwegian
29.	CPEAB	Wang (2005)	NR	NR	NR	NR	NR	NR	D	D	Chinese
30.	*EAS (Korea)	Kim et al. (2006)	NR	NR	NR	NR	NR	NR	D	D	Korean
COMMUNITY AND INSTITUTION											
31.	OAFEM	Conrad et al. (2009)	A	A	A	A	A	A	A	A	English
32.	EPAS	Wang et al. (2007)	A	A	NR	A	A	NR	A	A	Chinese
		Amirmohammadi et al. (2023)	NR	NR	NR	NR	NR	NR	D	NR	Persian

**Table 4:** Quality of content validity for each included study on the development and content validity of an instrument.

			Relevance			Comprehensiveness			Comprehensibility		
No	Instrument	Reference	Development study	Content validity	Reviewer rating	Development study	Content validity	Reviewer rating	Development study	Content validity	Reviewer rating
COMMUNITY											
1.	OAPAM	Conrad et al. (2009)	+	+	+	+	+	+	+	+	+
		Alhalal et al. (2022)	NR	+	+	NR	+	+	NR	NR	NE
2.	UKNPS	McCreadie et al. (2006); O'Keeffe (2007)	+	+	+	+	+	+	+	+	+
3.	DEAQ	Heravi‐Karimooi (2010)	+	+	+	+	+	+	+	+	+
4.	GMS	Giraldo‐Rodríguez (2013)	+	+	+	NR	NR	NR	+	+	+
		Pabón‐Poches (2019)	NR	+	+	NR	+	+	NR	NR	NE
5.	MEAS	Hamid Tengku et al. (2013)	?	?	−	?	?	−	NR	NR	NE
6.	FIVE	Hancock (2023)	?	?	−	NR	+	+	NR	NR	NE
7.	IEAQ‐Long form	Ghahari et al. (2018)	?	?	−	?	?	−	?	?	−
8.	HS‐EAST	Hwalek and Sengstock (1986)	−	+	+	NR	+	+	NR	NR	NE
		Reichenheim et al. (2008)	NR	+	+	NR	+	+	NR	+	+
		Jervis et al. (2014)	NR	+	+	NR	+	+	NR	?	−
		Hosseinkhani et al. (2017)	NR	+	+	NR	+	+	NR	NR	NE
		Ozmete (2017)	NR	+	+	NR	+	+	NR	?	−
9.	CASE	Reis and Nahmiash (1995)	−	NR	−	NR	NR	NE	NR	NR	NE
		Paixão et al. (2007)	NR	+	+	NR	?	+	NR	+	+
10.	EMM	Wong et al. (2021)	−	NR	−	NR	NR	NE	NR	NR	NE
11.	FVOW	Paranjape et al. (2009)	?	NR	−	NR	NR	NE	NR	NR	NE
12.	*IPPA (India)	Bajpai (2023)	?	?	−	?	?	−	NR	NR	NE
13.	EACS	Neise et al. (2022)	−	NR	−	NR	NR	NE	NR	NR	NE
14.	AAT	Sembiah et al. (2020)	NR	?	−	NR	?	−	NR	?	−
15.	mCTS‐34 items	Luoma (2011)	NR	−	−	NR	NR	NE	NR	−	−
		Lang et al. (2014)	NR	−	−	NR	+	+	NR	−	−
16.	VASS‐12 items	Maia (2014)	NR	+	+	NR	+	+	NR	?	−
		Dantas et al. (2017)	NR	NR	NE	NR	NR	NE	NR	+	+
		Motahedi et al. (2021)	NR	+	+	NR	+	+	NR	+	+
17.	*NAS (Ghana)	Asiamah et al. (2021)	NR	?	−	NR	+	+	NR	?	−
18.	ATDEA	Yi (2019)	NR	?	+	NR	?	−	NR	?	−
19.	mGMS (Neglect)	Zawisza et al. (2020)	NR	+	+	NR	+	+	NR	NR	NE
20.	*EA (Korea)	Oh et al. (2006)	NR	?	−	NR	?	_	NR	?	−
21.	EAS	Karimi and Elahi (2008)	NR	?	−	NR	?	−	NR	NR	NE
22.	*mRAAL‐27 items	Steinsheim et al. (2022)	NR	NR	NE	NR	NR	NE	NR	+	+
INSTITUTION											
23.	R‐REM	Ramirez et al. (2013)	+	+	+	+	+	+	+	+	+
24.	*RAAL‐31 items	Castle (2010)	+	+	+	+	+	+	+	+	+
		Castle and Beach (2013)	+	+	+	+	+	+	+	+	+
25.	*ICNH (Norwegian)	Malmedal (2009)	+	+	+	?	?	−	+	+	+
26.	CHCS	Cooper et al. (2013)	+	NR	+	+	NR	+	NR	NR	NE
27.	DVSQ	Mouton et al. (1999)	?	+	−	?	?	−	NR	NR	NE
28.	*mRAAL‐28 items	Botngård et al. (2020)	NR	+	+	NR	+	+	NR	NR	NE
29.	CPEAB	Wang (2005)	NR	?	−	NR	?	−	NR	NR	NE
30.	*EAS (Korea)	Kim et al. (2006)	NR	?	−	NR	?	−	NR	NR	NE
COMMUNITY AND INSTITUTION											
31.	OAFEM	Conrad et al. (2009)	+	+	+	+	+	+	+	+	+
32.	EPAS	Wang et al. (2007)	+	+	+	+	+	+	NR	NR	NE
		Amirmohammadi et al. (2023)	NR	+	+	NR	NR	NE	NR	NR	NE

**Table 5:** Overall methodological and quality assessment of development and content validity studies per instrument.

		Content validity		*Relevance*		*Comprehensiveness*		*Comprehensibility*	
	Instrument	Overall quality	Quality of evidence	Overall quality	Quality of evidence	Overall quality	Quality of evidence	Overall quality	Quality of evidence
COMMUNITY									
1.	OAPAM	+	Moderate	+	Moderate	+	Moderate	+	Moderate
2.	GMS	+	Moderate	+	Moderate	+	Moderate	+	Moderate
3.	UKNPS	+	Moderate	+	Moderate	+	Moderate	+	Moderate
4.	DEAQ	+	Low	+	Low	+	Low	+	Low
5.	HS‐EAST	+	Low	+	Low	?	Low	+	Low
6.	VASS‐12 items	+	Low	−	Low	+	Low	±	Low
7.	IEAQ‐Long form	−	Low	−	Low	−	Low	−	Low
8.	mCTS‐34 items	−	Low	−	Low	−	Low	−	Low
9.	ATDEA	−	Low	+	Low	−	Low	−	Low
10.	CASE	±	Inconsistent	−	Low	+	Moderate	+	Moderate
11.	*NAS (Ghana)	±	Inconsistent	±	Low	+	Low	−	Low
12.	mGMS (Neglect)	NE	NE	+	Moderate	+	Moderate	NE	NE
13.	MEAS	NE	NE	−	Low	−	Low	NE	NE
14.	FIVE	NE	NE	−	Low	+	Low	NE	NE
15.	*EA (Korea)	NE	NE	−	Low	−	Low	NE	NE
16.	EAS	NE	NE	−	Low	−	Low	NE	NE
17.	*IPPA (India)	NE	NE	−	Very Low	−	Very Low	NE	NE
18.	EMM	NE	NE	−	Very Low	NE	NE	NE	NE
19.	FVOW	NE	NE	−	Very Low	NE	NE	NE	NE
20.	EACS	NE	NE	−	Very Low	NE	NE	NE	NE
21.	*mRAAL‐27 items	NE	NE	NE	NE	NE	NE	+	Low
INSTITUTION									
22.	R‐REM	+	Moderate	+	Moderate	+	Moderate	+	Moderate
23.	*RAAL‐31 items	+	Low	+	Low	+	Low	+	Low
24.	*mRAAL‐28 items	+	Low	+	Low	+	Low	+	Low
25.	*ICNH (Norwegian)	±	Inconsistent	+	Low	−	Low	+	Low
26.	CHCS	NE	NE	+	High	+	Low	NE	NE
27.	DVSQ	NE	NE	+	Very Low	−	Very Low	NE	NE
28.	*EAS (Korea)	NE	NE	−	Low	−	Low	NE	NE
29.	CPEAB	NE	NE	−	Low	−	Low	NE	NE
COMMUNITY AND INSTITUTION									
30.	OAFEM	+	Moderate	+	Moderate	+	Moderate	+	Moderate
31.	EPAS	NE	NE	+	Moderate	+	Moderate	NE	NE

**Table 6:** Methodological quality assessment of psychometric studies.

No	Instrument	Reference	Structural validity	Internal consistency	Cross‐cultural validity	Measurement invariance	Reliability	Criterion Validity	Construct validity/Hypothesis testing	Language
COMMUNITY										
1.	CASE	Reis and Nahmiash (1995)	D	I	NR	NR	NR	NR	V, V	English
		Reichenheim et al. (2009)	V	V	NR	NR	A	NR	V, NR	English
		冯瑞新 (2010)	V	I	NR	NR	I	NR	NR	English
		Pérez‐Rojo et al. (2015)	V	V	NR	NR	NR	NR	V, NR	English
		Melchiorre et al. (2017)	V	V	NR	NR	NR	NR	V, NR	English
		Rivera‐Navarro (2018)	V	V	NR	NR	NR	NR	V, V	English
		Sakar et al. (2019)	A	V	NR	NR	NR	NR	V, NR	Persian
		Khan et al. (2020)	V	V	V	V	NR	NR	V, V	Urdu
2.	VASS‐12 items	Schofield (2002)	V	V	NR	NR	NR	NR	V, NR	English
		Schofield and Mishra (2003)	V	NR	NR	NR	NR	NR	V, NR	English
		Buri et al. (2009)	NR	I	NR	NR	I	V	NR	English
		Dong and Simon (2013)	NR	I	NR	NR	NR	NR	NR	Mandarin
		Maia (2016)	NR	V	NR	NR	NR	NR	NR	Portuguese
		Asiret (2017)	V	V	NR	NR	I	NR	V, NR	Turkish
		Dantas et al. (2017)	V	V	NR	NR	NR	NR	V, NR	Turkish
		Motahedi et al. (2021)	V	V	NR	NR	I	NR	NR	Persian
3.	HS‐EAST	Neale (1991)	NR	I	NR	NR	NR	NR	NR, V	English
		Moody (2000)	NR	V	NR	NR	NR	NR	NR, V	English
		Reichenheim et al. (2008)	V	V	NR	NR	V	NR	NR	Portuguese
		Buri et al. (2009)	I	I	NR	NR	I	V	NR	English
		Jervis et al. (2014)	NR	I	NR	NR	NR	NR	V, A	English
		Hosseinkhani et al. (2017)	I	I	NR	NR	I	NR	NR	Persian
		Özçakar et al. (2017)	I	V	NR	NR	I	V	V, NR	Turkish
		Ozmete (2017)	NR	V	NR	NR	NR	NR	NR	Turkish
		Akyol Guner (2022)	NR	I	NR	NR	NR	NR	NR	Turkish
		Atim et al. (2023)	NR	I	NR	NR	NR	NR	NR	Uganda local language
4.	mGMS (Neglect)	Zawisza et al. (2020)	V	V	NR	NR	I	NR	V, V	Polish
5.	GMS	Giraldo‐Rodríguez (2013)	NR	V	NR	NR	NR	NR	A, NR	English
		Giraldo‐Rodríguez (2015)	NR	I	NR	NR	NR	NR	NR	English
		Dasbas (2019)	V	V	NR	NR	NR	NR	NR	English
		Rashidi Fakari (2020)	V	I	NR	NR	I	NR	NR	English
6.	mCTS‐M 38 items	Wazid et al. (2021)	V	V	NR	NR	NR	NR	NR	Malay
		Sooryanarayana et al. (2017)	NR	NR	NR	NR	NR	NR	I, I	English
7.	DEAQ	Heravi‐Karimooi (2010)	V	V	NR	NR	D	NR	NR	Persian
		Keyghobadi et al. (2014)	NR	V	NR	NR	NR	NR	NR	Persian
		Morowatisharifabad et al. (2016)	NR	V	NR	NR	NR	NR	NR	Persian
8.	FIVE	Hancock (2023)	V	V	NR	NR	NR	NR	V, NR	English
9.	MEAS	Hamid Tengku et al. (2013)	V	V	NR	NR	NR	NR	V, NR	Malay
10.	*NAS (Ghana)	Asiamah et al. (2021)	V	V	NR	NR	NR	NR	V, NR	English
11.	EACS	Neise et al. (2022)	V	V	NR	NR	NR	NR	V, NR	German
12.	Native EAS‐Short Form	Ghahari et al. (2018)	V	V	NR	NR	NR	NR	V, NR	English
13.	FVOW	Paranjape et al. (2009)	V	I	NR	NR	NR	NR	V, NR	English
14.	mVASS‐15 items	徐金燕 and 蒋利平 (2020)	V	V	NR	NR	NR	NR	NR	English
15.	FVS	Préville et al. (2014)	V	I	NR	NR	NR	NR	NR	French
16.	*IPPA (India)	Bajpai (2023)	V	I	NR	NR	NR	NR	NR	Tamil
17.	IEAQ‐Long form	Ghahari et al. (2018)	V	V	NR	NR	NR	NR	NR	English
18.	EVEQ	Ajdikovic (2008)	V	V	NR	NR	NR	NR	NR	English
19.	EMM	Wong et al. (2021)	V	V	NR	NR	NR	NR	NR	English
20.	*EAN (Puerto Ricans)	Irizarry‐Irizarry (2008)	V	V	NR	NR	NR	NR	NR	Spanish
21.	EMS	Wu (2012)	I	I	NR	NR	NR	NR	NR	Chinese
22.	WHRS	Fisher and Regan (2006)	I	V	NR	NR	NR	NR	NR	English
23.	*IPVQ	Charro‐Baena et al. (2023)	V	NR	NR	NR	NR	NR	NR	Spanish
24.	*mNSEAN (Neglect)	Ayalon (2015)	NR	V	NR	V	NR	NR	NR	English
25.	mOARS‐ADL(Neglect)	Fang and Yan (2021)	NR	V	NR	NR	NR	NR	V, NR	English
26.	mCTS‐C 25 Items	Yan and Tang (2001)	NR	D	NR	NR	NR	NR	NR	Chinese
		Yan and Tang (2004)	NR	V	NR	NR	NR	NR	V, NR	Chinese
27.	mCTS‐10 items	Beach et al. (2005)	NR	I	NR	NR	NR	NR	NR	English
		Cooper, Manela, et al. (2008)	NR	I	NR	NR	NR	V	NR	English
		Lafferty (2016)	NR	I	NR	NR	NR	NR	NR	English
		Qin and Yan (2018)	NR	I	NR	NR	NR	NR	NR	NA
28.	*mNSEAN	Ayalon (2011)	NR	I	NR	NR	NR	NR	V, NR	English
29.	*EANQ (Iran)	Manoochehri et al. (2008)	NR	I	NR	NR	I	NR	NR	Persian
30.	mCTS‐T 18 items	Chokkanathan (2005)	NR	V	NR	NR	NR	NR	NR	Tamil
31.	mCTS‐C 18 items	Yan and Kwok (2011)	NR	V	NR	NR	NR	NR	NR	NA
32.	mCTS‐C 23 items	Chen and Chan (2022)	NR	V	NR	NR	NR	NR	NR	Chinese
33.	ABUEL	Melchiorre et al. (2013)	NR	V	NR	NR	NR	NR	NR	English
		Melchiorre et al. (2016)	NR	V	NR	NR	NR	NR	NR	English
34.	EAVQ	Kong and Jeon (2018)	NR	V	NR	NR	NR	NR	NR	English
35.	USCOACS	DeLiema et al. (2012)	NR	V	NR	NR	NR	NR	NR	Spanish
36.	m‐EAQ	Chang (2016)	NR	V	NR	NR	NR	NR	NR	Korean
37.	mCTS‐Verbal 12 items	Fulmer et al. (2014)	NR	I	NR	NR	NR	NR	NR	English
38.	mVASS‐10 items	Dong et al. (2014)	NR	I	NR	NR	NR	NR	NR	English
39.	mVASS‐16 items	钱振中 (2016)	NR	I	NR	NR	NR	NR	NR	Mandarin
40.	m‐HS‐EAST	Alizadeh‐Khoei et al. (2014)	NR	I	NR	NR	NR	NR	NR	Farsi
41.	ATDEA	Yang et al. (2022)	NR	I	NR	NR	NR	NR	NR	Chinese
42.	*EA (Korea)	Oh et al. (2006)	NR	I	NR	NR	NR	NR	NR	English
43.	*DVE (Thailand)	Panjaphothiwat (2021)	NR	I	NR	NR	NR	NR	NR	English
44.	*EAQ (Nepal)	Yadav et al. (2018)	NR	I	NR	NR	NR	NR	NR	English
45.	*EAQ (Iran)	Honarvar et al. (2020)	NR	V	NR	NR	NR	NR	NR	NA
46.	AAT	Sembiah et al. (2020)	NR	I	NR	NR	NR	NR	NR	Bengali
47.	m‐EASI	Badenes‐Ribera et al. (2021)	NR	I	NR	NR	NR	NR	NR	English
48.	QEEA	Badenes‐Ribera et al. (2021)	NR	I	NR	NR	NR	NR	NR	English
49.	*EAQ (China)	Su et al. (2011)	NR	NR	NR	NR	I	NR	NR	Chinese
50.	EAS	Karimi and Elahi (2008)	NR	NR	NR	NR	I	NR	NR	English
51.	FPS‐2 items	Chan et al. (2019)	NR	NR	NR	NR	NR	V	NR	English
52.	mCTS‐34 items	Lang et al. (2014)	NR	NR	NR	NR	NR	NR	V, NR	English
53.	mFPS‐5 items	Kita et al. (2023)	NR	NR	NR	NR	NR	NR	V, NR	English
54.	*mRAAL‐27 items	Steinsheim et al. (2022)	NR	NR	NR	NR	NR	NR	NR	Norwegian
55.	UKNPS	McCreadie et al. (2006) O'Keeffe (2007)	NR	NR	NR	NR	NR	NR	NR	English
INSTITUTION										
56.	OAPAM	Conrad et al. (2009)	V	V	NR	NR	NR	NR	NR, V	English
		Alhalal et al. (2022)	NR	V	NR	NR	NR	NR	NR	Arabic
57.	R‐REM	Teresi et al. (2014)	V	V	NR	NR	NR	NR	NR	English
58.	*EAS (Korea)	Kim et al. (2006)	V	V	NR	NR	NR	NR	NR	Korean
		Lee (2008)	NR	I	NR	NR	NR	NR	NR	English
59.	*ICNH (Norwegian)	Malmedal (2014)	V	V	NR	NR	NR	NR	NR	English
60.	CPEAB	Wang (2005)	NR	V	NR	NR	I	NR	NR	English
61.	*mICNH (Norwegian)	Blumenfeld Arens et al. (2017)	NR	V	NR	NR	NR	NR	NR	English
62.	*EAQ (Japan)	Shibusawa et al. (2014)	NR	V	NR	NR	NR	NR	NR	Japanese
63.	*mRAAL‐28 items	Botngård et al. (2020)	NR	I	NR	NR	NR	NR	NR	Norwegian
64.	*RAAL‐31 items	Castle (2010) Castle and Beach (2013)	NR	NR	NR	NR	NR	NR	NR	English
65.	DVSQ	Mouton et al. (1999)	NR	NR	NR	NR	NR	NR	NR	English
66.	CHCS	Cooper et al. (2013)	NR	NR	NR	NR	NR	NR	NR	English
COMMUNITY AND IN INSTITUTION										
67.	OAFEM	Conrad et al. (2009)	V	V	NR	NR	NR	NR	NR, V	English
		Fang and Yan (2021)	NR	V	NR	NR	NR	NR	NR	English
68.	EPAS	Wang et al. (2007)	NR	V	NR	NR	A	NR	V, NR	English
		Amirmohammadi et al. (2023)	NR	V	NR	NR	I	NR	NR	English

**Table 7:** Quality of the psychometric properties of each included study.

No	Instrument	Reference	Structural validity	Internal consistency	Cross‐cultural validity	Measurement invariance	Reliability	Criterion validity	Construct validity/Hypothesis testing
COMMUNITY									
1.	CASE	Reis and Nahmiash (1995)	?	+	NR	NR	NR	NR	+,+
		Reichenheim et al. (2009)	+	+	NR	NR	+	NR	+, NR
		冯瑞新 (2010)	+	+	NR	NR	?	NR	NR
		Pérez‐Rojo et al. (2015)	?	+	NR	NR	NR	NR	+, NR
		Melchiorre et al. (2017)	+	+	NR	NR	NR	NR	+, NR
		Rivera‐Navarro (2018)	+	+	NR	NR	NR	NR	+,+
		Sakar et al. (2019)	+	+	NR	NR	NR	NR	+, NR
		Khan et al. (2020)	+	+	+	+	NR	NR	+,+
2.	VASS‐12 items	Schofield (2002)	+	−	NR	NR	NR	NR	+, NR
		Schofield and Mishra (2003)	+	NR	NR	NR	NR	NR	+, NR
		Buri et al. (2009)	NR	+	NR	NR	?	−	NR
		Dong and Simon (2013)	NR	+	NR	NR	NR	NR	NR
		Maia (2016)	NR	+	NR	NR	NR	NR	NR
		Asiret (2017)	+	+	NR	NR	?	NR	+, NR
		Dantas et al. (2017)	+	+	NR	NR	NR	NR	+, NR
		Motahedi et al. (2021)	?	+	NR	NR	?	NR	NR
3.	HS‐EAST	Neale (1991)	NR	?	NR	NR	NR	NR	NR,+
		Moody (2000)	NR	?	NR	NR	NR	NR	NR, +
		Reichenheim et al. (2008)	_	?	NR	NR	+	NR	NR
		Buri et al. (2009)	?	?	NR	NR	?	−	NR
		Jervis et al. (2014)	NR	?	NR	NR	NR	NR	+, +
		Hosseinkhani et al. (2017)	?	?	NR	NR	?	NR	NR
		Özçakar et al. (2017)	+	?	NR	NR	?	+	+, NR
		Ozmete (2017)	NR	?	NR	NR	NR	NR	NR
		Akyol Guner (2022)	NR	?	NR	NR	NR	NR	NR
		Atim et al. (2023)	NR	?	NR	NR	NR	NR	NR
4.	mGMS (Neglect)	Zawisza et al. (2020)	+	+	NR	NR	?	NR	+,+
5.	GMS	Giraldo‐Rodríguez (2013)	NR	?	NR	NR	NR	NR	+, NR
		Giraldo‐Rodríguez (2015)	NR	?	NR	NR	NR	NR	NR
		Dasbas (2019)	+	?	NR	NR	NR	NR	NR
		Rashidi Fakari (2020)	−	?	NR	NR	+	NR	NR
6.	mCTS‐M 38 items	Wazid et al. (2021)	+	+	NR	NR	NR	NR	NR
		Sooryanarayana (2017)	NR	NR	NR	NR	NR	NR	+,+
7.	DEAQ	Heravi‐Karimooi (2010)	+	+	NR	NR	?	NR	NR
		Keyghobadi et al. (2014)	NR	+	NR	NR	NR	NR	NR
		Morowatisharifabad et al. (2016)	NR	+	NR	NR	NR	NR	NR
8.	FIVE	Hancock (2023)	+	−	NR	NR	NR	NR	+, NR
9.	MEAS	Hamid Tengku et al. (2013)	+	−	NR	NR	NR	NR	+, NR
10.	*NAS (Ghana)	Asiammah (2021)	+	+	NR	NR	NR	NR	+, NR
11.	EACS	Neise et al. (2022)	+	+	NR	NR	NR	NR	+, NR
12.	Native EAS‐Short Form	Ghahari et al. (2018)	+	+	NR	NR	NR	NR	+, NR
13.	FVOW	Paranjape et al. (2009)	−	+	NR	NR	NR	NR	?, NR
14.	mVASS‐15 items	徐金燕 and 蒋利平 (2020)	+	+	NR	NR	NR	NR	NR
15.	FVS	Préville et al. (2014)	+	+	NR	NR	NR	NR	NR
16.	*IPPA (India)	Bajpai (2023)	+	+	NR	NR	NR	NR	NR
17.	IEAQ‐Long form	Ghahari et al. (2018)	+	−	NR	NR	NR	NR	NR
18.	EVEQ	Ajdikovic (2008)	−	+	NR	NR	NR	NR	NR
19.	EMM	Wong et al. (2021)	?	_	NR	NR	NR	NR	NR
20.	*EAN (Puerto Ricans)	Irizarry‐Irizarry (2008)	?	+	NR	NR	NR	NR	NR
21.	EMS	Wu (2012)	+	?	NR	NR	NR	NR	NR
22.	WHRS	Fisher and Regan (2006)	?	?	NR		NR	NR	NR
23.	*IPVQ	Charro‐Baena et al. (2023)	?	NR	NR	NR	NR	NR	NR
24.	*mNSEAN (Neglect)	Ayalon (2015)	NR	?	NR	+	NR	NR	NR
25.	mOARS‐ADL(Neglect)	Fang and Yan (2021)	NR	?	NR	NR	NR	NR	+, NR
26.	mCTS‐C 25 Items	Yan and Tang (2001)	NR	?	NR	NR	NR	NR	NR
27.	mCTS‐10 items	Beach et al. (2005)	NR	?	NR	NR	NR	NR	NR
		Cooper, Manela, et al. (2008)	NR	?	NR	NR	NR	+	NR
		Lafferty (2016)	NR	?	NR	NR	NR	NR	NR
		Qin and Yan (2018)	NR	?	NR	NR	NR	NR	NR
28.	*mNSEAN	Ayalon (2011)	NR	?	NR	NR	NR	NR	+, NR
29.	*EANQ (Iran)	Manoochehri et al. (2008)	NR	?	NR	NR	?	NR	NR
30.	mCTS‐T 18 items	Chokkanathan (2005)	NR	?	NR	NR	NR	NR	NR
31.	mCTS‐C 18 items	Yan and Kwok (2011)	NR	?	NR	NR	NR	NR	NR
32.	mCTS‐C 23 items	Chen and Chan (2022)	NR	?	NR	NR	NR	NR	NR
33.	ABUEL	Melchiorre et al. (2013)	NR	?	NR	NR	NR	NR	NR
		Melchiorre et al. (2016)	NR	?	NR	NR	NR	NR	NR
34.	EAVQ	Kong and Jeon (2018)	NR	?	NR	NR	NR	NR	NR
35.	USCOACS	DeLiema et al. (2012)	NR	?	NR	NR	NR	NR	NR
36.	m‐EAQ	Chang (2016)	NR	?	NR	NR	NR	NR	NR
37.	mCTS‐Verbal 12 items	Fulmer et al. (2014)	NR	?	NR	NR	NR	NR	NR
38.	mVASS‐10 items	Dong et al. (2014)	NR	?	NR	NR	NR	NR	NR
39.	mVASS‐16 items	钱振中 (2016)	NR	?	NR	NR	NR	NR	NR
40.	m‐HS‐EAST	Alizadeh‐Khoei et al. (2014)	NR	?	NR	NR	NR	NR	NR
41.	ATDEA	Yang et al. (2022)	NR	?	NR	NR	NR	NR	NR
42.	*EA (Korea)	Oh et al. (2006)	NR	?	NR	NR	NR	NR	NR
43.	*DVE (Thailand)	Panjaphothiwat (2021)	NR	?	NR	NR	NR	NR	NR
44.	*EAQ (Nepal)	Yadav et al. (2018)	NR	?	NR	NR	NR	NR	NR
45.	*EAQ (Iran)	Honarvar et al. (2020)	NR	?	NR	NR		NR	NR
46.	AAT	Sembiah et al. (2020)	NR	?	NR	NR	NR	NR	NR
47.	m‐EASI	Badenes‐Ribera et al. (2021)	NR	?	NR	NR	NR	NR	NR
48.	QEEA	Badenes‐Ribera et al. (2021)	NR	?	NR	NR	NR	NR	NR
49.	*EAQ (China)	Su et al. (2011)	NR	NR	NR	NR	+	NR	NR
50.	EAS	Karimi and Elahi (2008)		NR	NR	NR	?	NR	NR
51.	FPS‐2 items	Chan et al. (2019)	NR	NR	NR	NR	NR	−	NR
52.	mCTS‐34 items	Lang et al. (2014)	NR	NR	NR	NR	NR	NR	+, NR
53.	mFPS‐5 items	Kita et al. (2023)	NR	NR	NR	NR	NR	NR	?, NR
54.	*mRAAL‐27 items	Steinsheim et al. (2022)	NR	NR	NR	NR	NR	NR	NR
55.	UKNPS	McCreadie et al. (2006) O'Keeffe (2007)	NR	NR	NR	NR	NR	NR	NR
INSTITUTION									
56.	OAPAM	Conrad et al. (2009)	+	+	NR	NR	NR	NR	NR, +
		Alhalal et al. (2022)	NR	+	NR	NR	NR	NR	NR
57.	R‐REM	Teresi et al. (2014)	+	+	NR	NR	NR	NR	NR
58.	*EAS (Korea)	Kim et al. (2006)	+	+	NR	NR	NR	NR	NR
		Lee (2008)	NR	+	NR	NR	NR	NR	NR
59.	*ICNH (Norwegian)	Malmadel (2014)	−	+	NR	NR	NR	NR	NR
60.	CPEAB	Wang (2005)	NR	?	NR	NR	−	NR	NR
61.	*mICNH (Norwegian)	Blumenfeld Arens et al. (2017)	NR	?	NR	NR	NR	NR	NR
62.	*EAQ (Japan)	Shibusawa et al. (2014)	NR	?	NR	NR	NR	NR	NR
63.	*mRAAL‐28 items	Botngård et al. (2020)	NR	?	NR	NR	NR	NR	NR
64.	*RAAL‐31 items	Castle (2010) Castle and Beach (2013)	NR	NR	NR	NR	NR	NR	NR
65.	DVSQ	Mouton et al. (1999)	NR	NR	NR	NR	NR	NR	NR
66.	CHCS	Cooper et al. (2013)	NR	NR	NR	NR	NR	NR	NR
COMMUNITY AND INSTITUTION									
67.	OAFEM	Conrad et al. (2009)	+	+	NR	NR	NR	NR	NR, +
		Fang and Yan (2021)	NR	+	NR	NR	NR	NR	NR
68.	EPAS	Wang et al. (2007)	NR	?	NR	NR	+	NR	+, NR
		Amirmohammadi et al. (2023)	NR	?	NR	NR	+	NR	NR

**Table 8:** Overall quality of the psychometric properties and evidence quality per instrument.

		Structural validity		Internal consistency		Cross‐cultural validity		Measurement invariance		Reliability		Criterion validity		Hypothesis testing	
No	Instrument	Overall rating	Quality of evidence	Overall rating	Quality of evidence	Overall rating	Quality of evidence	Overall rating	Quality of evidence	Overall rating	Quality of evidence	Overall rating	Quality of evidence	Overall rating	Quality of evidence
COMMUNITY															
1.	CASE	+	Moderate	+	Moderate	+	Moderate	+	High	±	Inconsistent	NE	NE	+	High
2.	VASS‐12 items	+	High	+	Moderate	NE	NE	NE	NE	?	Very Low	−	High	+	High
3.	HS‐EAST	±	Inconsistent	?	Very Low	NE	NE	NE	NE	?	Low	±	Inconsistent	+	High
4.	mGMS (Neglect)	+	High	+	High	NE	NE	NE	NE	?	Very Low	NE	NE	+	High
5.	GMS	±	Inconsistent	?	Very Low	NE	NE	NE	NE	+	Very Low	NE	NE	+	Moderate
6.	mCTS‐M 38 items	+	High	+	High	NE	NE	NE	NE	NE	NE	NE	NE	+	Very Low
7.	DEAQ	+	High	+	High	NE	NE	NE	NE	?	Low	NE	NE	NE	NE
8.	FIVE	+	High	−	High	NE	NE	NE	NE	NE	NE	NE	NE	+	High
9.	MEAS	+	High	−	High	NE	NE	NE	NE	NE	NE	NE	NE	+	High
10.	*NAS (Ghana)	+	High	+	High	NE	NE	NE	NE	NE	NE	NE	NE	+	High
11.	EACS	+	High	+	High	NE	NE	NE	NE	NE	NE	NE	NE	+	High
12.	Native EAS‐Short Form	+	High	+	High	NE	NE	NE	NE	NE	NE	NE	NE	+	High
13.	FVOW	−	High	+	Very Low	NE	NE	NE	NE	NE	NE	NE	NE	?	High
14.	mVASS‐15 items	+	High	+	High	NE	NE	NE	NE	NE	NE	NE	NE	NE	NE
15.	FVS	+	High	+	Very low	NE	NE	NE	NE	NE	NE	NE	NE	NE	NE
16.	*IPPA (India)	+	High	+	Very Low	NE	NE	NE	NE	NE	NE	NE	NE	NE	NE
17.	IEAQ‐Long form	+	High	−	High	NE	NE	NE	NE	NE	NE	NE	NE	NE	NE
18.	EVEQ	−	High	+	High	NE	NE	NE	NE	NE	NE	NE	NE	NE	NE
19.	EMM	?	High	−	High	NE	NE	NE	NE	NE	NE	NE	NE	NE	NE
20.	*EAN (Puerto Ricans)	?	High	+	High	NE	NE	NE	NE	NE	NE	NE	NE	NE	NE
21.	EMS	+	Very Low	+	Very Low	NE	NE	NE	NE	NE	NE	NE	NE	NE	NE
22.	WHRS	?	Very Low	?	High	NE	NE	NE	NE	NE	NE	NE	NE	NE	NE
23.	*IPVQ	?	High	NE	NE	NE	NE	NE	NE	NE	NE	NE	NE	NE	NE
24.	*mNSEAN (Neglect)	NE	NE	?	High	NE	NE	+	High	NE	NE	NE	NE	NE	NE
25.	mOARS‐ADL(Neglect)	NE	NE	?	High	NE	NE	NE	NE	NE	NE	NE	NE	+	High
26	mCTS‐C 25 Items	NE	NE	?	Moderate	NE	NE	NE	NE	NE	NE	NE	NE	+	High
27.	mCTS‐10 items	NE	NE	?	Very Low	NE	NE	NE	NE	NE	NE	+	High	NE	NE
28.	*mNSEAN	NE	NE	?	Very Low	NE	NE	NE	NE	NE	NE	NE	NE	+	High
29.	*EANQ (Iran)	NE	NE	?	Very Low	NE	NE	NE	NE	?	Very Low	NE	NE	NE	NE
30.	mCTS‐T 18 items	NE	NE	?	High	NE	NE	NE	NE	NE	NE	NE	NE	NE	NE
31.	mCTS‐C 18 items	NE	NE	?	High	NE	NE	NE	NE	NE	NE	NE	NE	NE	NE
32.	mCTS‐C 23 items	NE	NE	?	High	NE	NE	NE	NE	NE	NE	NE	NE	NE	NE
33.	ABUEL	NE	NE	?	High	NE	NE	NE	NE	NE	NE	NE	NE	NE	NE
34.	EAVQ	NE	NE	?	High	NE	NE	NE	NE	NE	NE	NE	NE	NE	NE
35.	USCOACS	NE	NE	?	High	NE	NE	NE	NE	NE	NE	NE	NE	NE	NE
36.	m‐EAQ	NE	NE	?	High	NE	NE	NE	NE	NE	NE	NE	NE	NE	NE
37.	mCTS‐Verbal 12 items	NE	NE	?	Very Low	NE	NE	NE	NE	NE	NE	NE	NE	NE	NE
38.	mVASS‐10 items	NE	NE	?	Very Low	NE	NE	NE	NE	NE	NE	NE	NE	NE	NE
39.	mVASS‐16 items	NE	NE	?	Very Low	NE	NE	NE	NE	NE	NE	NE	NE	NE	NE
40.	m‐HS‐EAST	NE	NE	?	Very Low	NE	NE	NE	NE	NE	NE	NE	NE	NE	NE
41.	ATDEA	NE	NE	?	Very Low	NE	NE	NE	NE	NE	NE	NE	NE	NE	NE
42.	*EA (Korea)	NE	NE	?	Very Low	NE	NE	NE	NE	NE	NE	NE	NE	NE	NE
43.	*DVE (Thailand)	NE	NE	?	Very Low	NE	NE	NE	NE	NE	NE	NE	NE	NE	NE
44.	*EAQ (Nepal)	NE	NE	?	Very Low	NE	NE	NE	NE	NE	NE	NE	NE	NE	NE
45.	*EAQ (Iran)	NE	NE	?	Very Low	NE	NE	NE	NE	NE	NE	NE	NE	NE	NE
46.	AAT	NE	NE	?	Very Low	NE	NE	NE	NE	NE	NE	NE	NE	NE	NE
47.	m‐EASI	NE	NE	?	Very Low	NE	NE	NE	NE	NE	NE	NE	NE	NE	NE
48.	QEEA	NE	NE	?	Very Low	NE	NE	NE	NE	NE	NE	NE	NE	NE	NE
49.	*EAQ (China)	NE	NE	NE	NE	NE	NE	NE	NE	+	Very Low	NE	NE	NE	NE
50.	EAS	NE	NE	NE	NE	NE	NE	NE	NE	?	Very Low	NE	NE	NE	NE
51.	FPS‐2 items	NE	NE	NE	NE	NE	NE	NE	NE	NE	NE	NE	High	NE	NE
52.	mCTS‐34 items	NE	NE	NE	NE	NE	NE	NE	NE	NE	NE	NE	NE	+	High
53.	mFPS‐5 items	NE	NE	NE	NE	NE	NE	NE	NE	NE	NE	NE	NE	?	Moderate
INSTITUTION															
54.	OAPAM	+	High	+	High	NE	NE	NE	NE	NE	NE	NE	NE	+	High
55.	R‐REM	+	High	+	High	NE	NE	NE	NE	NE	NE	NE	NE	NE	NE
56.	*EAS (Korea)	+	High	+	Low	NE	NE	NE	NE	NE	NE	NE	NE	NE	NE
57.	*ICNH (Norwegian)	−	Moderate	+	High	NE	NE	NE	NE	NE	NE	NE	NE	NE	NE
58.	CPEAB	NE	NE	?	High	NE	NE	NE	NE	NE	Very low	NE	NE	NE	NE
59.	*mICNH (Norwegian)	NE	NE	?	High	NE	NE	NE	NE	NE	NE	NE	NE	NE	NE
60.	*EAQ (Japan)	NE	NE	?	High	NE	NE	NE	NE	NE	NE	NE	NE	NE	NE
61.	*mRAAL‐28 items	NE	NE	?	Very Low	NE	NE	NE	NE	NE	NE	NE	NE	NE	NE
COMMUNITY AND INSTITUTION															
62.	OAFEM	+	High	+	High	NE	NE	NE	NE	NE	NE	NE	NE	+	High
63.	EPAS	NE	NE	?	High	NE	NE	NE	NE	+	Low	NE	NE	+	High

##### Summarise the quality of PROMS and grading the evidence

All analyses were performed based on recommendations proposed in the COSMIN guideline. We compared the overall results based on predefined criteria to determine if the measurement property for each instrument is rated as sufficient (+) or insufficient (−), or inconsistent (±).

To conclude, the quality of the instruments and the results of all available studies for each measurement property were assessed for consistency. For consistent results, all studies should be summarised qualitatively or pooled quantitatively by performing a meta‐analysis, provided adequate quantitative data on psychometric properties exists. For instance, Intraclass Correlation Coefficients (ICCs) from different studies assessing the same measurement instrument can be pooled by calculating the weighted means and 95% confidence interval based on the sample size in each study. Instead of performing a meta‐analysis for this review, we opted for a qualitative summary of findings for several compelling reasons. A primary concern was the limited data availability on each psychometric property per instrument, making it impractical to quantitatively pool results meaningfully. For most instruments, data were only available from one or two studies.

Additionally, the included studies encompassed diverse cultural and linguistic contexts, complicating the aggregation of results. Furthermore, some included studies exhibited notable diversity in the operational definition of the primary outcome measure, and variations in the scales or response options employed for the same instrument precluded direct quantitative comparisons. This diversity often stemmed from modifications of the original instruments, including adding or reducing items used to measure abuse. There were also divergent assessment timeframes across studies per instrument (e.g., past month, past year). Considering these factors collectively, a qualitative summary best serves the purpose of this review, offering a comprehensive and nuanced understanding of the available evidence.

For inconsistent results, we further explored the reasons behind this inconsistency, including examining the populations, methods used, or quality of the studies. We summarised the consistent results based on these subpopulations. If there was no explanation for the inconsistent findings, the overall rating is inconsistent (±). For example, the overall rating for a particular measurement property, such as reliability, may be rated as sufficient (+) for measuring abuse among community‐based older persons but insufficient (−) in institutionalised older people. In cases where inconsistent results cannot be explained, the overall rating is determined based on the majority outcome of the results. Specifically, if at least 75% of the studies have consistent results, the evidence is rated as sufficient; otherwise, we downgraded the evidence to insufficient (Prinsen et al., 2018).

The quality of the evidence was graded using the modified GRADE approach into four levels of evidence: high, moderate, low, and very low (Holger et al., 2013). The quality of evidence of the content validity was graded based on three factors: risk of bias, inconsistency and indirectness. Other psychometric properties were evaluated based on four factors: risk of bias, inconsistency, imprecision, and indirectness. The overall rating and evidence quality of each study is reported in Tables 5 and 8. The summary of the overall rating and evidence of the quality of each instrument is shown in the Summary of Findings Table 1.

Two reviewers summarised and assessed the certainty of the overall evidence by reaching a consensus among the two reviewers. A third reviewer or the rest of the team members were consulted if there were any discrepancies.

##### Recommendations for instrument based on overall evidence and reporting of review

Finally, we summarised the findings in Summary of Findings Table 1, which we used to provide recommendations for selecting the most appropriate measurement instrument for a particular context.

## 6 RESULTS

### 6.1 Description of studies

#### 6.1.1 Results of the search

The search generated 15,200 hits. Three thousand eight hundred seventy‐five duplicate studies were removed, and 11,325 studies remained for title and abstract screening. We identified 1056 studies for full‐text review in the title and abstract screening. We found 114 studies that reported data from 68 AOP measurement instruments eligible for inclusion. Three studies reported on two instruments (Badenes‐Ribera et al., 2021; Buri et al., 2009; Conrad et al., 2009) and one reported on three instruments (Fang & Yan, 2021). The included studies were published between 1986 and 2023.

Table 2 lists all included studies and measurement instruments in this review. The study selection is presented in the PRISMA flowchart Figure 1.

#### 6.1.2 Included studies

This review included 87 studies reporting on 46 original instruments and 29 studies reporting on 22 modified versions of an original instrument (Table 2 and Supporting Information: Appendix 5). For example, the VASS‐12 items were included as an original measure, but modified versions such as the VASS‐10 items, VASS‐16 (Chinese), and VASS‐15 (Chinese) were also included, as reported in studies. All versions of the CTS instruments were modified, and the original version was not used to measure AOP.

Ninety‐seven studies (56 instruments) evaluated AOP in the community, 14 studies (10 instruments) in institutions, and four studies (2 instruments) in both the community and institution settings (Table 2 and Supporting Information: Appendix 6). Sixty‐nine studies (44 instruments) were conducted in high‐income countries (Table 2 and Supporting Information: Appendix 7).

One hundred thirteen studies (67 instruments) evaluated AOP measurement instruments targeted at older adults over 60, and only one study (Instrument: DVSQ) targeted women aged 50–79 with an average age of 63 years (SD 6.35, range 51–79). This instrument measures domestic violence against women.

Ninety‐five studies (51 instruments) reported multiple forms of abuse, with subscales ranging from 2 to 13. Four studies (4 instruments) measured overall abuse and neglect among older adults. Fourteen studies (11 instruments) focused on one specific type of abuse; one instrument assessed physical abuse (DVSQ), four instruments assessed psychological abuse (OAPAM, EPAS, CPEAB, EAVQ), two instruments assessed financial abuse (OAFEM, FIVE), three instruments assessed neglect (mGMS (Neglect), *mNSEAN (Neglect), mOARS‐ADL (Neglect)) and one instrument assessed verbal abuse (mCTS‐12 items (Verbal)). However, two studies (2 instruments) provided no information on the subscale measured (EAS and mVASS‐10 items).

The definition of AOP used in this review is based on the World Health Organization (WHO), which includes five subtypes of abuse: physical, psychological, sexual, financial and neglect. We found 28 studies (18 instruments) measured all five subtypes of abuse according to the WHO definition (mCTS‐34 items, mCTS‐M 38 items, m‐HS‐EAST, GMS, *mRAAL‐27 items, DEAQ, UKNPS, *mNSEAN, ABUEL, ATDEA, FVOW, *EAQ (Nepal), USCOACS, *EAQ (Iran), *EANQ (Iran), m‐EAQ, m‐EASI and QEEA) (Table 2 and Supporting Information: Appendix 8). Four studies (4 instruments) reported overall AOP (FPS‐2 items, mFPS‐5 items, *DVE (Thailand), *EAN (Puerto Ricans). The abuse subscales reported by 11 studies across three instruments did not align with those defined by the World Health Organization (WHO). For example, VASS‐12 items measured vulnerability, dependence, dejection, and coercion. Two studies (2 instruments) did not state the measured dimension or subtypes of abuse (m‐VASS‐10 items, EAS)—however, the study's author reporting on m‐VASS‐10 items listed the questions in the instrument.

The included studies assessed AOP from three perspectives: older adults, their caregivers and third‐party observers. Ninety studies directly administered the instrument to older adults, while 57 studies relied on proxy reports (29 from caregivers and 28 from third‐party individuals or staff members) (Table 2 and Supporting Information: Appendix 9). The remaining studies administered the instruments to a combination of two out of three respondent groups: older adults and caregivers, older adults and third‐party observers, or caregivers and third‐party observers.

#### 6.1.3 Excluded studies

Of the 482 full texts retrieved, 368 studies were excluded for various reasons, as shown in Figure 1. Exclusion criteria encompassed the use of the instrument for screening and diagnostic purposes, studies conducted in a hospital setting, and studies that did not report any psychometric properties.

### 6.2 Risk of bias in included studies

The methodological quality assessment of the studies evaluating the psychometric properties is shown in Tables 3 and 6.

The methodological quality assessment of studies reporting on instrument development and content validity is predominantly rated as doubtful or inadequate. Only one study was rated as very good (Ramirez et al., 2013), and two were rated as very good for content validity and adequate for instrument development (McCreadie et al., 2006; O'Keeffe et al., 2007).

Various reasons contribute to the identified risk of bias in instrument development and content validity. One primary factor is the inappropriate methodological approach employed in the studies. In these cases, authors failed to adhere to the appropriate qualitative design for instrument design, including concept elicitation and cognitive testing, or to conduct pilot studies with target populations. Similarly, for content validity, most authors did not employ an appropriate study design for data collection. Another issue is reporting bias, where authors neglected to provide sufficient data collection and analysis details.

For other psychometric properties, the risk of bias is a mix of very good, doubtful, and inadequate. Most of the studies have doubtful and inadequate risk of bias due to inappropriate methodology used for data analysis and reporting bias.

### 6.3 Synthesis of results

 

### 6.4 Description of measures

The instruments with the most evidence available in terms of studies reporting on the instrument development and the evaluation of the content validity in all domains (relevance, comprehensiveness, and comprehensibility) are DEAQ, OAPAM, *RAAL‐31 items, *ICNH (Norwegian), and OAFEM (Tables 3 and 4).

The instruments with the most evidence available in terms of the number of studies evaluating and reporting on the psychometric properties of the instruments are the HS‐EAST (11 studies across 5 of 9 psychometric properties), CASE (9 studies across 6 of 9 psychometric properties), VASS‐12 items (9 studies across 5 of 9 psychometric properties) and GMS (5 studies across 4 of 9 psychometric properties) (Tables 6 and 7).

#### 6.4.1 Primary outcomes: Overview of measurement properties

Instrument development was reported in 21 studies (20 instruments), while content validity was evaluated in 34 studies (25 instruments). Among the included studies, internal consistency was the most assessed psychometric property (89 studies, 56 instruments), followed by structural validity (42 studies, 28 instruments) and hypothesis testing/construct validity (32 studies, 20 instruments). Relatively fewer instruments evaluated criterion validity (5 studies, 4 instruments), measurement invariance (2 studies, 2 instruments) and cross‐cultural validity (one study, one instrument). Measurement error and responsiveness were not evaluated in any of the studies.

The results below are structured using the COSMIN taxonomy of measurement properties grouped into the domains of validity and reliability. Tables 3, 4, 6 and 7 detailed reports on the methodological quality and rating of psychometric properties in each included study. Tables 5 and 8 show the overall quality of evidence of content validity and other psychometric properties evaluated for each instrument.

##### Validity

###### Instrument development

Twenty‐one studies reported instrument development for 20 different instruments (OAPAM, UKNPS, DEAQ, GMS, MEAS, HS‐EAST, IEAQ‐Long form, CASE, FIVE, * IPPA (India), EMM, FVOW, EACS, R‐REM, *RAAL‐31 items, *ICNH (Norwegian), CHCS, DVSQ, OAFEM, EPAS). Instrument development was evaluated for relevance, comprehensiveness and comprehensibility.

Five studies (6 instruments) were evaluated for the methodological quality of all three domains in relation to their development (relevance, comprehensiveness and comprehensibility); R‐REM was ‘very good’, followed by UKNPS, OAPAM and OAFEM with ‘adequate’ and *RAAL‐31 items and *ICNH (Norwegian) with ‘doubtful’ quality. The rest of the instrument development studies evaluated only one or two of the domains, and the methodological quality ranged from ‘adequate’ to ‘inadequate’ (Table 3).

The UKNPS and DEAQ were rated as ‘sufficient’ for relevance, comprehensiveness, and comprehensibility, with the overall quality of evidence being moderate to low, respectively. The GMS was rated ‘sufficient’ for its relevance and comprehensibility with moderate quality of evidence, while comprehensiveness was not reported. The rest of the instruments were rated as ‘indeterminate’ or ‘insufficient’ (Table 4).

Six instruments (UKNPS, EMM, FVOW, EACS, CHCS, and R‐REM) were evaluated only for the quality of the development study, as their content validity was not reported in any studies.

###### Content validity

####### Overall quality of evidence of instrument development and content validity

The overall quality of evidence of the instrument development and content validity of each instrument was assessed for 16 instruments: rated ‘sufficient’ for OAPAM, GMS, UKNPS, DEAQ, R‐REM, and OAFEM (moderate quality of evidence); HS‐EAST, VASS‐12 items, *RAAL‐31 items and*mRAAL‐28 items (low quality of evidence). ‘Insufficient’ ratings were given to IEAQ‐Long form, mCTS‐34 items, and ATDEA with low quality of evidence. CASE, *NAS (Ghana) and *ICNH (Norwegian) were rated ‘inconsistent’, and no overall quality was given.

Tables 3, 4, 5 show each domain's content validity rating, the methodological quality of each instrument's development and content validity studies, and the overall methodological and content validity quality assessment of development and content validity studies per instrument.

###### Rating of content validity

Thirty‐four studies across 25 instruments reported content validity: CASE, GMS, DEAQ, OAPAM, mCTS‐34 items, VASS‐12items, HS‐EAST, IEAQ‐Long form, *NAS (Ghana), ATDEA, mGMS (Neglect), FIVE, EAS, *EA (Korea), MEAS, *IPPA (India), *mRAAL‐27 items, *RAAL‐31 items, *mRAAL‐28 items, *ICNH (Norwegian), *EAS (Korea), CPEAB, DVSQ, OAFEM and EPAS.

Twenty‐two studies across 14 instruments had all domains of the content validity evaluated (relevance, comprehensiveness and comprehensibility): GMS, DEAQ, CASE, OAPAM, VASS‐12 items, HS‐EAST, *NAS (Ghana), mCTS‐34 items, IEAQ‐Long form, ATDEA, *RAAL‐31 items, *mRAAL‐28 items, *ICNH (Norwegian) and OAFEM.

Eight instruments were rated ‘sufficient’, three with moderate‐quality evidence (GMS, two studies; OAPAM, two studies; and OAFEM, one study) and five with low quality‐evidence (DEAQ, one study; HS‐EAST, five studies; VASS‐12 items, two studies; *RAAL‐31 items, two studies; and *mRAAL‐28 items, one study). Three instruments were rated ‘insufficient’ with low‐quality evidence (IEAQ‐Long Form: one study; mCTS‐34 items: two studies; and ATDEA: one study). Three instruments were not rated due to inconsistency rating (CASE: one study, *NAS (Ghana): one study and *ICNH (Norwegian): one study).

Eleven studies (10 instruments) had the relevance and comprehensiveness evaluated but not comprehensibility: mGMS (Neglect), FIVE, EAS, *EA (Korea), MEAS, *IPPA (India), *EAS (Korea), CPEAB, DVSQ and EPAS. The mGMS (Neglect) (one study) and EPAS (one study), in which both relevance and comprehensiveness were rated ‘sufficient’ with moderate study quality.

One study focusing on *mRAAL‐27 items had only comprehensibility assessed, was rated as ‘sufficient’, and was deemed a low‐quality study.

###### Methodological quality of the studies evaluating each domain

Relevance was investigated in 33 studies across 24 instruments. Thirteen studies (12 instruments) involving both older adults and professionals (DEAQ, CASE, VASS‐12 items, HS‐EAST, *NAS (Ghana), mCTS‐34 items, IEAQ‐Long form, MEAS, OAPAM, *RAAL‐31 items, OAFEM and EPAS). Sixteen studies (13 instruments) involved only professionals (GMS, VASS‐12 items, HS‐EAST, mGMS (Neglect), FIVE, EAS, *EA (Korea), *IPPA (India), OAPAM, *EAS (Korea), CPEAB, DVSQ and EPAS). Four studies (4 instruments) focused solely on older adults (mCTS‐34 items, ATDEA, *mRAAL‐28 items, *ICNH (Norwegian)).

One study received a rating of ‘very good’ (Heravi‐Karimooi, 2010; DEAQ), and four studies received a rating of ‘adequate’ (Reichenheim et al., 2008: HS‐EAST; Zawisza et al., 2020: mGMS (Neglect); Conrad et al., 2009: OAPAM and OAFEM; Wang et al., 2007: EPAS). Twenty‐five studies were rated ‘doubtful’, and three were rated ‘inadequate’.

Comprehensiveness was assessed in 30 studies across 24 instruments. Fourteen studies (12 instruments) involving both older adults and professionals (DEAQ, OAPAM, CASE, VASS‐12 items, HS‐EAST, *NAS (Ghana), mCTS‐34 items, IEAQ‐Long form, MEAS, *RAAL‐31 items, OAFEM, EPAS). Three studies (three instruments) focused on older adults only (ATDEA, *mRAAL‐28 items, *ICNH (Norwegian)). Fourteen studies (12 instruments) involved only professionals (GMS, VASS‐12 items, HS‐EAST, mGMS (Neglect), FIVE, EAS, *EA (Korea), *IPPA (India), OAPAM, *EAS (Korea), CPEAB and DVSQ)).

One study was rated ‘very good’ (Heravi‐Karimooi, 2010; DEAQ), and four studies were rated ‘adequate’ (Reichenheim et al., 2008: HS‐EAST; Zawisza et al., 2020: mGMS (Neglect); Conrad et al., 2009: OAPAM and OAFEM; Wang et al., 2007: EPAS). Twenty‐one studies were rated ‘doubtful’, and four were rated ‘inadequate’.

Comprehensibility was assessed in 17 studies across 15 instruments involving the target population (GMS, DEAQ, OAPAM, CASE, VASS‐12 items, HS‐EAST, *NAS (Ghana), mCTS‐34 items, IEAQ‐Long form, ATDEA, *mRAAL‐27 items, *RAAL‐31 items, *mRAAL‐28 items, *ICNH (Norwegian), OAFEM).

One study received a rating of ‘very good’ (Heravi‐Karimooi, 2010: DEAQ), three studies were rated ‘adequate’ (Giraldo‐Rodríguez, 2013: GMS; Paixão et al., 2007: CASE; Conrad et al., 2009; OAPAM and OAFEM), two studies were rated ‘inadequate’ (Jervis et al., 2014, Ozmete, 2017: HS‐EAST) and the rest were rated as ‘doubtful’.

###### Structural validity

Structural validity was tested for 28 instruments in 43 studies. The methodological quality for the majority of studies testing structural validity was ‘very good’ (35 studies), with one study each with ‘adequate’ and ‘doubtful’ rating and five studies with ‘inadequate’ methodological quality. Studies were rated as doubtful or inadequate if they had important flaws in the design or statistical methods (Table 6).

The quality rating of psychometric properties for 27 studies was ‘sufficient’. Six studies were rated ‘insufficient’, and 10 were ‘indeterminate’. A sufficient rating was received if the results of the Confirmatory or Exploratory Factor Analysis or Rasch/Item Response Theory were above the Model fit cut‐off set (above 0.7) based on COSMIN criteria. Insufficient ratings were given when the results did not meet sufficient criteria.

Overall ratings of 19 instruments were ‘sufficient’ with overall high‐quality evidence of 17 instruments (OAPAM, OAFEM, mCTS‐M 38 items, VASS‐12 items, mVASS‐15 items, mGMS (Neglect), DEAQ, R‐REM, *EAS (Korea), FVS, FIVE, MEAS, *NAS (Ghana), *IPPA (India), EACS, Native EAS‐Short Form and IEAQ‐Long form). One instrument had moderate‐quality evidence (CASE), and another had very low‐quality evidence (EMS). Three instruments were rated ‘insufficient’ (EVEQ, FVOW and *ICNH (Norwegian)). Four instruments received an ‘indeterminate’ overall rating (WHRS, EMM, *EAN (Puerto Ricans) and *IPVQ). In comparison, the two instruments had ‘inconsistent’ overall ratings due to varying ratings of the individual studies evaluating the instrument (HS‐EAST and GMS).

###### Cross‐cultural validity

Only one study focusing on one instrument (CASE) assessed its cross‐cultural validity among the Urdu language‐speaking population in India. CASE was rated ‘sufficient’ with a ‘very good’ quality of the study methodology. Overall, the quality of evidence for this instrument was assessed as moderate.

Studies were considered sufficient if no significant differences were found between group factors (such as age, gender, language) in multiple group factor analysis or if there were no significant instances of Differential Item Functioning for group factors. Sample characteristics, sample size, and the analysis approach were important factors determining the methodological quality of studies.

###### Measurement invariance

Two studies, one on the instrument CASE and the other on *mNSEAN (Neglect), were rated as ‘sufficient’ with a ‘very good’ quality of study methodology for measurement invariance. Overall, the quality of the instruments' evidence was assessed as high. The criteria COSMIN sets for a sufficient rating in this context are similar to those for cross‐cultural validity.

###### Criterion validity

Five studies across four instruments evaluated criterion validity. All studies were rated ‘very good’, ranging from ‘sufficient’ (2 studies) to ‘insufficient’ (3 studies). Studies were rated sufficient if the correlation with the gold standard was ≥0.70 or AUC ≥ 0.70 and considered insufficient if they did not meet one of these criteria.

One instrument (mCTS‐10 items) received an overall sufficient rating with high‐quality evidence. Two instruments (VASS‐12 items and FPS‐2 items) were rated as insufficient with high‐quality evidence. Additionally, one instrument (HS‐EAST) received an inconsistent rating.

###### Construct validity

Construct validity (convergent and discriminative or known‐groups validity) was evaluated in 32 studies across 20 instruments. Convergent validity or comparison with other outcome measurement instruments was evaluated in 28 studies, and discriminative or known‐groups validity or comparison between subgroups was assessed in 10 studies. Out of 32 studies, only six across four instruments (mCTS‐M 38 items, HS‐EAST, CASE and mGMS (Neglect)) assessed both convergent and discriminative or known‐groups validity.

Convergent validities were conducted using the Geriatric Depression Scale, Anxiety and Stress Scale, Activity of Daily Living Scale or Quality of Life Score. Known‐group validity was conducted among abused and non‐abused groups.

The quality of studies evaluating convergent validity was rated as ‘very good’ (26 studies), while one study received ratings of ‘adequate’ and ‘inadequate’, respectively. For discriminative or known‐groups validity, eight studies were rated as ‘very good’, with one study each receiving ratings of ‘adequate’ and ‘inadequate’.

Thirty studies were rated as ‘sufficient’ and two ‘indeterminate’. Studies were rated sufficient if the result was in accordance with the hypothesis and indeterminate if no hypothesis was defined.

Each instrument's overall construct validity ratings were ‘sufficient’ for 18 instruments. Sixteen instruments with high‐quality evidence (mCTS‐34 items, mCTS‐C 25 Items, VASS‐12 items, HS‐EAST, CASE, mGMS (Neglect), OAPAM, *mNSEAN, OAFEM, mOARS‐ADL(Neglect), Native EAS‐Short Form, EPAS, FIVE, MEAS, *NAS (Ghana), and EACS) and one each with moderate (GMS) and very low (mCTS‐M 38 items) quality of evidence. An overall ‘indeterminate’ rating was given for two instruments, each with high (FVOW) and moderate (mFPS‐5 items) overall quality of evidence.

##### Reliability

###### Internal consistency

Internal consistency was measured for 57 instruments in 93 studies. The methodological quality of studies testing internal consistency was rated ‘very good’ for 57 studies, ‘inadequate’ for 35 studies and ‘doubtful’ for one study. Studies were rated ‘insufficient’ if the value for internal consistency statistic was not calculated for each unidimensional (sub)scale separately.

The internal consistency for 36 studies was rated as ‘sufficient’, five as ‘insufficient’, and 52 studies were rated as ‘indeterminate’. A sufficient rating was assigned if there was at least low evidence for sufficient structural validity and Cronbach's alpha(s) ≥ 0.70 for each unidimensional scale subscale. Insufficient ratings were given when there was low evidence for sufficient structural validity and Cronbach's alpha(s) < 0.70 for each unidimensional scale or subscale.

The overall rating of 19 instruments was assessed as ‘sufficient’, with 13 instruments receiving an overall high quality of evidence (mCTS‐M 38 items, mVASS‐15 items, mGMS (Neglect), OAPAM, *ICNH (Norwegian), DEAQ, R‐REM, OAFEM, EVEQ, *NAS (Ghana), *EAN (Puerto Ricans), EACS, Native EAS‐Short Form). Two instruments had moderate‐quality evidence (VASS‐12 items and CASE), while one had low‐quality evidence (*EAS (Korea)). Three instruments were rated with very low overall quality of evidence (FVOW, FVS and *IPPA (India)).

Four instruments were rated ‘insufficient’ (EMM, FIVE, MEAS and IEAQ‐Long form) with high‐quality evidence, and 34 instruments' overall ratings were ‘indeterminate’. Most studies (30) received an ‘indeterminate’ rating as they did not meet the criteria for at least low evidence of sufficient structural validity.

###### Reliability

Eighteen studies investigated the reliability of 11 instruments. The methodological quality of studies testing for reliability was mainly ‘inadequate’ (14 studies), followed by ‘adequate’ (2 studies), ‘very good’ (one study) and ‘doubtful’ (one study). The quality of reliability within these studies was rated ‘indeterminate’ (11 studies), ‘sufficient’ (6 studies) and ‘insufficient’ (one study). A sufficient rating was given if ICC or weighted Kappa ≥ 0.70 and indeterminate if ICC or weighted Kappa is not reported. Most of the studies' methodological quality was rated inadequate due to inappropriate statistical methods, lack of reporting on study design and inadequate time interval before retesting.

Overall, three of the 11 instruments were rated ‘sufficient’. The quality of evidence varied, with one instrument (EPAS) being high while two instruments received very low ratings (GMS and *EAQ (China)). Another instrument (CPEAB) was deemed ‘insufficient’ and had a very low quality of evidence. Six instruments were rated ‘indeterminate’ (HS‐EAST, DEAQ, VASS‐12 items, mGMS (Neglect), EAS and *EANQ (Iran)), and one instrument (CASE) received an ‘inconsistent’ rating due to varying study methodological quality.

##### Measurement error

None of the studies evaluated this psychometric property for the included instruments in this review.

##### Responsiveness

None of the studies evaluated this psychometric property for the included instruments in this review.

Tables 6, 7, 8 show the methodological quality of other psychometric properties studies, the quality of other psychometric properties, and the overall methodological and quality assessment of other psychometric properties studies per instrument.

#### 6.4.2 Secondary outcome: Definition of AOP and its operationalisation

Various definitions of AOP were employed in the instruments analysed. The most commonly referenced definitions are based on The Toronto Declaration on the Global Prevention of Elder Abuse, Geneva: World Health Organization (World Health Organization, 2002). Some authors also cited established organisations' definitions, including the Centres for Disease Control and Prevention, The American Medical Association (AMA) and the National Research Council (United States) Panel to Review Risk and Prevalence of Elder Abuse and Neglect (Hall et al., 2016; National Research Council Panel to Review Risk Prevalence of Elder Abuse and Neglect, 2003; Yawn et al., 1992).

All four commonly cited definitions above share similar core concepts, characterising AOP actions as causing harm or creating a serious risk of harm, failing to provide for basic needs or protecting vulnerable older adults from harm, whether through intentional or unintentional actions by a caregiver or other individuals in a position of trust.

The reviewed studies identified a range of measurement approaches. Seventy‐four studies employed binary questions, 20 used the Likert scale, and 15 used frequency counts. Ten studies did not report on the range of measures used. Out of these studies, 43 asked about the frequency of abuse, and 24 included inquiries about potential perpetrators.

## 7 DISCUSSION

### 7.1 Summary of main results

We systematically reviewed the evidence for the psychometric properties of instruments used to measure the prevalence of AOP in community and institutional settings from the inception of electronic databases to May 2023. We thoroughly assessed the quality of the included studies, rigorously evaluated the psychometric properties of the measurement instruments, and described the practical applications of each instrument to provide evidence‐based recommendations for determining which AOP measurement instruments to use in different settings. The summary of each instrument and critical findings are presented in the Summary of Findings Table 1.

Our study builds upon prior research conducted by Cooper, Manela, et al. (2008), De Donder et al. (2011) and Sooryanarayana et al. (2013), who collectively underscored the imperative of employing a valid and reliable measurement instrument when assessing AOP (Cooper, Manela, et al., 2008; De Donder et al., 2011; Sooryanarayana et al., 2013). While their studies provide valuable insights into the existing landscape of measurement instruments for AOP, their work may benefit from a more detailed assessment of various aspects of validity and reliability. This observation underscores the potential for further research to pinpoint specific focus areas in refining AOP measurement instruments.

Our search and screening process identified 114 studies that described 68 instruments used to measure AOP. Forty‐six original instruments and 22 modified versions of an original instrument are included in this review. The modified versions were included in the review as independent instruments, as comparing psychometric properties across the original and modified versions would be inaccurate. The modified versions may have different numbers of items, constructs of abuse measured and study settings.

#### 7.1.1 Instrument development

Best practice guidance suggests instrument development should involve concept elicitation and testing a new instrument using cognitive interviews or pilot studies (Terwee et al., 2018). This review found that 20 instruments have reports on their development available. Among them, 14 instruments assessed at least one aspect of content validity, while six solely presented information on the instrument's development. Notably, studies detailing the development of the R‐REM instrument demonstrated very good methodological quality. This was evident in the comprehensive description of the qualitative methodology and cognitive/pilot study (Ramirez et al., 2013).

The paucity of current evidence regarding instrument development may stem from two potential factors: suboptimal development practices or the absence of established reporting guidelines. In most reported studies, detailed reporting on instrument development was lacking. Some instruments were initially developed to measure the prevalence of other forms of interpersonal violence and were subsequently adapted for AOP assessments. Among the studies that provided information on instrument development, there was generally limited information regarding the qualitative approach, and the conduct of cognitive interviews or pilot studies was often omitted. Like content validity, the instrument development process requires substantial resources and input from various stakeholders, including professionals/experts and the target population. In most cases, the authors briefly mentioned that the instruments were derived from a combination of literature reviews and input from expert panels.

#### 7.1.2 Content validity

This review identified 34 studies across 25 instruments that evaluated the instrument's content validity. Twenty‐two studies covering 14 instruments evaluated all aspects of content validity. Thirty‐two studies (24 instruments) evaluated relevance and comprehensiveness. Seventeen studies (15 instruments) evaluated comprehensibility. Only three modified versions (mCTS‐34 items, m‐RAAL‐28 items and m‐RAAL‐27 items) had at least one of the aspects of content validity assessed. Most AOP measurement instruments have not undergone comprehensive content validity evaluations. This could be attributed to the resource‐intensive and time‐consuming nature of the research process required to conduct such assessments, a consideration that authors may overlook if they have used or adapted an existing instrument. There is a possibility that the reporting of content validity was not performed thoroughly or exhaustively.

Additionally, some authors may presume that widely used or adapted instruments already have their content validity established in prior studies, thus not deeming it necessary to re‐evaluate them. Resource limitations, including restricted funding or lack of access to specialised expertise in content validation methodologies, may further dissuade authors from undertaking a thorough content validity assessment. In addition, the lack of clear and standardised guidelines for appraising content validity in the context of AOP measurement instruments may contribute to the observed scarcity of assessments in the existing literature.

Among the studies reporting all aspects of the instrument's content validity, eight instruments were rated as sufficient, and the quality of evidence ranged from low (4 instruments) to moderate (4 instruments). Four were rated as insufficient, and three instruments were not rated due to inconsistencies in information. Many studies fell short in adhering to the comprehensive reporting standards as required in the COSMIN checklist. Crucial details regarding the qualitative approach, such as specifics about the interviewer(s), utilisation of a topic guide, procedures for data collection (including audio‐recording and verbatim transcribing) and the method of data analysis, were omitted. This lack of thorough reporting likely contributed to the varying ratings of content validity across the assessed instruments.

Furthermore, inconsistencies in reporting across studies resulted in three instruments being ineligible for rating. These discrepancies highlight the need for standardised reporting guidelines in assessing content validity for AOP measurement instruments. This process would enhance transparency in research practices and facilitate more accurate and reliable evaluations of these instruments.

#### 7.1.3 Considerations relating to other psychometric properties

The validity and reliability of an instrument require the measurement of other psychometric properties listed in the COSMIN guideline (Mokkink et al., 2018; Prinsen et al., 2018). These include properties assessing the validity of the instrument (structural validity, cross‐cultural validity, measurement invariance, criterion validity and construct validity), the reliability of the instrument (internal consistency, reliability (test–retest), measurement error) and responsiveness of the instrument.

We found that the most common psychometric properties evaluated by the researchers were internal consistency, followed by structural validity. Internal consistency emerged as a common property because many prevalence studies reported Cronbach's alpha value of the instrument used in the study. However, it is important to note that the primary aim of these studies was to measure prevalence. According to the COSMIN guidelines, the quality of structural validity should be considered before determining the quality of internal consistency evidence (Mokkink et al., 2018; Prinsen et al., 2018). In instances where structural validity was not adequately demonstrated, the rating and quality of evidence for internal consistency were appropriately downgraded. As a result, 30 instruments had their internal consistency study quality rated as indeterminate due to the criterion that there be at least low evidence for sufficient structural validity. It was observed that a significant number of studies in this review reported a Cronbach's alpha based on scales that were not strictly unidimensional despite evaluating multiple subscales of abuse. The risk of bias in these studies was rated as inadequate (Mokkink et al., 2018). This practice introduces a potential source of bias, as it may not accurately capture the true internal consistency of the instrument. Based on the COSMIN guidelines, this raises concerns about the adequacy of the internal consistency assessment (Mokkink et al., 2018).

Some studies evaluated structural validity, most of whose methodological quality was very good; however, only 40% of the included studies reported on structural validity. Assessment of the structural validity of an instrument requires an adequate number of participants, and the statistical analysis of the structural validity using confirmatory or explorative factor analysis needs input from an expert in this field and is not commonly conducted by many researchers. It may also be challenging to gather a sufficiently large sample size (often a few hundred) to conduct a robust factor analysis, especially for vulnerable populations.

The included studies evaluated two aspects of the construct validity: comparison with other outcome measurement instruments (convergent validity) and comparison between subgroups (discriminative or known‐groups validity). While assessing convergent validity, studies often aimed to establish associations between AOP and adverse outcomes or other risk factors. For example, convergent validity of the instrument was reported in studies evaluating the relationship between AOP and mental health issues such as depression and anxiety, quality of life, caregiver burden and social support. Studies also evaluated the validity of discriminative or known groups, such as between genders, different respondent types and abused versus non‐abused groups. Some studies also examined convergent validity with other AOP measurement instruments, such as HS‐EAST, CTS, and VASS‐12 items. Most of the studies found that the results were in accordance with their hypothesis, hence giving a sufficient rating for construct validity with high‐quality evidence. Evaluating known‐group validity is crucial for understanding the instrument's ability to capture nuanced differences in experiences of AOP. Furthermore, studies assessing convergent validity with other AOP measurement instruments contribute to understanding the degree of overlap or agreement between different instruments, providing insights into the robustness and consistency of measurements.

However, the studies included in this review did not consider some important risk factors, such as age group, gender, and socioeconomic status, while assessing the validity of the AOP measurement instrument. Therefore, it is crucial to consider these factors while determining the construct validity of the AOP measurement instrument. Convergent or discriminant validity can be used to evaluate these factors and to examine whether the scores obtained from the AOP measurement instrument align with relevant hypotheses, such as differences among demographic groups in socioeconomic status, age groups, and gender. This evaluation assumes that the AOP measurement instrument accurately measures the intended construct of AOP.

A few studies evaluating reliability (test–retest) were identified. However, the majority of these studies received inadequate methodological ratings. This deficiency primarily arose from the absence of an appropriate study design, erroneous statistical reporting, and an insufficient time interval for retest. Moreover, in some instances, there was a misapplication of statistical measures, with Kappa and ICCs being used interchangeably and inappropriately for the type of outcomes measured. Assessing reliability through test–retest measures presents its own set of challenges. This is due to the logistical difficulties associated with re‐administering the instrument to the same population within a defined time frame. This factor may have contributed to the limited reliability reporting in many studies. Other factors influencing the reliability evaluation could include participant variability, instrument sensitivity, and potential changes in the construct being measured over time. These aspects can further hinder the accurate reliability assessment in AOP measurement instruments when not adequately considered.

Other less commonly evaluated psychometric properties were cross‐cultural validity, measurement invariance and criterion validity. The assessment of criterion validity often necessitates researchers to employ two distinct instruments for a single construct, frequently involving a comparison with professional clinical judgement. Evaluating cross‐cultural validity can be complex due to various factors, including the need for expertise in diverse cultural contexts, potential language barriers, and the complexity of data analysis across different cultural groups. The scarcity of studies establishing cross‐cultural validation raises serious concerns, given the widespread use of specific AOP prevalence measurement instruments and the frequent comparisons of prevalence estimates across countries.

#### 7.1.4 Definition of AOP and its operationalisation

##### AOP definition

Most studies used the AOP definition based on The Toronto declaration on the global prevention of AOP, which the World Health Organization endorsed (World Health Organization, 2002).

The studies included in this review mainly evaluated instruments measuring at least one of the five subscales of AOP defined by WHO. Most studies included all five abuse subscales (18 instruments). However, there are also studies evaluating instruments measuring the AOP subscale, which is not included in the list provided by WHO (VASS‐12 items, *NAS (Ghana) and mCTS‐Verbal 12 items (Hall et al., 2016). An illustrative example is the original VASS‐12 items instrument, widely used to measure AOP prevalence. This instrument encompasses subscales that gauge vulnerability, dependence, dejection and coercion. It is essential to acknowledge that the diverse approaches to defining and measuring AOP in the literature and current research landscape inevitably introduce variability in the reported prevalence rates. Furthermore, variations in definitions and measurement frameworks may impact the identification of and response to AOP in different contexts and populations. This highlights the need for continued dialogue and consensus‐building to ensure a standardised and inclusive understanding of AOP for research and practice.

##### Threshold or score to define abuse

This review included studies that reported different thresholds for defining a case of AOP. The instruments included in this review exhibited diverse evaluation criteria, with distinctions in the subscale, items, response options, recall periods, and frequency and severity of abuse experienced by older adults.

Notably, the different instruments posed distinct sets of questions for each subscale. For example, instrument GMS has five items for physical abuse, and DEAQ has eight items, while for psychological abuse, EPAS has 32 items, and GMS has six items (Table 2). Additionally, while some instruments incorporated questions on frequency and severity to derive final scores, others did not follow this approach. Furthermore, response options across the included instruments were notably heterogeneous, predominantly adopting either a dichotomous (Yes/No) format or a Likert scale. These disparities, observed across the range of AOP instruments, undoubtedly contribute to inconsistencies in prevalence measurements, ultimately undermining the accuracy of AOP prevalence estimates (Burnes et al., 2017).

##### Dimensions of abuse measured

Most of the instruments in this review evaluated at least one of the five subscales outlined in the WHO abuse classifications. However, some instruments have been specifically tailored to assess one type of abuse. This is a critical consideration because relying solely on an instrument that assesses one dimension of abuse may not offer a comprehensive understanding of AOP. Current evidence indicates that many victims of AOP experience multiple forms of mistreatment simultaneously (Sooryanarayana et al., 2013, 2017; Yon et al., 2017; Yon, Ramiro‐Gonzalez, et al., 2019). Therefore, a narrow focus on a single type of abuse could lead to an underestimation of the prevalence and complexity of AOP. Instruments must incorporate a holistic approach, encompassing various dimensions of abuse, to provide a more accurate representation of the challenges faced by older adults.

#### 7.1.5 Other non‐psychometric properties issues and gaps related to AOP measurement instruments

##### Screening instruments used for prevalence measurement

Around 25% of the 114 studies in this review evaluated psychometric properties for one of three instruments: HS‐EAST (11 studies), VASS‐12 items and CASE (9 studies). All three instruments were evaluated extensively for content validity and five to six psychometric properties. However, the HS‐EAST and CASE instruments had studies reporting instrument development for the instrument design section but no information on cognitive interviews or pilot studies for instrument development evaluation. No studies have reported the development of the VASS‐12 items.

Interestingly, these instruments were originally developed for screening and other forms of interpersonal violence. However, researchers have used them to measure the prevalence of AOP upon modifications. The HS‐EAST was developed in 1986 through the collaboration of experts and a comprehensive literature search (Hwalek & Sengstock, 1986). The instrument was initially known as the Sengstock‐Hwalek Comprehensive Index of Elder Abuse and was used to identify abuse victims with 209 risk indicators (Hwalek & Sengstock, 1986). Risk indicators are divided into three major categories: the characteristics of the elderly victims, caretakers, and specific situations. VASS‐12 items were developed from the HS‐EAST by Hwalek and Sengstock (1986), the Conflict Tactics Scale (Schofield & Mishra, 2003). CASE is designed to screen for current physical, psychosocial or financial abuse and neglect of older adults by primary or other unpaid caregivers (Reis & Nahmiash, 1995).

The subscales measured by these instruments do not align with WHO classifications, except for CASE. The CASE is administered to caregivers to assess physical, psychosocial, financial and neglect. The HS‐EAST measures the following subscales: physical (direct abuse), violation of personal rights, characteristics of vulnerability and potentially abusive situation, and VASS‐12 items measure vulnerability, dependence, dejection, and coercion. Items in the HS‐EAST and VASS‐12 mainly assess the risk factors, except for the HS‐EAST, which has a subscale that measures physical abuse (direct abuse).

The CASE has been evaluated rigorously using these three instruments, covering six out of nine other psychometric properties, including internal consistency, cross‐cultural validity and measurement invariance, reliability, criterion validity and construct validity. The overall rating of the psychometric properties was sufficient, with the quality of evidence ranging from moderate to high and inconsistent ratings for reliability.

##### Translation process

Our review revealed a notable dearth of comprehensive information regarding the translation process of research instruments in some studies. This deficiency could be attributed to several plausible explanations. Firstly, the researchers may not be fully acquainted or thoroughly familiar with the recommended reporting practices for translation procedures. Secondly, restricted word limits in scholarly journal papers could have compelled researchers to curtail detailed accounts of the translation process. Lastly, there may exist instances where adherence to the established standard protocols for instrument translation was not rigorously maintained. Inadequate reporting on the translation process can substantially compromise the transparency and replicability of research, potentially introducing threats to the integrity, validity, and reliability of the measurements derived from the instrument. Furthermore, it may impede cross‐cultural comparisons and limit the generalisability and applicability of study findings.

### 7.2 Overall completeness and applicability of evidence

This review was conducted thoroughly and meticulously, encompassing aspects such as the breadth of the search, including all publication languages, double screening of all studies, a substantial number of screened and included studies, and applying the COSMIN 2018 criteria. The synthesis of instrument content and administration practicalities enhances the usefulness of this review by providing researchers with a means to identify the most suitable instrument. It also enables them to conduct methodologically appropriate studies to evaluate existing instruments, develop new instruments, or adapt instruments to their specific research needs.

Regarding the implications of the findings of this review on measuring AOP prevalence, several issues require careful consideration. Although we conducted an extensive search to find relevant studies in interdisciplinary databases and related subjects, there might still be some gaps that remain in the literature. It is plausible that we may have overlooked pertinent studies, particularly those that were not widely indexed. We might have missed some prevalence studies that briefly reported the psychometric properties evaluated as secondary outcomes of the study. Although we tried to include grey literature sources, obtaining scholastic grey literature (such as untranslated and unindexed reports) can be challenging.

Our review did not impose any restrictions on the language of the study publications. However, we need to highlight that the search terms were exclusively in English, which does not rule out the possibility of finding non‐English‐published research. Although we captured a few studies published in languages other than English, such as Chinese, Korean, Japanese, Persian, and Portuguese, future reviews could adopt a more deliberate approach to including non‐English search terms.

The heterogeneity of findings among these studies might be attributed to different methodologies, scoring of the outcome's measurement and inconsistent instrument adaptations. In this review, we did not evaluate the methodological quality of the instrument's translation process from its original language. In general, some studies have not extensively reported the translation process. Most of the primary aims of the studies included in this review were not to measure psychometric properties. The authors performed a psychometric properties analysis based on the data collected for the primary outcome, which could be a prevalence measurement or studies assessing the factors associated with AOP. Hence, the explicit methodological quality of individual studies, overall rating of psychometric properties, and quality of evidence of AOP measurement instruments are still concerns.

A summary of the instruments included in this study is shown in Table 2. We summarise the subscales measured, the response option, recall period, frequency, severity, language and translation, setting and respondents of each study, which may be necessary for individual researchers depending on the aims and objectives of each study. We also summarise the practical administrative elements of each measure, such as the number of items of the instruments, length of administration and available translations. Beyond assessing the quality of psychometric properties, some instruments may be better suited for particular research undertakings.

In summary, the applicability of the evidence of this review to current practice in AOP measurement instruments is adequate to show that there is a lack of high‐quality studies evaluating the validity and reliability of the available instruments to measure prevalence in community and institutional settings. The authors of this review could not recommend a specific instrument for measuring the prevalence of AOP in community and institutional settings. Hence, future research should map the available items of the existing instruments, develop a comprehensive instrument assessing all AOP subscales, and evaluate the instruments' validity and reliability by adopting high methodological quality across countries.

### 7.3 Quality of the evidence

In this review, we employed the stringent 2018 COSMIN criteria to evaluate the psychometric properties of studies and instruments. These criteria are thorough and require significant time and effort, necessitating multiple evaluation steps by the research team at both individual study and instrument levels. The COSMIN guidelines for assessing the psychometric properties of measurement instruments are comprehensive and methodical. The instructions provided for the steps involved in evaluating the instrument's validity and reliability were extensive and meticulously detailed.

Despite this, we identified some limitations in the review process. The current iteration of the COSMIN guidelines can be time‐intensive to implement. Given the extensive number of studies and instruments in this review, we recognised the need for a more efficient approach to reporting and synthesising individual study quality, psychometric properties quality, and overall evidence for each instrument. While we utilised the COSMIN rating forms provided by the guidelines' authors, we also made some adaptations and modifications to these forms to facilitate systematic data summarisation. This approach allowed our team to offer explicit evaluations of each reported psychometric property for individual studies while upholding the comprehensiveness and integrity of the COSMIN guidelines.

Furthermore, our initial review indicated that utilising the ‘lowest rated items count’ approach to assess the methodological quality of studies might not reasonably account for minor methodological issues. We had to convene a consensus meeting with fellow team members to determine whether or not to comply with this rule. Nevertheless, reviewers assessing the methodological quality of studies should exercise appropriate discretion, as the primary goal of the COSMIN guidelines is to enable researchers to select the most suitable instruments for their intended measurements.

In the Summary of Findings Table 1 and Tables 5 and 8, we summarised the overall quality of the evidence of each study and the instrument's psychometric properties using the GRADE assessment. The GRADE assessment showed that the psychometric properties of the instruments included in this review were rated as moderate to very low‐quality evidence.

The evidence suggests that our confidence in the methodological quality of individual studies, overall rating, and quality of evidence of each instrument are limited, and the psychometric properties of existing AOP measurement instruments need to be better researched. A substantial number of the included studies were conducted independently, lacking thorough prior scrutiny of the instrument's psychometric properties. This was evident because certain psychometric properties were used across multiple studies without modifying the instruments, study population and settings. The review findings imply that measuring the prevalence of AOP in community and institutional settings using existing instruments might not yield accurate and dependable data for conducting cross‐country comparisons.

We recommend that researchers utilise the information provided in the Summary of Findings Table 1 and Tables 3, 4, 5, 6, 7, 8 to examine all psychometric properties of the instruments, enabling them to assess the comprehensiveness of the evaluation of each property and the overall quality of these properties for each instrument. Moreover, when selecting a measurement instrument, it is crucial to consider that specific psychometric properties might be more relevant to particular research studies than others. The need for cross‐cultural validity may not be imperative when using a measurement instrument in the original context of its development. However, this could become critical when applying the same instrument in a different country or cultural setting. We also suggest that researchers employ an appropriate methodological approach when assessing the psychometric properties of instruments with doubtful and inadequate methodological quality. Likewise, researchers should investigate the instrument's psychometric properties, which have not been examined previously.

### 7.4 Potential biases in the review process

This review adhered to the published protocol (Mohd Mydin et al., 2023), and any variances from the published protocol are documented in the section that outlines discrepancies between the protocol and review. It is improbable that there were oversights in identifying studies for this review, as we conducted a thorough search across databases, websites, trial registries, and reference lists. Nonetheless, some factors could have potentially introduced bias into the review.

First, there is the possibility of bias in the review process, particularly during the screening or data extraction stages, despite our extensive efforts to minimise it. Second, we did not directly communicate with the authors to request missing information about study characteristics or to seek clarification on data. This decision was primarily due to the extensive number of included studies, and earlier, less well‐documented studies date back more than three decades. Third, we incorporated all studies that assessed AOP in community and institutional settings, regardless of whether the authors opted for an instrument that may not have been optimally designed. We observed that when the authors utilised an instrument not explicitly tailored for measuring AOP prevalence, the resulting psychometric properties tended to be suboptimal. Fourth, we omitted studies that employed AOP measurement instruments in community and institutional settings but did not provide information on psychometric properties. It is possible that we overlooked these instruments, which is why they were not included in our list of prevalence measurement instruments. Fifth, most of the included studies focused on respondents who were mentally well, and only a few included those with neurodegenerative issues, such as cognitive impairment, which is common among older adults.

Consequently, the psychometric properties may not entirely reflect the validity and reliability of the instruments when applied to this population. Finally, it is crucial to acknowledge that AOP measurement instruments face limitations due to variations in the recall period among studies and instruments. These measures typically assess exposure to abuse over different timeframes, ranging from as recent as 2 weeks ago to 12 months ago or since the individual turned 60. This diversity in timeframes is susceptible to recall bias, as respondents may experience memory decline or distortion, potentially influencing the accuracy of their responses.

### 7.5 Agreements and disagreements with other studies or reviews

This is the first review evaluating the psychometric properties of the AOP measurement instrument in the community and institutional settings using the COSMIN checklist. A few reviews have been conducted on other types of interpersonal violence, such as child abuse, cyber dating and domestic violence (Georgieva et al., 2021, 2023; Meinck et al., 2023; Ravi et al., 2023; Saini et al., 2019; Soto & Ibabe, 2022; Steele et al., 2024; Tarriño‐Concejero et al., 2023; Yoon et al., 2021a, 2021b). Our findings are consistent with reviews of the psychometric properties of other types of interpersonal violence, including emerging cyber‐dating violence.

Reviews on child abuse measurement instruments were conducted and published from two perspectives: adult retrospective self‐report instruments on child abuse and neglect and child and adolescent self‐report instruments (Meinck et al., 2023; Steele et al., 2024). Similarly, their findings showed that the quality of evidence for the instruments' psychometric properties ranged from ‘very low’ to ‘high’ for child and adolescent self‐report instruments and ‘low’ to ‘high’ for adult retrospective self‐report instruments (Meinck et al., 2023). A review of adult retrospective self‐report instruments on child abuse and neglect showed there are a few instruments that can be recommended with rigorous evidence; however, many instruments were not evaluated concerning their content validity (Steele et al., 2024; Yoon et al., 2021a, 2021b). The findings are consistent with this review, in which the instrument used widely to measure the prevalence of AOP in the community and institutions did have a content validity evaluation, and the quality of evidence ranged from ‘very low’ to ‘moderate’. We could not recommend any instruments based on our reviewed evidence, as most instruments were not robustly developed and evaluated for their psychometric properties.

A review of studies of culturally responsive domestic violence measurements found that the most commonly reported psychometric properties were content validity, criterion validity and construct validity (Ravi et al., 2023). Likewise, in this review, it was observed that most assessments regarding instrument reliability predominantly relied on internal consistency measures rather than test–retest methodologies. This preference may be due to logistical difficulties in gathering data from vulnerable individuals on multiple occasions. Similarly, content validity, structural validity and internal consistency were the most commonly reported psychometric properties of instruments measuring dating violence, and none reported measurement error and responsiveness (Tarriño‐Concejero et al., 2023). The content validities were poorly rated, and the review also concluded that there was a lack of evaluation of the cross‐cultural validity of the instruments.

A systematic review of instruments measuring cyber dating violence among adolescents using the COSMIN checklist found similar findings on the evaluation of instrument development and content validity in which the studies reported poorly on the item selection process, lack of involvement of the target population or professionals and insufficient information on the number of items, recall period and response options (Soto & Ibabe, 2022). Internal consistency and structural validity are among the psychometric properties reported in the literature (Rodríguez‐deArriba et al., 2021).

## 8 RECOMMENDATIONS

The review findings reveal a wide and diverse range of instruments researchers use to gauge AOP in community and institutional settings. While numerous instruments cover a broad spectrum of abuse types, others focus exclusively on specific forms of mistreatment. Despite generally lacking robust psychometric properties and limited‐quality evidence, researchers can still adopt and modify existing instruments. Researchers can refine the instrument's validity and reliability by incorporating adjustments, including various methodological and procedural changes tailored to specific research contexts and settings.

Researchers planning to adopt pre‐existing instruments or develop new measurement instruments to measure prevalence can enhance their instruments by following COSMIN guidelines. These guidelines provide a robust framework for ensuring the quality and reliability of measurement instruments (Mokkink et al., 2018; Prinsen et al., 2018; Terwee et al., 2018). As COSMIN guidelines recommend, it is crucial to ensure that the chosen instrument has undergone rigorous validation procedures, including evaluation of psychometric properties, such as reliability, validity, responsiveness, and interpretability, specifically in assessing AOP prevalence. Researchers should carefully select instruments with strong evidence supporting their psychometric properties in similar populations or contexts or conduct validation studies to ensure the instrument's suitability for accurately and reliably measuring AOP prevalence.

Transparent reporting of measurement properties is essential in developing or adopting instruments for measuring AOP prevalence. Researchers should provide detailed information on the instrument development or selection methods and the validation procedures employed, including sample characteristics, statistical analyses, and results. Transparent reporting facilitates understanding and interpretation of the instrument's strengths and limitations. This would allow other researchers and stakeholders to determine its suitability and contribute to the ongoing refinement of measurement practices in this domain.

Engaging stakeholders, including older people, caregivers, healthcare professionals, and policymakers, is paramount in developing and adopting instruments to measure AOP prevalence. Their input can ensure that the instrument adequately captures the nuances of AOP experiences, addresses relevant cultural or contextual factors, and meets end‐users' needs in various settings. Additionally, stakeholder involvement can help identify potential biases or limitations in the measurement process and foster greater acceptance and uptake of the instrument within the broader community.

Researchers should commit to ongoing evaluation and refinement of measurement instruments for AOP prevalence to ensure their continued relevance and effectiveness. This involves monitoring emerging evidence, soliciting stakeholder feedback, and actively seeking opportunities for improvement based on new insights or evolving needs. By embracing a dynamic and iterative approach to instrument development and validation, researchers can contribute to advancing measurement practices in AOP prevalence assessment, ultimately leading to more accurate, reliable, and comprehensive data to inform prevention and intervention efforts.

The comprehensive groundwork laid by previous researchers serves as a solid foundation for refining existing AOP measurement instruments, aiming to produce a new instrument that boasts worldwide validity and reliability. Previous research efforts have provided invaluable insights into the complexities of AOP measurement, contributing to the development and validation of existing instruments. Building upon this rich body of work, establishing a standardised measurement tool is imperative for accurately assessing the prevalence of AOP on a global scale. Conducting content or item analyses on instruments whose content validity and psychometric properties have been rigorously assessed across various countries and cultural contexts constitutes a pivotal stride toward crafting an all‐encompassing item set that addresses every facet and form of abuse. Through content analysis, existing items can undergo enhancement, while their psychometric properties can be systematically evaluated on a global scale, ensuring robustness and applicability across diverse populations and settings.

## 9 AUTHORS' CONCLUSIONS

This marks the inaugural systematic review employing the COSMIN criteria for evaluating psychometric properties of AOP measurement instruments used in community and institutional settings. Our findings included 53 studies that reported 46 distinct original instruments and 62 studies on 22 modified instruments. Notably, the methodological quality of evidence for most instruments is limited and deficient. Although content validity is regarded as pivotal in assessing an instrument's validity, many instruments have not comprehensively evaluated their content validity. The methodological quality of the studies and evidence pertaining to each psychometric property are lacking.

Considering the notably low quality of evidence observed throughout this review, we could not make any specific recommendations for a particular measurement instrument to gauge the prevalence of AOP within the community and institutional settings. It is worth noting that some researchers have undertaken redundant evaluations of the same instrument's psychometric properties, such as internal consistency. While replication is vital in providing more robust evidence for establishing the reliability and validity of the instrument, it is essential to emphasise that without improved methodologies or the introduction of novel insights, these redundant assessments may lead to inefficiencies in resource allocation and potentially impede progress in the field. A myopic focus on specific psychometric attributes, such as internal consistency, and neglecting other vital dimensions like responsiveness may result in an incomplete understanding of the instrument's overall performance. Additionally, certain instruments were initially developed for screening purposes, featuring items that solely assess risk indicators rather than directly measuring the occurrence of abuse. This limitation may compromise the validity and reliability of the instruments in accurately estimating AOP prevalence, potentially leading to imprecise estimations of the true magnitude of AOP. Furthermore, other issues, such as inconsistent cut‐off values for defining abuse cases, the unidimensional nature of some abuse measurement instruments lacking comprehensive multidomain coverage, and inadequate details regarding the translational process, should be considered when selecting an appropriate measurement instrument.

The large number of studies included in this review underscores the proliferation of AOP measurement instruments developed and utilised by researchers in this field. However, systematic evidence of their validity and reliability remains scarce.

### 9.1 Implications for practice and policy

Researchers seeking to choose a suitable AOP measurement instrument should consider both its psychometric properties and content to ascertain its compatibility with the goals and objectives of their research. Each instrument evaluated in this study emphasised distinct aspects of AOP. Table 2 summarises the non‐psychometric content within the instruments, which could be crucial in the selection process.

### 9.2 Implications for research

Future research should establish the reliability and validity of the most commonly employed instruments for measuring AOP. Moreover, extensive research is needed to develop a comprehensive measurement instrument encompassing various AOP subscales. This can be achieved by incorporating existing items from established instruments or developing new relevant items. The psychometric properties of this new measurement instrument need to be comprehensively evaluated using an appropriate methodological approach, as recommended by the COSMIN guideline.

## CONTRIBUTIONS OF AUTHORS

Content: Fadzilah Hanum Mohd Mydin, Choo Wan Yuen, Christopher Mikton, Noran Naqiah Hairi, Farizah Hairi, Raudah Mohd Yunus, Yongjie Yon, Marie Beaulieu, Amanda Phelan.

Systematic review methods: Fadzilah Hanum Mohd Mydin, Choo Wan Yuen, Christopher Mikton, Yongjie Yon, Noran Naqiah Hairi, Farizah Hairi, Raudah Mohd Yunus.

Statistical analysis: Choo Wan Yuen, Aja Murray, Fadzilah Hanum Mohd Mydin.

Information retrieval: Ranita Hisham Shanmugam.

## DECLARATIONS OF INTEREST

Choo Wan Yuen, Noran Naqiah Hairi, Christopher Mikton, Yongjie Yon, Amanda Phelan, and Raudah Mohd Yunus are involved in the design, conduct and publication of a study potentially eligible for the review.

Fadzilah Hanum Mohd Mydin, Marie Beaulieu, Aja Murray, and Ranita Hisham Shanmugam have no conflict of interest to declare.

## SOURCE OF SUPPORT

This review is made possible with financial support from the following funders:

Ministry of Higher Education Fundamental Research Grant Scheme, Malaysia (FRGS/1/2022/SKK05/UM/01/1) awarded to the principal investigator, Choo Wan Yuen.

World Health Organization Educational Grant, Intervention Accelerator for Abuse of Older People (IF033‐2023).

## DIFFERENCES BETWEEN PROTOCOL AND REVIEW

We changed the term ‘elder abuse and neglect’ to ‘abuse of older person’ in the title of the review and text.

## Supporting information

Supporting information.
