# Supplementary material for: Psychometric properties of instruments for measuring abuse of older people in community and institutional settings: A systematic review
Source: Campbell Syst Rev. 2024 Aug 29;20(3):e1419. doi: 10.1002/cl2.1419 (PMC11358705; doi:10.1002/cl2.1419)
Supplement: Supplementary file 1 — Supporting information. [file CL2-20-e1419-s001.docx]

# Appendices

#### Appendix 1: Definition of elder abuse subtypes

| **Abuse subtype** | **Definition** |
| --- | --- |
| Physical | Elder experiences illness, pain, injury, functional impairment, distress, or death due to the intentional use of physical force and includes acts such as hitting, kicking, pushing, slapping, and burning. |
| Emotional | Verbal or nonverbal behaviours that inflict anguish, mental pain, fear, or distress on an older adult. Examples include humiliation or disrespect, verbal and non-verbal threats, harassment, and geographic or interpersonal isolation. |
| Financial | Illegal, unauthorised, or improper use of an elder's money, benefits, belongings, property, or assets for the benefit of someone other than the older adult. |
| Sexual | Forced or unwanted sexual interaction of any kind with an older adult. This action may include unwanted sexual contact, penetration, or non-contact acts such as sexual harassment. |
| Neglect | Failure to meet an older adult's basic needs. These needs include food, water, shelter, clothing, hygiene, and essential medical care. |

#### Appendix 2: COSMIN definitions of domains, measurement properties, and aspects of measurement properties (Mokkink et al., 2010)

| **Domain** | **Measurement property** | **Aspect of a measurement**  **property** | **Definition** |
| --- | --- | --- | --- |
| Reliability |  |  | The degree to which the measurement is free from measurement error. |
|  | Internal consistency |  | The degree of the  interrelatedness among the items. |
|  | Reliability |  | The proportion of the total variance in the measurements is due to 'true’† differences between patients. |
|  | Measurement error |  | The systematic and random error of a patient's score that is not attributed to true changes in the construct is to be measured. |
| Validity |  |  | The degree to which a PROM measures the construct(s) it purports to measure. |
|  | Content validity |  | The degree to which the content of a PROM is an adequate reflection of the construct to be measured. |
|  |  | Face validity | The degree to which (the items of) a PROM look like an adequate reflection of the construct to be measured. |
|  | validity |  | The degree to which the scores of a PROM are consistent with hypotheses (for instance, concerning internal relationships, relationships to scores of other instruments, or differences  between relevant groups) based on the assumption that the PROM  validly measures the construct to be measured. |
|  |  | Structural  validity | The degree to which the scores of a PROM are an adequate reflection of the dimensionality of the construct to be measured. |
|  |  | Hypotheses  testing | Idem construct validity |
|  |  | Cross-cultural  validity | The degree to which the  performance of the items on a translated or culturally adapted PROM is an adequate reflection of the performance of the items of the original version of the PROM. |
|  | Criterion  validity |  | The degree to which the scores of a PROM are an adequate reflection of a 'gold standard'. |
| Responsiveness |  |  | The ability of a PROM to detect change over time in the construct is to be measured. |
|  | Responsiveness |  | Idem responsiveness |
| Interpretability* |  |  | Interpretability is the degree to which one can assign qualitative meaning ‐ that is, clinical or commonly understood connotations – to a PROM's quantitative scores or change in scores. |

The word 'true' must be seen in the context of the CTT, which states that any observation comprises two components – a true score and an error associated with the observation.

'True' is the average score obtained if the scale were given an infinite number of times. It refers only to the consistency of the score and not to its accuracy

* Interpretability is not considered a measurement property but an important characteristic of a measurement instrument

#### Appendix 3: Search strategy

| **No** | **Databases** | **Search strategy** |
| --- | --- | --- |
| 1 | Pubmed | **(((("elder* abuse*"[Title/Abstract:~3] OR "elder* neglect"[Title/Abstract:~3] OR "elder* mistreatment"[Title/Abstract:~3] OR "elder* maltreatment"[Title/Abstract:~3] OR elder abuse[MeSH Terms])))) AND ((((( psychometr*[Title/Abstract] OR "outcome assessment"[Title/Abstract] OR "observer variation"[Title/Abstract] OR reproducib*[Title/Abstract] OR reliab*[Title/Abstract] OR unreliab*[Title/Abstract] OR valid*[Title/Abstract] OR "coefficient of variation"[Title/Abstract] OR coefficient[Title/Abstract] OR homogeneity[Title/Abstract] OR homogeneous[Title/Abstract] OR "internal consistency"[Title/Abstract] OR "cronbach alpha"[Title/Abstract] OR "cronbach alphas"[Title/Abstract] OR "items correlation*"[Title/Abstract] OR "items selection*"[Title/Abstract] OR "item reduction*"[Title/Abstract] OR "test retest"[Title/Abstract] OR stability[Title/Abstract] OR interrater[Title/Abstract] OR inter-rater[Title/Abstract] OR intrarater[Title/Abstract] OR intra-rater[Title/Abstract] OR intertester[Title/Abstract] OR inter-tester[Title/Abstract] OR intratester[Title/Abstract] OR intra-tester[Title/Abstract] OR interobserver[Title/Abstract] OR inter-observer[Title/Abstract] OR intraobserver[Title/Abstract] OR intra-observer[Title/Abstract] OR interexaminer[Title/Abstract] OR inter-examiner[Title/Abstract] OR intraexaminer[Title/Abstract] OR intra-examiner[Title/Abstract] OR interindividual[Title/Abstract] OR inter-individual[Title/Abstract] OR intraindividual[Title/Abstract] OR intra-individual[Title/Abstract] OR interparticipant[Title/Abstract] OR inter-participant[Title/Abstract] OR intraparticipant[Title/Abstract] OR intra-participant[Title/Abstract] OR kappa[Title/Abstract] OR kappa's[Title/Abstract] OR kappas[Title/Abstract] OR "repeated measure"[Title/Abstract] OR "repeated measures"[Title/Abstract] OR "repeated findings"[Title/Abstract] OR "repeated results"[Title/Abstract] OR "repeated test"[Title/Abstract] OR "repeated tests"[Title/Abstract] OR generaliza*[Title/Abstract] OR generalisa*[Title/Abstract] OR concordance[Title/Abstract] OR "intraclass-correlation"[Title/Abstract] OR discriminative[Title/Abstract] OR "known group"[Title/Abstract] OR "factor analysis"[Title/Abstract] OR "factor analyses"[Title/Abstract] OR "factor structure"[Title/Abstract] OR "factor structures"[Title/Abstract] OR dimension*[Title/Abstract] OR subscale*[Title/Abstract] OR "multitrait analysis"[Title/Abstract] OR "multitrait analyses"[Title/Abstract] OR "scaling analysis"[Title/Abstract] OR "scaling analyses"[Title/Abstract] OR "item discriminant"[Title/Abstract] OR "interscale correlation*"[Title/Abstract] OR error[Title/Abstract] OR errors[Title/Abstract] OR "individual variability"[Title/Abstract] OR "interval variability"[Title/Abstract] OR "rate variability"[Title/Abstract] OR "variability analysis"[Title/Abstract] OR "variability values"[Title/Abstract] OR "uncertainty measurement"[Title/Abstract] OR "standard error of measurement"[Title/Abstract] OR sensitiv*[Title/Abstract] OR responsive*[Title/Abstract] OR "limit detection"[Title/Abstract] OR "minimal detectable concentration"[Title/Abstract] OR interpretab*[Title/Abstract] OR "Minimal important"[Title/Abstract] OR "minimally important"[Title/Abstract] OR "clinical important"[Title/Abstract] OR "clinically important"[Title/Abstract] OR "Minimal significant"[Title/Abstract] OR "Minimally significant"[Title/Abstract] OR "clinical significant"[Title/Abstract] OR "clinically significant"[Title/Abstract] OR "minimal detectable"[Title/Abstract] OR "minimally detectable"[Title/Abstract] OR "clinical detectable"[Title/Abstract] OR "clinically detectable"[Title/Abstract] OR Change[Title/Abstract] OR difference[Title/Abstract] OR "meaningful change"[Title/Abstract] OR "ceiling effect"[Title/Abstract] OR "floor effect"[Title/Abstract] OR "Item response model"[Title/Abstract] OR IRT[Title/Abstract] OR Rasch[Title/Abstract] OR "Differential item functioning"[Title/Abstract] OR DIF[Title/Abstract] OR "computer adaptive testing"[Title/Abstract] OR "item bank"[Title/Abstract] OR "cross-cultural equivalence"[Title/Abstract] OR "screening tool"[Title/Abstract] OR "screening assessment"[Title/Abstract] OR assessment[Title/Abstract] OR "assessment tool"[Title/Abstract] OR screening[Title/Abstract] OR "appraisal tool"[Title/Abstract])) OR ((((((psychometrics[MeSH Terms]) OR (assessment, outcome health care[MeSH Terms])) OR (observer variation[MeSH Terms])) OR (health status indicator[MeSH Terms])) OR (reproducibility of results[MeSH Terms])) OR (discriminant analysis[MeSH Terms])))))** |
| 2 | CINAHL | TI ( "elder* abuse*" OR "elder* neglect" OR "elder* mistreatment" OR "elder* maltreatment" ) OR AB ( "elder* abuse*" OR "elder* neglect" OR "elder* mistreatment" OR "elder* maltreatment" ) AND ( ( SU ( instrumentation OR methods ) OR PT ( "Validation Studies" OR "Comparative Study" ) OR TI ( psychometr* OR "outcome assessment" OR "observer variation" OR reproducib* OR reliab* OR unreliab* OR valid* OR "coefficient of variation" OR coefficient OR homogeneity OR homogeneous OR "internal consistency" OR "cronbach* alpha" OR "cronbach alphas" OR "items correlation*" OR "items selection*" OR "item reduction*" OR "test-retest" OR "test retest" OR reliab* OR "test OR retest" OR stability OR interrater OR inter-rater OR intrarater OR intra-rater OR intertester OR inter-tester OR intratester OR intra-tester OR interobserver OR inter-observer OR intraobserver OR intra-observer OR interexaminer OR inter-examiner OR intraexaminer OR intra-examiner OR interindividual OR inter-individual OR intraindividual OR intra-individual OR interparticipant OR inter-participant OR intraparticipant OR intra-participant OR kappa OR kappa's OR kappas OR “replica* measure” OR “replica* measures” OR “replica* findings” OR “replica* result” OR “replica* test” OR “replica* tests” OR “repeated measure” OR “repeated measures” OR “repeated findings” OR “repeated results” OR “repeated test” OR “repeated tests” OR generaliza* OR generalisa* OR concordance OR "intraclass-correlation" OR discriminative OR "known group" OR "factor analysis" OR "factor analyses" OR "factor structure" OR "factor structures" OR dimension* OR subscale* OR "multitrait analysis" OR "multitrait analyses" OR "scaling analysis" OR "scaling analyses" OR "item discriminant" OR "interscale correlation*" OR error OR errors OR "individual variability" OR "interval variability" OR "rate variability" OR "variability analysis" OR "variability values" OR "uncertainty measurement" OR "uncertainty measuring" OR "standard error of measurement" OR sensitiv* OR responsive* OR "limit detection" OR "minimal detectable concentration" OR interpretab* OR “Minimal important” OR “minimally important” OR “clinical important” OR “clinically important” OR “Minimal significant” OR “Minimally significant” OR “clinical significant” OR “clinically significant” OR “minimal detectable” OR “minimally detectable” OR “clinical detectable” OR “clinically detectable” OR Change OR difference OR “small* real” OR “small detectable” OR “small* change” OR “small* difference” OR "meaningful change" OR "ceiling effect" OR "floor effect" OR "Item response model" OR IRT OR Rasch OR "Differential item functioning" OR DIF OR "computer adaptive testing" OR "item bank" OR "cross-cultural equivalence" OR “screening tool”) OR AB ( psychometr* OR "outcome assessment" OR "observer variation" OR reproducib* OR reliab* OR unreliab* OR valid* OR "coefficient of variation" OR coefficient OR homogeneity OR homogeneous OR "internal consistency" OR "cronbach* alpha" OR "cronbach alphas" OR "items correlation*" OR "items selection*" OR "item reduction*" OR "test-retest" OR "test retest" OR reliab* OR "test OR retest" OR stability OR interrater OR inter-rater OR intrarater OR intra-rater OR intertester OR inter-tester OR intratester OR intra-tester OR interobserver OR inter-observer OR intraobserver OR intra-observer OR interexaminer OR inter-examiner OR intraexaminer OR intra-examiner OR interindividual OR inter-individual OR intraindividual OR intra-individual OR interparticipant OR inter-participant OR intraparticipant OR intra-participant OR kappa OR kappa's OR kappas OR “replica* measure” OR “replica* measures” OR “replica* findings” OR “replica* result” OR “replica* test” OR “replica* tests” OR repeated measure” OR “repeated measures” OR “repeated findings” OR “repeated results” OR “repeated test” OR “repeated tests” OR generaliza* OR generalisa* OR concordance OR "intraclass-correlation" OR discriminative OR "known group" OR "factor analysis" OR "factor analyses" OR "factor structure" OR "factor structures" OR dimension* OR subscale* OR "multitrait analysis" OR "multitrait analyses" OR "scaling analysis" OR "scaling analyses" OR "item discriminant" OR "interscale correlation*" OR error OR errors OR "individual variability" OR "interval variability" OR "rate variability" OR OR "variability analysis" OR "variability values" OR "uncertainty measurement" OR "uncertainty measuring" OR "standard error of measurement" OR sensitiv* OR responsive* OR "limit detection" OR "minimal detectable concentration" OR interpretab* OR “Minimal important” OR “minimally important” OR “clinical important” OR “clinically important” OR “Minimal significant” OR “Minimally significant” OR “clinical significant” OR “clinically significant” OR “minimal detectable” OR “minimally detectable” OR “clinical detectable” OR “clinically detectable” OR Change OR difference OR “small* real” OR “small detectable” OR “small* change” OR “small* difference” OR "meaningful change" OR "ceiling effect" OR "floor effect" OR "Item response model" OR IRT OR Rasch OR "Differential item functioning" OR DIF OR "computer adaptive testing" OR "item bank" OR "cross-cultural equivalence" OR “screening tool”) OR TX ( clinimetr* OR clinometr* OR "outcome measure*" OR agreement OR precision OR imprecision OR "precise values" OR repeatab* ) ) OR ( MM "Psychometrics" OR MM "Outcome Assessment, Health Care" OR (MM "Observer Variation") OR MM "Health Status Indicators" OR MM "Reproducibility of Results" OR MM "Discriminant Analysis" ) ) ) ) |
| 3 | Scopus | ( ( TITLE ( ( psychometr* OR "outcome assessment" OR reproducib* OR reliab* OR unreliab* OR valid* OR "screening tool" OR "screening assessment" OR assessment OR "assessment tool" OR screening OR "appraisal tool" ) ) OR ABS ( ( psychometr* OR "outcome assessment" OR reproducib* OR reliab* OR unreliab* OR valid* OR "screening tool" OR "screening assessment" OR assessment OR "assessment tool" OR screening OR "appraisal tool" ) ) ) ) AND ( ( TITLE ( ( "elder* abuse*" OR "elder* neglect" OR "elder* mistreatment" OR "elder* maltreatment" ) ) OR ABS ( ( "elder* abuse*" OR "elder* neglect" OR "elder* mistreatment" OR "elder* maltreatment" ) ) ) ) AND ( LIMIT-TO ( DOCTYPE , "ar" ) OR LIMIT-TO ( DOCTYPE , "re" ) OR LIMIT-TO ( DOCTYPE , "cp" ) ) |
| 4 | LILACS | ( "elder* abuse*" OR "elder* neglect" OR "elder* mistreatment" OR "elder* maltreatment") AND ("psychometric properties" OR "psychometr*" OR "screening tool" OR tool OR "outcome assessment" OR assessment ) |
| 5 | SciELO | (ti:("elder* abuse*" OR "elder* neglect" OR "elder* mistreatment" OR "elder* maltreatment")) OR (ab:("elder* abuse*" OR "elder* neglect" OR "elder* mistreatment" OR "elder* maltreatment")) AND (ti:("psychometric properties" OR "psychometr*" OR "screening tool" OR tool OR "outcome assessment" OR assessment)) OR (ab:("psychometric properties" OR "psychometr*" OR "screening tool" OR tool OR "outcome assessment" OR assessment)) |
| 6 | AgeLine | TI ("elder* abuse*" OR "elder* neglect" OR "elder* mistreatment" OR "elder* maltreatment") OR AB ( "elder* abuse*" OR "elder* neglect" OR "elder* mistreatment" OR "elder* maltreatment" ) AND ( ( SU ( instrumentation OR methods) OR PT ("Validation Studies" OR "Comparative Study" ) OR TI ( psychometr* OR "outcome assessment" OR "observer variation" OR reproducib* OR reliab* OR unreliab* OR valid* OR "coefficient of variation" OR coefficient OR homogeneity OR homogeneous OR "internal consistency" OR "cronbach* alpha" OR "cronbach alphas" OR "items correlation*" OR "items selection*" OR "item reduction*" OR "test-retest" OR "test retest" OR reliab* OR "test OR retest" OR stability OR interrater OR inter-rater OR intrarater OR intra-rater OR intertester OR inter-tester OR intratester OR intra-tester OR interobserver OR inter-observer OR intraobserver OR intra-observer OR interexaminer OR inter-examiner OR intraexaminer OR intra-examiner OR interindividual OR inter-individual OR intraindividual OR intra-individual OR interparticipant OR inter-participant OR intraparticipant OR intra-participant OR kappa OR kappa's OR kappas OR “replica* measure” OR “replica* measures” OR “replica* findings” OR “replica* result” OR “replica* test” OR “replica* tests” OR “repeated measure” OR “repeated measures” OR “repeated findings” OR “repeated results” OR “repeated test” OR “repeated tests” OR generaliza* OR generalisa* OR concordance OR "intraclass-correlation" OR discriminative OR "known group" OR "factor analysis" OR "factor analyses" OR "factor structure" OR "factor structures" OR dimension* OR subscale* OR "multitrait analysis" OR "multitrait analyses" OR "scaling analysis" OR "scaling analyses" OR "item discriminant" OR "interscale correlation*" OR error OR errors OR "individual variability" OR "interval variability" OR "rate variability" OR "variability analysis" OR "variability values" OR "uncertainty measurement" OR "uncertainty measuring" OR "standard error of measurement" OR sensitiv* OR responsive* OR "limit detection" OR "minimal detectable concentration" OR interpretab* OR “Minimal important” OR “minimally important” OR “clinical important” OR “clinically important” OR “Minimal significant” OR “Minimally significant” OR “clinical significant” OR “clinically significant” OR “minimal detectable” OR “minimally detectable” OR “clinical detectable” OR “clinically detectable” OR Change OR difference OR “small* real” OR “small detectable” OR “small* change” OR “small* difference” OR "meaningful change" OR "ceiling effect" OR "floor effect" OR "Item response model" OR IRT OR Rasch OR "Differential item functioning" OR DIF OR "computer adaptive testing" OR "item bank" OR "cross-cultural equivalence" OR “screening tool”) OR AB ( psychometr* OR "outcome assessment" OR "observer variation" OR reproducib* OR reliab* OR unreliab* OR valid* OR "coefficient of variation" OR coefficient OR homogeneity OR homogeneous OR "internal consistency" OR "cronbach* alpha" OR "cronbach alphas" OR "items correlation*" OR "items selection*" OR "item reduction*" OR "test-retest" OR "test retest" OR reliab* OR "test OR retest" OR stability OR interrater OR inter-rater OR intrarater OR intra-rater OR intertester OR inter-tester OR intratester OR intra-tester OR interobserver OR inter-observer OR intraobserver OR intra-observer OR interexaminer OR inter-examiner OR intraexaminer OR intra-examiner OR interindividual OR inter-individual OR intraindividual OR intra-individual OR interparticipant OR inter-participant OR intraparticipant OR intra-participant OR kappa OR kappa's OR kappas OR “replica* measure” OR “replica* measures” OR “replica* findings” OR “replica* result” OR “replica* test” OR “replica* tests” OR repeated measure” OR “repeated measures” OR “repeated findings” OR “repeated results” OR “repeated test” OR “repeated tests” OR generaliza* OR generalisa* OR concordance OR "intraclass-correlation" OR discriminative OR "known group" OR "factor analysis" OR "factor analyses" OR "factor structure" OR "factor structures" OR dimension* OR subscale* OR "multitrait analysis" OR "multitrait analyses" OR "scaling analysis" OR "scaling analyses" OR "item discriminant" OR "interscale correlation*" OR error OR errors OR "individual variability" OR "interval variability" OR "rate variability" OR OR "variability analysis" OR "variability values" OR "uncertainty measurement" OR "uncertainty measuring" OR "standard error of measurement" OR sensitiv* OR responsive* OR "limit detection" OR "minimal detectable concentration" OR interpretab* OR “Minimal important” OR “minimally important” OR “clinical important” OR “clinically important” OR “Minimal significant” OR “Minimally significant” OR “clinical significant” OR “clinically significant” OR “minimal detectable” OR “minimally detectable” OR “clinical detectable” OR “clinically detectable” OR Change OR difference OR “small* real” OR “small detectable” OR “small* change” OR “small* difference” OR "meaningful change" OR "ceiling effect" OR "floor effect" OR "Item response model" OR IRT OR Rasch OR "Differential item functioning" OR DIF OR "computer adaptive testing" OR "item bank" OR "cross-cultural equivalence" OR “screening tool”) OR TX ( clinimetr* OR clinometr* OR "outcome measure*" OR agreement OR precision OR imprecision OR "precise values" OR repeatab* ) ) OR ( MM "Psychometrics" OR MM "Outcome Assessment, Health Care" OR (MM "Observer Variation") OR MM "Health Status Indicators" OR MM "Reproducibility of Results" OR MM "Discriminant Analysis" ) ) ) ) |
| 7 | ASSIA | (ab(("elder* abuse*" OR "elder* neglect" OR "elder* mistreatment" OR "elder* maltreatment"))) OR (ti (("elder* abuse*" OR "elder* neglect" OR "elder* mistreatment" OR "elder* maltreatment"))) OR [mainsubject.Exact("elder abuse")](https://www.proquest.com/recentsearches.recentsearchtabview.recentsearchesgridview.scrolledrecentsearchlist.checkdbssearchlink:rerunsearch/118B2EF3CF1E4146PQ/None?site=pqdtglobal&t:ac=RecentSearches) AND PEER(yes)) AND (ti((psychometr* OR "outcome assessment" OR reproducib* OR reliab* OR unreliab* OR valid* OR "screening tool" OR "screening assessment" OR "assessment tool" OR "appraisal tool")) OR (ti((psychometr* OR "outcome assessment" OR reproducib* OR reliab* OR unreliab* OR valid* OR "screening tool" OR "screening assessment" OR "assessment tool" OR "appraisal tool")) AND PEER(yes)) |
| 8 | CNKI | "elder* abuse" |
| 9 | EMBASE | #7 #3 AND #6  #6 #4 OR #5  #5 'psychometry'/exp OR 'outcome assessment'/exp OR 'observer variation'/exp OR 'health status indicator'/exp OR 'reproducibility'/exp OR 'discriminant analysis'/exp  #4 psychometr*:ab,ti OR 'outcome assessment':ab,ti OR 'observer variation':ab,ti OR reproducib*:ab,ti OR reliab*:ab,ti OR unreliab*:ab,ti OR valid*:ab,ti OR 'coefficient of variation':ab,ti OR coefficient:ab,ti OR homogeneity:ab,ti OR homogeneous:ab,ti OR 'internal consistency':ab,ti OR 'cronbach alpha':ab,ti OR 'cronbach alphas':ab,ti OR 'items correlation*':ab,ti OR 'items selection*':ab,ti OR 'item reduction*':ab,ti OR 'test retest':ab,ti OR stability:ab,ti OR interrater:ab,ti OR 'inter rater':ab,ti OR intrarater:ab,ti OR 'intra rater':ab,ti OR intertester:ab,ti OR 'inter tester':ab,ti OR intratester:ab,ti OR 'intra tester':ab,ti OR interobserver:ab,ti OR 'inter observer':ab,ti OR intraobserver:ab,ti OR 'intra observer':ab,ti OR interexaminer:ab,ti OR 'inter examiner':ab,ti OR intraexaminer:ab,ti OR 'intra examiner':ab,ti OR interindividual:ab,ti OR 'inter individual':ab,ti OR intraindividual:ab,ti OR 'intra individual':ab,ti OR interparticipant:ab,ti OR 'inter participant':ab,ti OR intraparticipant:ab,ti OR 'intra participant':ab,ti OR kappa:ab,ti OR kappas:ab,ti OR 'repeated measure':ab,ti OR 'repeated measures':ab,ti OR 'repeated findings':ab,ti OR 'repeated results':ab,ti OR 'repeated test':ab,ti OR 'repeated tests':ab,ti OR generaliza*:ab,ti OR generalisa*:ab,ti OR concordance:ab,ti OR 'intraclass correlation':ab,ti OR discriminative:ab,ti OR 'known group':ab,ti OR 'factor analysis':ab,ti OR 'factor analyses':ab,ti OR 'factor structure':ab,ti OR 'factor structures':ab,ti OR dimension*:ab,ti OR subscale*:ab,ti OR 'multitrait analysis':ab,ti OR 'multitrait analyses':ab,ti OR 'scaling analysis':ab,ti OR 'scaling analyses':ab,ti OR 'item discriminant':ab,ti OR 'interscale correlation*':ab,ti OR error:ab,ti OR errors:ab,ti OR 'individual variability':ab,ti OR 'interval variability':ab,ti OR 'rate variability':ab,ti OR 'variability analysis':ab,ti OR 'variability values':ab,ti OR 'uncertainty measurement':ab,ti OR 'standard error of measurement':ab,ti OR sensitiv*:ab,ti OR responsive*:ab,ti OR 'limit detection':ab,ti OR 'minimal detectable concentration':ab,ti OR interpretab*:ab,ti OR 'minimal important':ab,ti OR 'minimally important':ab,ti OR 'clinical important':ab,ti OR 'clinically important':ab,ti OR 'minimal significant':ab,ti OR 'minimally significant':ab,ti OR 'clinical significant':ab,ti OR 'clinically significant':ab,ti OR 'minimal detectable':ab,ti OR 'minimally detectable':ab,ti OR 'clinical detectable':ab,ti OR 'clinically detectable':ab,ti OR change:ab,ti OR difference:ab,ti OR 'meaningful change':ab,ti OR 'ceiling effect':ab,ti OR 'floor effect':ab,ti OR 'item response model':ab,ti OR irt:ab,ti OR rasch:ab,ti OR 'differential item functioning':ab,ti OR dif:ab,ti OR 'computer adaptive testing':ab,ti OR 'item bank':ab,ti OR 'cross-cultural equivalence':ab,ti OR 'screening tool':ab,ti OR 'screening assessment':ab,ti OR assessment:ab,ti OR 'assessment tool':ab,ti OR screening:ab,ti OR 'appraisal tool':ab,ti  #3 #1 OR #2  #2 'elder abuse'/exp  #1 'elder* abuse*':ab,ti OR 'elder* neglect':ab,ti OR 'elder* mistreatment':ab,ti OR 'elder* maltreatment':ab,ti |
| 10 | Google Scholar | ("elder* abuse" OR "elder* neglect" OR "elder* mistreatment" OR "elder* maltreatment") AND psychometric* OR “screening tool” OR tool) |
| 11 | Proquest | S1 - title(( "elder* abuse*" OR "elder* neglect" OR "elder* mistreatment" OR "elder* maltreatment" ) ) OR abstract(( "elder* abuse*" OR "elder* neglect" OR "elder* mistreatment" OR "elder* maltreatment" ) )  S2 - title((((psychometr* OR "outcome assessment" OR reproducib* OR reliab* OR unreliab* OR valid* OR "screening tool" OR "screening assessment" OR "assessment tool" OR "appraisal tool")))) OR abstract((((psychometr* OR "outcome assessment" OR reproducib* OR reliab* OR unreliab* OR valid* OR "screening tool" OR "screening assessment" OR "assessment tool" OR "appraisal tool"))))  S3- S1 AND S2 |
| 12 | PsycINFO | (((("elder* abuse*[Title"/ OR "elder* neglect[Title"/ OR "elder* mistreatment[Title"/ OR "elder* maltreatment[Title"/ OR exp "elder abuse"/)))) AND (((((psychometr*.ti,ab. OR "outcome assessment".ti,ab. OR "observer variation".ti,ab. OR reproducib*.ti,ab. OR reliab*.ti,ab. OR unreliab*.ti,ab. OR valid*.ti,ab. OR "coefficient of variation".ti,ab. OR coefficient.ti,ab. OR homogeneity.ti,ab. OR homogeneous.ti,ab. OR "internal consistency".ti,ab. OR "cronbach alpha".ti,ab. OR "cronbach alphas".ti,ab. OR "items correlation*".ti,ab. OR "items selection*".ti,ab. OR "item reduction*".ti,ab. OR "test retest".ti,ab. OR stability.ti,ab. OR interrater.ti,ab. OR inter-rater.ti,ab. OR intrarater.ti,ab. OR intra-rater.ti,ab. OR intertester.ti,ab. OR inter-tester.ti,ab. OR intratester.ti,ab. OR intra-tester.ti,ab. OR interobserver.ti,ab. OR inter-observer.ti,ab. OR intraobserver.ti,ab. OR intra-observer.ti,ab. OR interexaminer.ti,ab. OR inter-examiner.ti,ab. OR intraexaminer.ti,ab. OR intra-examiner.ti,ab. OR interindividual.ti,ab. OR inter-individual.ti,ab. OR intraindividual.ti,ab. OR intra-individual.ti,ab. OR interparticipant.ti,ab. OR inter-participant.ti,ab. OR intraparticipant.ti,ab. OR intra-participant.ti,ab. OR kappa.ti,ab. OR kappa's.ti,ab. OR kappas.ti,ab. OR "repeated measure".ti,ab. OR "repeated measures".ti,ab. OR "repeated findings".ti,ab. OR "repeated results".ti,ab. OR "repeated test".ti,ab. OR "repeated tests".ti,ab. OR generaliza*.ti,ab. OR generalisa*.ti,ab. OR concordance.ti,ab. OR intraclass-correlation.ti,ab. OR discriminative.ti,ab. OR "known group".ti,ab. OR "factor analysis".ti,ab. OR "factor analyses".ti,ab. OR "factor structure".ti,ab. OR "factor structures".ti,ab. OR dimension*.ti,ab. OR subscale*.ti,ab. OR "multitrait analysis".ti,ab. OR "multitrait analyses".ti,ab. OR "scaling analysis".ti,ab. OR "scaling analyses".ti,ab. OR "item discriminant".ti,ab. OR "interscale correlation*".ti,ab. OR error.ti,ab. OR errors.ti,ab. OR "individual variability".ti,ab. OR "interval variability".ti,ab. OR "rate variability".ti,ab. OR "variability analysis".ti,ab. OR "variability values".ti,ab. OR "uncertainty measurement".ti,ab. OR "standard error of measurement".ti,ab. OR sensitiv*.ti,ab. OR responsive*.ti,ab. OR "limit detection".ti,ab. OR "minimal detectable concentration".ti,ab. OR interpretab*.ti,ab. OR "Minimal important".ti,ab. OR "minimally important".ti,ab. OR "clinical important".ti,ab. OR "clinically important".ti,ab. OR "Minimal significant".ti,ab. OR "Minimally significant".ti,ab. OR "clinical significant".ti,ab. OR "clinically significant".ti,ab. OR "minimal detectable".ti,ab. OR "minimally detectable".ti,ab. OR "clinical detectable".ti,ab. OR "clinically detectable".ti,ab. OR Change.ti,ab. OR difference.ti,ab. OR "meaningful change".ti,ab. OR "ceiling effect".ti,ab. OR "floor effect".ti,ab. OR "Item response model".ti,ab. OR IRT.ti,ab. OR Rasch.ti,ab. OR "Differential item functioning".ti,ab. OR DIF.ti,ab. OR "computer adaptive testing".ti,ab. OR "item bank".ti,ab. OR "cross-cultural equivalence".ti,ab. OR "screening tool".ti,ab. OR "screening assessment".ti,ab. OR assessment.ti,ab. OR "assessment tool".ti,ab. OR screening.ti,ab. OR "appraisal tool".ti,ab.)) OR ((((((exp psychometrics/) OR (exp "assessment, outcome health care"/)) OR (exp "observer variation"/)) OR (exp "health status indicator"/)) OR (exp "reproducibility of results"/)) OR (exp "discriminant analysis"/))))) |
| 13 | Sociological Abstract | S1 - title(( "elder* abuse*" OR "elder* neglect" OR "elder* mistreatment" OR "elder* maltreatment" ) ) OR abstract(( "elder* abuse*" OR "elder* neglect" OR "elder* mistreatment" OR "elder* maltreatment" ) )  S2 - title((((psychometr* OR "outcome assessment" OR reproducib* OR reliab* OR unreliab* OR valid* OR "screening tool" OR "screening assessment" OR "assessment tool" OR "appraisal tool")))) OR abstract((((psychometr* OR "outcome assessment" OR reproducib* OR reliab* OR unreliab* OR valid* OR "screening tool" OR "screening assessment" OR "assessment tool" OR "appraisal tool"))))  S3- S1 AND S2 |
| 14 | WHO Index Medicus | “elder abuse” OR “elder neglect” |

#### Appendix 4: COSMIN Risk of Bias Checklist

| **Content validity**  Box 1: PROM Development  Box 2: Content validity |
| --- |
| **Internal structure**  Box 3: Structural validity  Box 4: Internal consistency  Box 5: Cross-cultural validity/measurement invariance |
| **Remaining measurement properties**  Box 6: Reliability  Box 7: Measurement error  Box 8: Criterion validity  Box 9: Hypotheses testing for construct validity  Box 10. Responsiveness |

#### Appendix 5: List of original and modified instruments

| **Original instrument (n=46)** | VASS-12 items  HS-EAST  CASE  GMS  *RAAL-31 items  OAPAM  *ICNH (Norwegian)  DEAQ  EPAS  UKNPS | R-REM  FPS-2 items  ABUEL  *EAS (Korea)  ATDEA  OAFEM  CPEAB  DVSQ  EMM | EVEQ  FVOW  FVS  FIVE  CHCS  *EA (Korea)  MEAS  *NAS (Ghana)  *DVE(Thailand) | *EAQ (Nepal)  *EAN (Puerto Ricans)  EAVQ  USCOACS  EMS  WHRS  * IPPA (India)  EACS  * IPVQ | *EAQ (China)  *EAQ (Japan)  *EAQ (Iran)  EAS  IEAQ-Long form  Native EAS-Short Form  *EANQ (Iran)  AAT  QEEA |
| --- | --- | --- | --- | --- | --- |
| **Modified instrument (n=22)** | mCTS-C 25 Items  mCTS-10 items  mCTS-34 items  mCTS-M 38 items  mCTS-T 18 items | mCTS-C 18 items  mCTS-C 23 items  mCTS-Verbal 12 items  mVASS-10 items  mVASS-16 items | mVASS-15 items  m-HS-EAST  mGMS (Neglect)  *mRAAL-28 items | *mRAAL-27 items  *mICNH (Norwegian)  *mNSEAN  *mNSEAN (Neglect) | mFPS-5 items  mOARS-ADL(Neglect)  m-EAQ  m-EASI |

*The original author did not name the instrument—an acronym created by the author of this review.

#### Appendix 6: List of instruments and the study setting

| **Community (n=56)** | mCTS-C 25 Items  mCTS-10 items  mCTS-34 items  mCTS-M 38 items  mCTS-T 18 items  mCTS-C 18 items  mCTS-C 23 items  mCTS-Verbal 12 items  VASS-12 items  mVASS-10 items  mVASS-16 items | mVASS-15 items  HS-EAST  m-HS-EAST  CASE  GMS  mGMS (Neglect)  *mRAAL-27 items  DEAQ  UKNPS  *mNSEAN  *mNSEAN (Neglect) | FPS-2 items  mFPS-5 items  ABUEL  ATDEA  mOARS-ADL(Neglect)  EMM  EVEQ  FVOW  FVS  FIVE  *EA (Korea) | | MEAS  *NAS (Ghana)  *DVE(Thailand)  *EAQ (Nepal)  *EAN (Puerto Ricans)  EAVQ  USCOACS  EMS  WHRS  * IPPA (India)  EACS | * IPVQ  *EAQ (China)  *EAQ (Iran)  EAS  IEAQ-Long form  Native EAS-Short Form  *EANQ (Iran)  AAT  m-EAQ  m-EASI  QEEA  OAPAM |
| --- | --- | --- | --- | --- | --- | --- |
| **Institution (n=10)** | *RAAL-31 items  *mRAAL-28 items  *ICNH (Norwegian)  *mICNH (Norwegian)  R-REM | | | *EAS (Korea)  CPEAB  DVSQ  CHCS  *EAQ (Japan) | | |
| **Community and institution (n=2)** | EPAS  OAFEM | | | | | |

#### Appendix 7: List of instruments and the study countries

| **High-income countries**  **(n=38)** | mCTS-C 25 Items  mCTS-10 items  mCTS-34 items  mCTS-C 18 items  mCTS-Verbal 12 items  mVASS-10 items  mGMS (Neglect) *RAAL- 31 items  *mRAAL-28 items  *mRAAL-27 items | OAPAM  *ICNH (Norwegian)  *mICNH (Norwegian)  UKNPS  R-REM  *mNSEAN  *mNSEAN (Neglect)  mFPS-5 items  *EAS (Korea) | CPEAB DVSQ  EMM  EVEQ  FVOW  FVS  FIVE  CHCS  *EA (Korea)  *EAN (Puerto Ricans) | EAVQ  USCOACS  WHRS  EACS  * IPVQ  *EAQ (Japan)  m-EAQ  m-EASI  QEEA |
| --- | --- | --- | --- | --- |
| **Upper-middle income countries**  **(n=11)** | mCTS-M 38 items  mCTS-C 23 items  mVASS-16 items  mVASS-15 items  FPS-2 items  ATDEA | | mOARS-ADL(Neglect)  MEAS  *DVE(Thailand)  EMS  *EAQ (China) | |
| **Low-middle income countries**  **(n=12)** | mCTS-T 18 items  m-HS-EAST  DEAQ  *NAS (Ghana)  *EAQ (Nepal)  * IPPA (India) | | *EAQ (Iran)  EAS  IEAQ-Long form  Native EAS-Short Form  *EANQ (Iran)  AAT | |
| **High, upper-middle, and low-middle-income countries**  **(n=3)** | VASS-12 items  HS-EAST  CASE | | | |
| **High and upper-middle-income countries**  **(n=2)** | ABUEL  OAFEM | | | |
| **High and low-middle-income countries**  **(n=1)** | EPAS | | | |
| **Upper-middle and low-middle income countries**  **(n=1)** | GMS | | | |

#### Appendix 8: List of subscales of abuse measured based on WHO definition.

| **Five subscales (n=18)** | mCTS-34 items  mCTS-M 38 items  m-HS-EAST  GMS  *mRAAL-27 items  DEAQ | UKNPS  *mNSEAN  ABUEL  ATDEA  FVOW  *EAQ (Nepal) | | USCOACS  *EAQ (Iran)  *EANQ (Iran)  m-EAQ  m-EASI  QEEA |
| --- | --- | --- | --- | --- |
| **Four subscales (n=14)** | mVASS-16 items  mVASS-15 items  CASE  *RAAL-31 items | *mRAAL-28 items  *ICNH(Norwegian)  *EAS (Korea)  EVEQ  *EA (Korea) | | EMS  EACS  *EAQ (China)  IEAQ-Long form  AAT |
| **Three subscales (n=10)** | mCTS-T 18 items  mCTS-C 23 items  *mICNH (Norwegian)  EMM  FVS | | CHCS  MEAS  WHRS  * IPVQ  Native EAS-Short Form | |
| **Two subscales (n=5)** | mCTS-C 25 Items  mCTS-10 items  mCTS-C 18 items  * IPPA (India)  *EAQ (Japan) | | | |
| **One subscale (n=12)** | HS-EAST  mGMS (Neglect)  OAPAM  EPAS  R-REM  *mNSEAN (Neglect) | | OAFEM  mOARS-ADL(Neglect)  CPEAB  DVSQ  FIVE  EAVQ | |
| **0 subscale (n=3)** | mCTS-Verbal 12 items  VASS-12 items  *NAS (Ghana) | | | |
| **Overall elder abuse (n=4)** | FPS-2 items  mFPS-5 items  *DVE(Thailand)  *EAN (Puerto Ricans) | | | |
| **Information on subscale not available (n=2)** | mVASS-10 items  EAS | | | |

#### Appendix 9: List of instruments and questionnaire administration

| **Older adults (n=46)** | mCTS-C 25 Items  mCTS-34 items  mCTS-M 38 items  mCTS-T 18 items  mCTS-Verbal 12 items  VASS-12 items  mVASS-10 items  mVASS-16 items  mVASS-15 items  m-HS-EAST | GMS  mGMS (Neglect)  OAPAM  DEAQ  EPAS  UKNPS  ABUEL  OAFEM  DVSQ | EMM  EVEQ  FVOW  FVS  FIVE  *EA (Korea)  MEAS  *NAS (Ghana)  *DVE(Thailand) | *EAQ (Nepal)  *EAN (Puerto Ricans)  EAVQ  USCOACS  EMS  WHRS  EACS  * IPVQ  *EAQ (China) | *EAQ (Japan)  *EAQ (Iran)  EAS  IEAQ-Long form  Native EAS-Short Form  AAT  m-EAQ  m-EASI  QEEA |
| --- | --- | --- | --- | --- | --- |
| **Caregivers (n=6)** | mCTS-10 items  mCTS-C 18 items  mCTS-C 23 items  CASE  *mRAAL-28 items  *mRAAL-27 items | | | | |
| **3^rd^ person (n=4)** | *RAAL-31 items  *ICNH(Norwegian)  *mICNH (Norwegian)  CHCS | | | | |
| **Both older adults and caregivers (n=3)** | FPS-2 items  mFPS-5 items  mOARS-ADL(Neglect) | | | | |
| **Both older adults and staff/third person (n=2)** | HS-EAST  R-REM | | | | |
| **Both caregiver and staff (n=2)** | *EAS (Korea)  CPEAB | | | | |
| **Older adults, caregivers and staff/third person (n=5)** | *mNSEAN  *mNSEAN (Neglect)  ATDEA  * IPPA (India)  *EANQ (Iran) | | | | |

#### Appendix 10: Recall period of each instrument

| **12 months (n=24**) | mCTS-34 items  mCTS-M 38 items  mCTS-T 18 items  mCTS-C 23 items  mCTS-Verbal 12 items  VASS-12 items | | mVASS-15 items  HS-EAST  m-HS-EAST  mGMS (Neglect)  OAPAM  UKNPS | | FPS-2 items  ABUEL  OAFEM  DVSQ  FVS  EAVQ | | USCOACS  EMS  EACS  AAT  mCTS-C 25 Items  GMS | |
| --- | --- | --- | --- | --- | --- | --- | --- | --- |
| **Three months (n=6)** | mCTS-10 items  *RAAL-31 items  *mRAAL-28 items  *mRAAL-27 items  CHCS  *EAQ (Nepal) | | | | | | | |
| **Four weeks (n=2)** | *mICNH (Norwegian)  *EA (Korea) | | | | | | | |
| **Two weeks (n=1**) | R-REM | | | | | | | |
| **Lifetime (n=1**) | * IPVQ | | | | | | | |
| **At the age of 55 and above (n=1**) | WHRS | | | | | | | |
| **At the age of 60 and above (n=1**) | EMM | | | | | | | |
| **No information given (n=32)** | mCTS-C 18 items  mVASS-10 items  mVASS-16 items  CASE  *ICNH(Norwegian)  DEAQ  EPAS | *mNSEAN  *mNSEAN (Neglect)  mFPS-5 items  *EAS (Korea)  ATDEA  mOARS-ADL(Neglect)  CPEAB | | EVEQ  FVOW  FIVE  MEAS  *NAS (Ghana)  *DVE(Thailand) | | *EAN (Puerto Ricans)  * IPPA (India)  *EAQ (China)  *EAQ (Japan)  *EAQ (Iran)  EAS | | IEAQ-Long form  Native EAS-Short Form  *EANQ (Iran)  m-EAQ  m-EASI  QEEA |
